# Discovery of Ureido-Substituted 4-Phenylthiazole Derivatives as IGF1R Inhibitors with Potent Antiproliferative Properties

**DOI:** 10.3390/molecules29112653

**Published:** 2024-06-04

**Authors:** Yuan Tian, Ni An, Wenru Li, Shixin Tang, Jiqi Li, He Wang, Rongjian Su, Dong Cai

**Affiliations:** 1 College of Pharmacy, Jinzhou Medical University, Jinzhou 121001, China; 2 The Key Laboratory of Molecular and Cellular Biology and Drug Development in Universities of Liaoning Province, Jinzhou Medical University, Jinzhou 121001, China

**Keywords:** HCC, anticancer, ureido of 4-phenylthiazole derivatives, inhibitors, molecular modeling

## Abstract

The existing kinase inhibitors for hepatocellular carcinoma (HCC) have conferred survival benefits but are hampered by adverse effects and drug resistance, necessitating the development of novel agents targeting distinct pathways. To discover potent new anti-HCC compounds, we leveraged scaffold hopping from Sorafenib and introduced morpholine/piperidine moieties to develop ureido-substituted 4-phenylthiazole analogs with optimized physicochemical properties and binding interactions. Notably, compound **27** exhibited potent cytotoxicity against HepG2 cells (IC_50_ = 0.62 ± 0.34 μM), significantly exceeding Sorafenib (IC_50_ = 1.62 ± 0.27 μM). Mechanistic investigations revealed that compound **27** potently inhibited HCC cell migration and colony formation, and it induced G2/M arrest and early-stage apoptosis. Kinase profiling revealed IGF1R as a key target, which compound **27** potently inhibited (76.84% at 10 μM). Molecular modeling substantiated compound **27**’s strong binding to IGF1R via multiple hydrogen bonds. Computational predictions indicate favorable drug-like properties for compound **27**. These findings provide a promising drug candidate for the treatment of HCC patients.

## 1. Introduction

Sorafenib, approved by the FDA in 2007 as the first-line standard treatment for advanced hepatocellular carcinoma (HCC), targets critical tumor angiogenesis and proliferation pathways by inhibiting VEGFR1–3, PDGFRβ, and Raf-MEK-ERK signaling [1]. Remaining the mainstay therapy for over a decade, Sorafenib offers significant survival benefits. However, its long-term efficacy poses challenges. Adverse events (AEs) such as diarrhea, weight loss, hand–foot skin reactions, alopecia, anorexia, and voice changes contribute to patient discomfort, often leading to disease progression, despite dosage adjustments or treatment cessation [2,3]. Furthermore, a notable proportion of patients (27%) in the SHARP trial did not initially respond to Sorafenib [4,5,6], indicating inherent resistance issues that hinder its effectiveness. Both primary and acquired resistance significantly curtail the survival benefits of Sorafenib. Hence, there is an urgent call for innovative therapeutic strategies or new compounds targeting distinct signaling mechanisms to diminish the likelihood of cancer cells developing resistance and to mitigate side effects. This pursuit is crucial in enhancing the prospects of successfully combating HCC [7].

**Figure 1:**
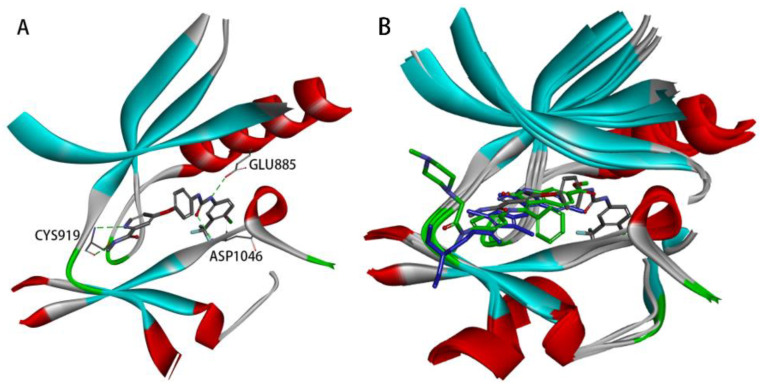
(**A**) Receptor–ligand interactions of the crystal structures of the VEGFR-2 kinase complexed with Sorafenib (PDB code: 4ASD). (**B**) Superimposition of the crystal structures of the VEGFR-2 kinase complexed with Sorafenib (PDB code: 4ASD, represented by gray carbon atoms), Sunitinib (PDB code: 4AGD, represented by blue carbon atoms), and Nintedanib (PDB code: 3C7Q, represented by green carbon atoms).

The crystal structure of VEGFR-2 in complex with Sorafenib (PDB code: 4ASD), as depicted in Figure 1A, reveals the presence of a hydrogen bond donor–acceptor pair (the ureido moiety). One hydrogen bond is formed with the side chain of a conserved GLU885 in the C-helix, and the other with the ASP1046 in the activation loop. Additionally, Sorafenib contains a hydrophobic ‘tail’ substituent (4-chloro-3-(trifluoromethyl)phenyl moiety), which interacts with the allosteric site created by the aspartate–phenylalanine–glycine (DFG)-out conformation [8,9,10]. Moreover, Sorafenib comprises a ‘head’ group (*N*-methyl-4-phenoxypicolinamide) that extends to the ATP-binding cleft, forming two hydrogen bonds with the kinase hinge residue CYS919 [10,11,12,13].

The co-crystal structures of 4ASD, 4AGD (VEGFR-2 in complex with Sunitinib), and 3C7Q (VEGFR-2 in complex with Nintedanib) were superimposed (Figure 1B). It was observed that Sunitinib and Nintedanib, both VEGFR-2 inhibitors, cannot adequately interact with the allosteric site due to their shorter backbones. Occupation of the allosteric site is a characteristic of type II inhibitors [10]. Type II inhibitors act on this site and generally have better kinase selectivity [14]. Based on the above docking analysis, it was found that the Sorafenib structure contains a hydrogen bond donor–acceptor pair (the urea moiety) and a hydrophobic ‘tail’ moiety, which are crucial for its activity and selectivity.

In this research endeavor, 4-phenylthiazole served as the fundamental structural framework for the target compounds, displaying a structural similarity to the 4-methyl-5-(pyridin-4-yl)thiazol-2-amine scaffold observed in the PI3K inhibitor Alpelisib [15]. Leveraging the principles of scaffold hopping, this scaffold was systematically substituted for the 4-phenoxypyridine moiety of Sorafenib (Figure 1B). Using Overlay Similarity with 50% steric and 50% electrostatic parameters, the molecular scaffolds or linker regions (highlighted in purple) of Sorafenib, Sunitinib, and Nintedanib were overlaid with the template of 4-phenylthiazole. The resulting similarity scores were 0.809056, 0.879794, and 0.849585, respectively. These structural similarities may influence the biological activities and pharmacokinetic properties of these drugs. Additionally, hydrophobic ureido moieties were intentionally retained to fulfill dual roles as hydrogen bond acceptors and donors. As depicted in Figure 1B, the *N*-methylacetamide fragment in Sorafenib is directed towards the kinase hinge region. This region provides ample space to accommodate the tertiary amine-containing side chains of Sunitinib and Nintedanib [16,17]. The research group has previously designed and synthesized a series of 2-amino-4-phenylthiazole scaffolds containing amide moieties that exhibit c-Met inhibitory activity. Molecular docking studies have demonstrated that the 2-morpholinoacetamido side chain in these molecules, which is oriented towards the hinge region, can lead to the identification of potent c-Met inhibitors [18].

**Figure 2:**
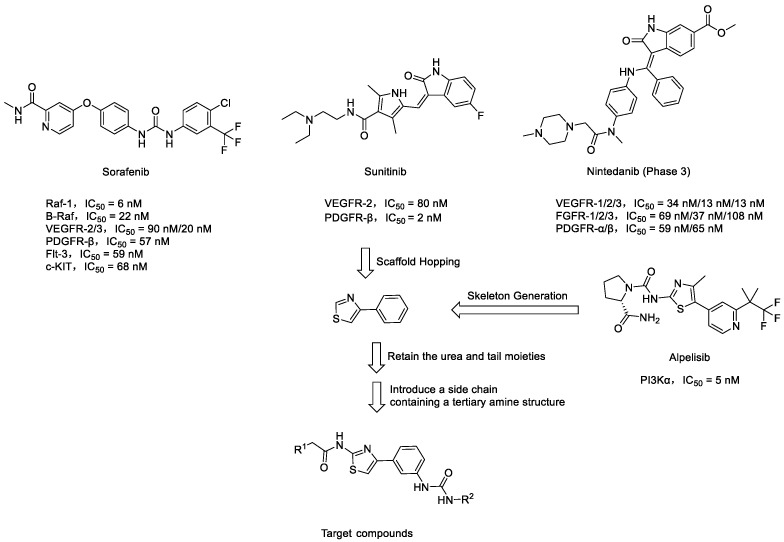
Rational design of target compounds.

To greatly enhance their water solubility and physicochemical properties, the structures of the target compounds will be modified by introducing side chains containing tertiary amine moieties (Figure 2). A similar approach was validated during the development of Imatinib, where the incorporation of the piperazine moiety was found to increase solubility and enabled the formation of an additional hydrogen bond [19,20,21].

## 2. Results and Discussion

### 2.1. Chemistry

**Scheme 1:**
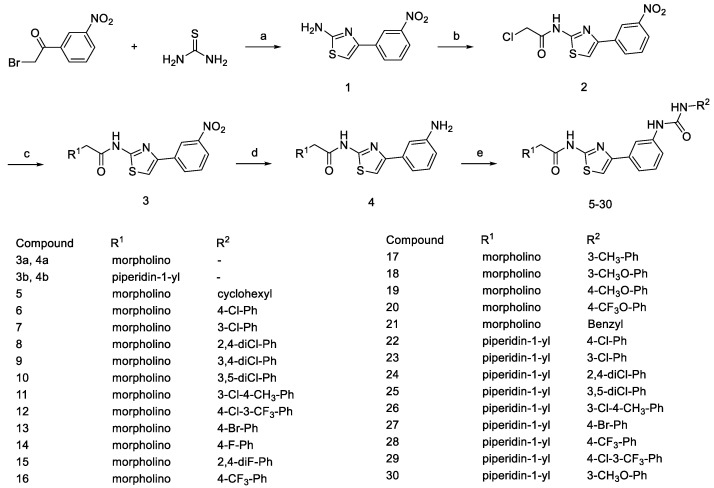
Synthesis of compounds **5**~**30**. Reagents and conditions: (a) 95% EtOH, reflux, 2 h; (b) 2-chloroacetyl chloride, CH_2_Cl_2_, K_2_CO_3_, r.t.; (c) secondary amines, K_2_CO_3_, KI, acetone, reflux, 12 h; (d) NaBH_4_, NiCl_2_·_6_H_2_O, CH_3_OH, 0 °C, 1 h; (e) isocyanate derivatives, CH_2_Cl_2_, reflux.

The general synthetic procedures for the target compounds **5**–**30** are outlined in Scheme 1. The commercially available starting material 2-bromo-1-(3-nitrophenyl)ethan-1-one was treated with thiourea in 95% ethanol to obtain 4-(3-nitrophenyl)thiazol-2-amine **1**. Amide **2** can be generated from the reaction of 4-(3-nitrophenyl)thiazol-2-amine **1** with 2-chloroacetyl chloride under basic conditions. The synthetic conditions were optimized in the presence of different basic reagents, such as sodium carbonate and potassium carbonate, etc. Inorganic weak bases produced a much higher yield (>90%) of Amide **2** compared with triethylamine (<60%). When using triethylamine as a binding acid agent and with the reactants at a scale above grams, the conversion of reactants in the later stages of the reaction is not complete, leading to an increased amount of impurities due to excess 2-chloroacetyl chloride and triethylamine. Amide **2** reacts with secondary amines like morpholine or piperidine, leading to alkylation at the chlorine atom, forming compound **3**. Then, compound **3** undergoes nitro reduction to produce compound **4**. In the final step, substituted aniline **4** and isocyanate derivatives react in dichloromethane solvent to yield the target compounds. Applying reflux conditions during the reaction can significantly reduce the reaction time, while having a minimal impact on the yield.

### 2.2. Biological Evaluation

#### 2.2.1. Preliminary Antiproliferative Activity

Compounds **5**–**30** were evaluated for their antiproliferative activity, using the standard CCK-8 assay against the human liver cancer cell lines HuH-7 and HepG2. As shown in Table 1, increasing the compound concentration resulted in higher inhibition rates being exhibited by most compounds across both the HuH-7 and HepG2 cell lines.

Notably, most of the screened compounds demonstrated comparable antiproliferative effects to the positive control drug, Sorafenib. Compounds **6**, **7**, **9**–**16**, and **18**–**21**, which contain morpholine moieties, displayed superior activity. In contrast, compounds **25**–**30**, containing piperidine fragments, also exhibited favorable activity. This suggests that the presence of the oxygen atoms in the morpholine ring of the side chain has a relatively small impact on inhibitory activity. When the R^2^ substituent is a phenyl group substituted with chlorine, bromine, or methoxy, the compounds generally exhibit better antiproliferative activity. However, the specific substitution positions on the phenyl ring did not reveal a consistent structure–activity relationship.

Moreover, the selected compounds were evaluated for their IC_50_ values against a broader spectrum of cancer cell lines, including human hepatoma cells (HepG2, QGY7703, SMMC-7721, Huh-7, PLC) and human breast cancer cells (MCF7). As shown in Table 2, compared to Sorafenib, compound **27** exhibited more potent antiproliferative activities against HepG2 cell lines and demonstrated considerable inhibitory effects on Huh-7, SMMC-7721, and QGY7703 cells. This implies that compounds **13**, **20**, **21**, **25**, **27**, and **28**, with a specific focus on compound **27**, emerge as more promising candidates for potential anticancer drug development, warranting further investigation.

#### 2.2.2. Compound **27** Inhibited the Growth of HepG2 Cells

**Figure 3:**
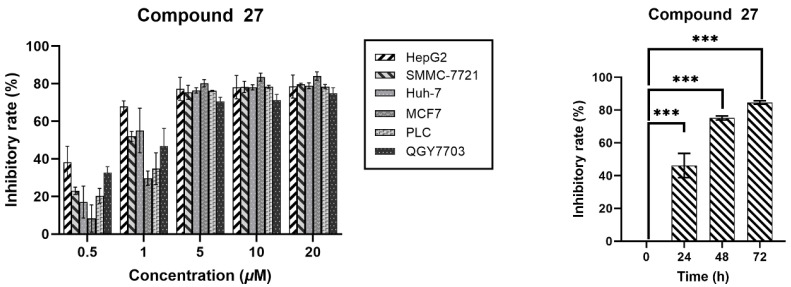
Inhibitory effect of compound **27** on HepG2 cells (n = 3; *** *p* < 0.001 vs. the DMSO group).

To comprehensively evaluate the anticancer efficacy of compound **27**, we exposed HepG2 hepatocellular carcinoma cells to a range of concentrations, with a 48 h incubation of this compound. Cell growth inhibition was quantified using the CCK-8 cell viability assay. As illustrated in Figure 3, compound **27** significantly suppressed the proliferation of HepG2 cells in a concentration-dependent manner. The inhibitory effect remained consistent as the concentration increased, plateauing when it surpassed 5.0 μM. Furthermore, at a constant concentration of 5.0 μM, the growth inhibition increased with prolonged incubation time with compound **27**. Collectively, these experimental findings demonstrate that compound **27** effectively restrains the growth of HepG2 cells within a specific range of concentrations and incubation durations, exhibiting a clear correlation with both time and concentration.

#### 2.2.3. Compound **27** Inhibited the Migration of HepG2 Cell

**Figure 4:**
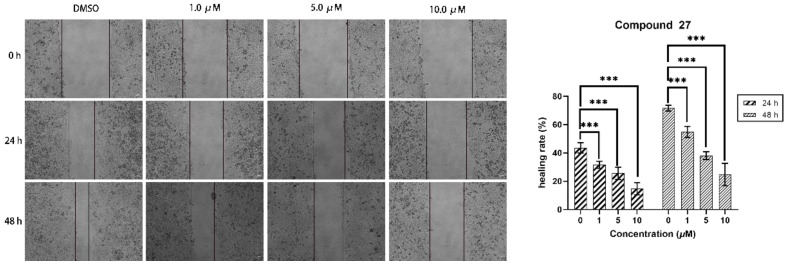
Compound **27** effectively inhibited the migration of HepG2 cells in the wound-healing assay (n = 3; *** *p* < 0.001 vs. the DMSO group).

The wound-healing assay is a commonly used method to study cell migration ability. The principle of this method is to artificially create a blank area, referred to as a “scratch”, on a confluent cell monolayer. The cells at the edge of the scratch will gradually migrate into the blank area and proliferate until the “scratch” is healed. This method is widely used to observe the effects of exogenous factors, such as drugs, on cell migration and repair. The changes in the distance of the scratch or the number of cells within the scratch are primarily used to reflect the rate of cell migration. In this study, we employed a wound-healing assay to investigate the influence of compound **27** on HepG2 cell migration. As depicted in Figure 4, the width of scratch healing at 48 h showed a clear dose-dependent decrease with increasing concentrations of compound **27**. Relative to the initial scratches, in the control group (0 μM), the scratches had almost completely healed at 48 h, boasting a healing rate of approximately 69.8%. As the concentration of compound **27** increased, the healing rate gradually diminished. Specifically, at a concentration of 1.0 μM/mL, the healing rate was 52.7% after 48 h, while at 5.0 μM/mL, it reduced to 38.4%, and further dropped to 33.5% at a 10.0 μM/mL concentration. The experimental results demonstrated that compound **27** significantly reduced HepG2 cell migration in a dose-dependent manner.

#### 2.2.4. Compound **27** Inhibits Colony Formation in HepG2 Cells

**Figure 5:**
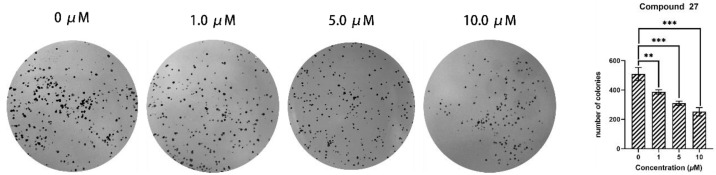
Compound **27** inhibits clonogenic formation of HepG2 cells (n = 3; ** *p* < 0.01, *** *p* < 0.001 vs. DMSO group).

The influence of compound **27** on the proliferative capacity of HepG2 cells was investigated through a clonogenic formation experiment, delineated in Figure 5. The results revealed that the cell colony count in the control group stood at 509.67 ± 36.08. At concentrations of 1.0, 5.0, and 10.0 μM, the corresponding cell colony counts were 387 ± 11.58, 310 ± 11.52, and 251.67 ± 23.23, respectively. In contrast to the control group, a conspicuous reduction in cell colony counts was observed at various concentrations, with a concomitant decline evident as the concentration of compound **27** escalated. These findings affirm the pronounced inhibitory impact of compound **27** on the proliferation capacity of HepG2 cells, illustrating a concentration-dependent modulation.

#### 2.2.5. Cell Cycle Analysis

**Figure 6:**
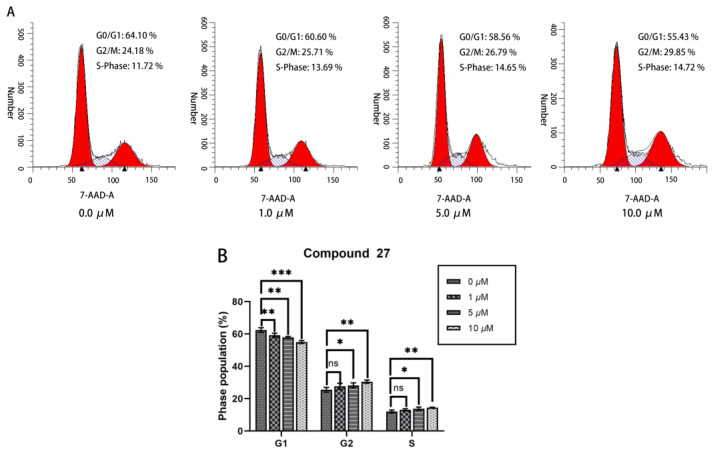
(**A**) Cell cycle analysis of HepG2 cells treated with compound **27**. (**B**) Quantification of HepG2 cells at various phases of the cell cycle. Each point represents the average of three independent experiments (n = 3; * *p* < 0.05, ** *p* < 0.01, *** *p* < 0.001 vs. DMSO group).

This investigation employed cell cycle analysis to elucidate the anti-tumor mechanism of compound **27** on HepG2 cells. As shown in Figure 6A,B, increasing the concentration of compound **27** was associated with a concurrent reduction in the proportion of G2/M-phase cells—from 62.45 ± 1.17% in the control group to 55.05 ± 0.76% at 10.0 μM. Simultaneously, escalating the concentration of compound **27** led to an augmentation in the proportion of G2-phase cells—increasing from 25.47 ± 1.23% in the control group to 30.51 ± 0.81% (10.0 μM). These findings suggest that compound **27** blocks the progression of HepG2 cells from the G2 phase to the M phase of the cell cycle, resulting in G2/M cell cycle arrest. This mechanism likely contributes to the anti-tumor activity of compound **27** against HepG2 cells.

#### 2.2.6. Apoptosis Analysis

In cell apoptosis analysis, the horizontal axis (FITC-A) represents the cell-staining indicator, which is typically Annexin V stained with the green fluorescent probe FITC. Annexin V can specifically bind to externalized phosphatidylserine on the cell membrane, and it is a common indicator for detecting early apoptotic cells. The vertical axis (7-AAD-A) represents another cell-staining indicator, which is typically propidium iodide (PI) staining. PI can penetrate the cell membrane and enter the cell nucleus, staining the DNA, and it is used to detect late apoptotic or necrotic cells.

**Figure 7:**
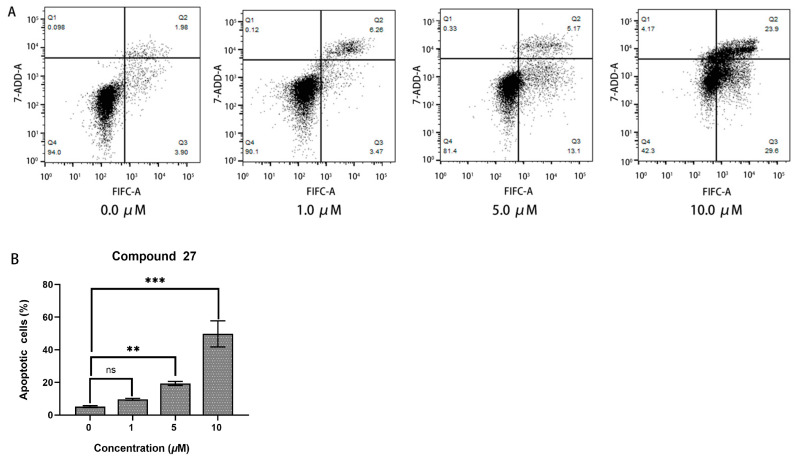
(**A**) The apoptosis analysis of HepG2 cells treated with compound **27**. (**B**) Comparison of the apoptosis response at different concentration of compound **27** (n = 3; ** *p* < 0.01, *** *p* < 0.001 vs. the DMSO group, ns means “not significant”).

The apoptotic response of HepG2 cells to compound **27** was evaluated using flow cytometry analysis (refer to Figure 7). The results reveal that the percentage of early apoptotic cells in the control group was 5.26 ± 0.29%. In contrast, treatment with 10.0 μM of compound **27** induced a significantly higher apoptotic rate of 49.77 ± 7.98%, particularly with a notable induction in early-stage apoptosis. These findings demonstrate that compound **27** effectively induced apoptosis in HepG2 cells in a concentration-dependent manner.

#### 2.2.7. Protein Kinase Inhibition Activity

The target compound in this study is designed based on the structures of Sorafenib, Sunitinib, and Nintedanib. The selected targets include FDA-approved ani-hepatocellular carcinoma drug targets within the Ras/MAPK signaling pathway and PI3K-AKT/PKB pathway, which are related to the aforementioned drugs. Additionally, our research group has long studied similar compounds and identified that the targets of active compounds with a structural similarity to the designed compound in this work include c-RAF, FLT3, c-MET, and EGFR [18,22,23,24]. Furthermore, this study incorporates clinical drug targets identified through GO and KEGG enrichment analyses, which are also FDA-approved targets (refer to the Supplemental Materials for details). The specific results are presented in Table 3; among these kinases, compound **27** demonstrates significant inhibitory activity against IGF1R, achieving a notable inhibition of 76.84%. Additionally, compound **27** also displays considerable inhibitory activity against EGFR, reaching 24.36%. Regarding other kinases, compound **27** exhibits inhibitory effects on VEGFR1 and PDGFRβ, with inhibitions of 11.86% and 11.72%, respectively. However, for some other kinases, such as c-KIT, Flt-3, and PDGFRα, the compound **27** shows a negative effect.

### 2.3. Molecular Docking Study

**Figure 8:**
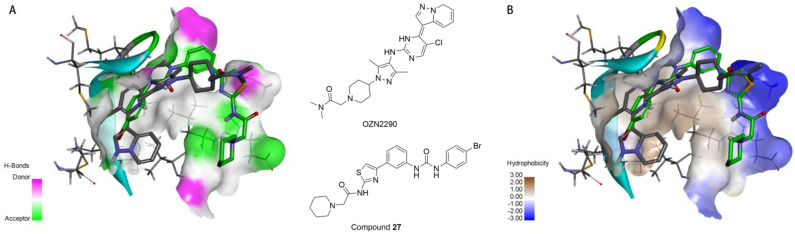
(**A**) The binding of compound **27** (C atoms in green) and the inhibitor OZN2290 (C atoms in gray) to the active site, with the surface colored by hydrogen bond types (hydrogen bond donors in magenta, hydrogen bond acceptors in green). (**B**) The binding of compound **27** (C atoms in green) and the inhibitor OZN2290 (C atoms in gray) to the active site, with the surface colored by hydrophobicity types (ranging from hydrophilic in blue to hydrophobic in brown).

Both compound **27** and the novel IGF1R inhibitor OZN2290 were found to effectively bind to the unphosphorylated active site of the IGF1R kinase domain (PDB code: 5FXS) [25]. As shown in Figure 8A, the 2-(piperidin-1-yl)acetamide fragment of compound **27** projects towards the solvent-exposed region outside the binding pocket. The tertiary amine nitrogen of the piperidine ring can potentially form favorable interactions with hydrogen bond acceptor residues in this region, especially when protonated. Replacing the piperidine with a morpholine ring, which also contains a similar tertiary amine, also could enhance the binding affinity between the receptor and the ligand.

Additionally, as depicted in Figure 8B, the region accommodating the 2-(piperidin-1-yl)acetamide fragment of compound **27** is lined with both hydrophobic and hydrophilic amino acid residues. The neutral or protonated piperidine/morpholine moieties can potentially achieve a high binding affinity in this area. The substituted phenyl group of compound **27** points towards the hinge region of the binding site, where neither prominent hydrophobic nor hydrogen bond donor residues are present. Considering the flexibility of compound **27**, this could help explain the relatively high potency observed for many of the target compounds.

**Figure 9:**
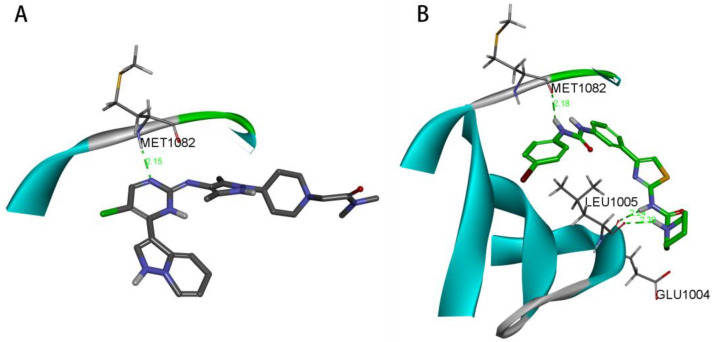
(**A**) Hydrogen bond interaction between OZN2290 and IGF1R. (**B**) Hydrogen bond interaction between compound **27** and IGF1R.

In Figure 9A, OZN2290 forms a classical hydrogen bond (2.10 Å) with the MET1082 residue of the IGF1R enzyme. In contrast, as shown in Figure 9B, compound **27** establishes three classical hydrogen bonds with the MET1082, GLU1004, and LEU1005 residues of the same enzyme, with hydrogen bond lengths of 2.18 Å, 2.24 Å, and 2.30 Å, respectively. Notably, compound **27** forms a greater number of hydrogen bond interactions with the IGF1R enzyme compared to OZN2290, signifying a stronger binding affinity of compound **27** for the target.

## 3. Materials and Methods

### 3.1. Chemistry

Unless otherwise stated, all materials were acquired from commercial suppliers and used without additional purification. ^1^H NMR and ^13^C NMR spectra were recorded using a 400-MR NMR Spectrometer (400 MHz for ^1^H and 101 MHz for ^13^C) (Agilent Technologies, Inc., Santa Clara, CA, USA). Chemical shifts are reported in parts per million (ppm) relative to TMS as internal standards. Column chromatography was conducted on silica gel (300~400 mesh). HRMS analyses were performed on an Agilent G6130A Quadrupole Mass Spectrometer with the Agilent 1200 Series (Agilent Technologies, Inc., Santa Clara, CA, USA).

#### 3.1.1. Synthesis of 4-(3-Nitrophenyl)thiazol-2-amine (**1**)

A mixture containing 2-bromo-1-(3-nitrophenyl)ethan-1-one (4.88 g, 20.0 mmol) and thiourea (1.60 g, 21.0 mmol) in 95% ethanol (50 mL) was heated at reflux for 2 h. The resulting solution was concentrated and subsequently combined with 100 mL of ice-cold water. The pH of the mixture was adjusted to 7 using saturated aqueous Na_2_CO_3_. The precipitate formed was then filtered and subsequently dried, yielding 4-(3-nitrophenyl)thiazol-2-amine.

Yellow solid; yield, 97.2%; m.p.: 269.2–270.8 °C; ^1^H NMR (400 MHz, Chloroform-*d*) δ 8.65 (dd, *J* = 4.1, 2.2 Hz, 1H), 8.13 (d, *J* = 10.6 Hz, 2H), 7.57 (td, *J* = 8.1, 4.0 Hz, 1H), 6.92 (d, *J* = 3.9 Hz, 1H), 5.07 (s, 2H); ^13^C NMR (101 MHz, Chloroform-*d*) δ 167.35, 148.88, 146.96, 140.81, 131.67, 129.50, 122.23, 120.86, 105.09; HRMS (*m/z*): calcd. for C_9_H_8_N_3_O_2_S [M + H]^+^ 222.0337, found 222.0338.

#### 3.1.2. Synthesis of 2-Chloro-N-(4-(3-nitrophenyl)thiazol-2-yl)acetamide (**2**)

To a solution of compound **1** (4.43 g, 20.0 mmol) in dichloromethane (20 mL) at 0 °C, sodium carbonate (2.12 g, 20.0 mmol) was added. The reaction mixture was stirred in an ice-water bath for 15 min. Subsequently, 2-chloroacetyl chloride (2.37 g, 20.0 mmol) in dichloromethane (20 mL) was added dropwise at room temperature to the stirring solution. The resulting mixture was stirred for 4 h at room temperature. After completion of the reaction, 20 mL of water was added to the reaction mixture and stirred for an additional 0.5 h. The mixture was then subjected to suction filtration, and the solid product was washed twice with water. The filtrate was separated into layers, and the organic solvent phase was dried and concentrated. Finally, the resulting solids were combined to obtain the desired product **2**.

Yellow solid; yield, 92.6%; m.p.: 218.1–219.9 °C; ^1^H NMR (400 MHz, Chloroform-*d*) δ 8.73 (s, 1H), 8.24–8.14 (m, 2H), 7.62 (t, *J* = 8.0 Hz, 1H), 7.39 (s, 1H), 4.35 (s, 2H); ^13^C NMR (101 MHz, Chloroform-*d*) δ 169.99, 161.91, 154.38, 148.70, 144.89, 131.66, 129.75, 122.74, 121.03, 110.38, 41.94; HRMS (*m/z*): calcd. for C_11_H_9_ClN_3_O_3_S [M + H]^+^ 298.0053, found 298.0051.

#### 3.1.3. Synthesis of Compounds **3**

In a 15 mL acetone solution, compound **2** (0.60 g, 2.0 mmol), secondary amines (2.5 mmol), potassium carbonate (0.33 g, 2.4 mmol), and potassium iodide (0.14 g, 0.80 mmol) were added and stirred for 5 min. The reaction mixture was subsequently refluxed for 12 h. After the reaction was completed, the mixture was diluted with water (50 mL), filtered, and then washed with water to obtain the desired compound **3**.

2-morpholino-*N*-(4-(3-nitrophenyl)thiazol-2-yl)acetamide (**3a**):

Yellow solid; yield, 89.6%; m.p.: 189.7–191.2 °C; ^1^H NMR (400 MHz, Chloroform-*d*) δ 10.40 (s, 1H), 8.76 (t, *J* = 2.0 Hz, 1H), 8.24–8.14 (m, 2H), 7.60 (t, *J* = 8.0 Hz, 1H), 7.34 (s, 1H), 3.88 (t, *J* = 4.7 Hz, 4H), 3.37–3.32 (m, 2H), 2.71 (t, *J* = 4.6 Hz, 4H); ^13^C NMR (101 MHz, Chloroform-*d*) δ 168.30, 157.36, 148.69, 147.57, 135.88, 131.62, 129.67, 126.56, 124.19, 122.57, 121.05, 109.92, 66.76, 61.38, 53.89; HRMS (*m/z*): calcd. For C_15_H_17_N_4_O_4_S [M + H]^+^ 349.0971, found 349.1002.

*N*-(4-(3-nitrophenyl)thiazol-2-yl)-2-(piperidin-1-yl)acetamide (**3b**):

Yellow solid; yield, 83.3%; m.p.: 220.5–221.8 °C; ^1^H NMR (400 MHz, Chloroform-*d*) δ 8.76 (d, *J* = 1.9 Hz, 1H), 8.18 (dt, *J* = 7.7, 1.6 Hz, 2H), 7.59 (td, *J* = 8.0, 1.4 Hz, 1H), 7.35–7.23 (m, 1H), 3.28 (s, 2H), 2.63 (s, 4H), 1.74 (q, *J* = 5.7 Hz, 4H), 1.54 (t, *J* = 6.7 Hz, 3H); ^13^C NMR (101 MHz, Chloroform-*d*) δ 169.28, 157.56, 148.67, 147.54, 136.00, 131.68, 129.62, 126.57, 124.15, 122.48, 121.03, 109.76, 61.62, 55.09, 25.90, 23.40; HRMS (*m*/*z*): calcd. for C_16_H_19_N_4_O_3_S [M + H]^+^ 347.1178, found 347.1240.

#### 3.1.4. Synthesis of Compounds **4**

Sodium borohydride (0.38 g, 10.0 mmol) was added to a mixture of compound **3** (2.0 mmol), NaBH_4_ (3.78 g, 100.0 mmol), and NiCl_2_·6H_2_O (1.19 g, 5.0 mmol) in 80% CH_3_OH (20 mL) in an ice-water bath. The reaction was stirred for 1 h and then concentrated. Then, 27% ammonium hydroxide (10 mL) was added, and the aqueous layer was extracted with dichloromethane (3 × 60 mL). The combined organic extracts were dried with Na_2_SO_4_, filtered, and concentrated to obtain the desired compound **4**.

*N*-(4-(3-aminophenyl)thiazol-2-yl)-2-morpholinoacetamide (**4a**):

Yellow solid; yield, 82.6%; m.p.: 182.3–184.7 °C; ^1^H NMR (400 MHz, DMSO-*d*_6_) δ 11.97 (s, 1H), 7.39 (s, 1H), 7.10 (s, 1H), 7.04 (d, *J* = 6.5 Hz, 2H), 6.52 (d, *J* = 6.9 Hz, 1H), 5.11 (s, 2H), 3.64–3.58 (m, 4H), 2.67–2.36 (m, 4H); ^13^C NMR (101 MHz, DMSO-*d*_6_) δ 168.88, 157.51, 150.17, 149.34, 135.30, 129.60, 114.07, 111.79, 107.71, 66.64, 60.86, 53.45; HRMS (*m*/*z*): calcd. For C_15_H_19_N_4_O_2_S [M + H]^+^ 319.1229, found 319.1231.

*N*-(4-(3-aminophenyl)thiazol-2-yl)-2-(piperidin-1-yl)acetamide (**4b**):

Yellow solid; yield, 84.6%; m.p.: 180.5–182.1 °C; ^1^H NMR (400 MHz, DMSO-*d*_6_) δ 11.76 (s, 1H), 7.52 (d, *J* = 8.5 Hz, 1H), 7.37 (s, 1H), 7.06 (t, *J* = 1.8 Hz, 1H), 7.00 (d, *J* = 2.1 Hz, 1H), 6.52 (m, 1H), 5.16 (m, 2H), 3.20 (d, *J* = 5.5 Hz, 2H), 2.44 (t, *J* = 5.1 Hz, 4H), 1.49 (t, *J* = 5.6 Hz, 4H), 1.35 (d, *J* = 5.8 Hz, 2H); ^13^C NMR (101 MHz, DMSO-*d*_6_) δ 169.25, 157.43, 150.07, 149.30, 135.21, 129.55, 127.13, 122.76, 113.97, 111.71, 107.65, 103.79, 61.40, 54.24, 25.98, 23.95; HRMS (*m*/*z*): calcd. for C_16_H_21_N_4_OS [M + H]^+^ 317.1436, found 317.1456.

#### 3.1.5. Synthesis of Compounds **5**~**30**

Compounds **4** (1.0 mmol) were added to a solution of isocyanate derivatives (1.1 mmol) in dichloromethane (15 mL). The mixture was stirred at reflux temperature and monitored using TLC. After completion of the reaction, the resulting solids were separated from the mixture through filtration and then washed with 10 mL of dichloromethane. The obtained residue was further purified using silica gel flash chromatography (DCM/MeOH = 40/1) to yield the desired compounds.

*N*-(4-(3-(3-cyclohexylureido)phenyl)thiazol-2-yl)-2-morpholinoacetamide (**5**): White solid; yield, 64.8%; m.p.: 206.8–208.0 °C; ^1^H NMR (400 MHz, dmso) δ 12.11 (s, 1H), 8.40 (s, 1H), 8.06 (s, 1H), 7.53 (s, 1H), 7.43 (d, J = 7.2 Hz, 1H), 7.25 (s, 4H), 6.13 (d, J = 7.7 Hz, 1H), 3.63 (s, 4H), 3.33 (s, 3H), 1.83 (d, J = 12.2 Hz, 2H), 1.69 (d, J = 15.1 Hz, 2H), 1.33 (d, J = 12.4 Hz, 2H), 1.19 (q, J = 11.6 Hz, 4H); ^13^C NMR (101 MHz, DMSO-*d*_6_) δ 168.90, 157.69, 154.80, 149.48, 141.34, 135.07, 129.39, 118.93, 117.45, 115.47, 108.40, 66.56, 60.78, 53.38, 48.10, 33.38, 25.67, 24.81; HRMS (*m*/*z*): calcd. ForC_22_H_30_N_5_O_3_S [M + H]^+^ 444.2069, found 444.2126.

*N*-(4-(3-(3-(4-chlorophenyl)ureido)phenyl)thiazol-2-yl)-2-morpholinoacetamide (**6**): White solid; yield, 73.5%; m.p.: 223.0–234.7 °C; ^1^H NMR (400 MHz, DMSO-*d*_6_) δ 12.13 (s, 1H), 8.91 (s, 1H), 8.84 (s, 1H), 8.17 (s, 1H), 7.61–7.49 (m, 4H), 7.40–7.27 (m, 4H), 3.63 (t, *J* = 4.5 Hz, 4H), 3.34 (s, 2H), 2.59–2.54 (m, 4H); ^13^C NMR (101 MHz, DMSO-*d*_6_) δ 168.95, 157.79, 152.85, 149.29, 140.36, 139.11, 135.24, 129.55, 129.06, 125.77, 120.15, 119.95, 118.25, 116.24, 108.67, 66.57, 60.80, 53.39; HRMS (*m*/*z*): calcd. For C_22_H_23_ClN_5_O_3_S [M + H]^+^ 472.1210, found 472.1232.

*N*-(4-(3-(3-(3-chlorophenyl)ureido)phenyl)thiazol-2-yl)-2-morpholinoacetamide (**7**): White solid; yield, 73.5%; m.p.: 211.0–212.3 °C; ^1^H NMR (400 MHz, DMSO-*d*_6_) δ 12.14 (s, 1H), 9.03 (d, *J* = 3.9 Hz, 1H), 8.93 (d, *J* = 3.9 Hz, 1H), 8.20 (s, 1H), 7.78 (s, 1H), 7.60 (d, *J* = 4.0 Hz, 1H), 7.54 (t, *J* = 5.6 Hz, 1H), 7.32 (q, *J* = 6.4 Hz, 4H), 7.05 (t, *J* = 5.4 Hz, 1H), 3.63 (d, *J* = 5.5 Hz, 4H), 3.34 (d, *J* = 4.0 Hz, 2H), 2.55 (d, *J* = 5.3 Hz, 4H); ^13^C NMR (101 MHz, DMSO-*d*_6_) δ 168.94, 157.79, 152.82, 149.26, 141.68, 140.26, 135.24, 133.62, 130.85, 129.56, 121.87, 120.02, 118.29, 117.93, 117.04, 116.27, 108.71, 66.56, 60.79, 53.38; HRMS (*m/z*): calcd. For C_22_H_23_ClN_5_O_3_S [M + H]^+^ 472.12101, found 472.125415.

*N*-(4-(3-(3-(2,4-dichlorophenyl)ureido)phenyl)thiazol-2-yl)-2-morpholin-4-ylacetamide (**8**): White solid; yield, 72.1%; m.p.: 219.6–220.8 °C; ^1^H NMR (400 MHz, DMSO-*d*_6_) δ 12.13 (s, 1H), 9.57 (s, 1H), 8.46 (s, 1H), 8.24 (dd, *J* = 14.5, 5.6 Hz, 2H), 7.66 (d, *J* = 2.8 Hz, 1H), 7.64–7.53 (m, 2H), 7.47–7.33 (m, 2H), 7.30 (d, *J* = 8.1 Hz, 1H), 3.67–3.59 (m, 4H), 3.34 (s, 2H), 2.55 (d, *J* = 5.0 Hz, 4H); ^13^C NMR (101 MHz, DMSO-*d*_6_) δ 168.96, 157.81, 152.39, 149.21, 140.08, 135.60, 135.33, 129.69, 129.01, 128.08, 126.54, 123.03, 122.49, 120.22, 118.13, 116.14, 108.77, 66.57, 60.79, 53.38; HRMS (*m*/*z*): calcd. For C_22_H_22_Cl_2_N_5_O_3_S [M + H]^+^ 506.0820, found 506.0892.

*N*-(4-(3-(3-(3,4-dichlorophenyl)ureido)phenyl)thiazol-2-yl)-2-morpholinoacetamide (**9**): White solid; yield, 76.6%; m.p.: 206.3.4–207.9 °C; ^1^H NMR (400 MHz, DMSO-*d*_6_) δ 12.15 (s, 1H), 9.57 (s, 1H), 9.33 (s, 1H), 8.18 (s, 1H), 7.95 (d, *J* = 2.4 Hz, 1H), 7.59 (s, 3H), 7.57–7.51 (m, 2H), 7.42–7.30 (m, 3H), 3.63 (t, *J* = 4.6 Hz, 4H), 3.34 (s, 2H), 2.58–2.54 (m, 4H); ^13^C NMR (101 MHz, DMSO-*d*_6_) δ 168.95, 157.82, 152.90, 149.28, 140.59, 140.29, 135.22, 131.42, 130.98, 129.52, 123.33, 120.04, 119.56, 118.67, 118.36, 116.31, 108.68, 66.57, 60.80, 53.38; HRMS (m/z): calcd. For C_22_H_22_Cl_2_N_5_O_3_S [M + H]^+^ 506.0820, found 506.0834.

*N*-(4-(3-(3-(3,5-dichlorophenyl)ureido)phenyl)thiazol-2-yl)-2-morpholinoacetamide (**10**): White solid; yield, 72.6%; m.p.: 222.4–223.9 °C; ^1^H NMR (400 MHz, DMSO-*d*_6_) δ 12.12 (s, 1H), 9.23 (s, 1H), 9.07 (s, 1H), 8.20 (s, 1H), 7.63–7.52 (m, 4H), 7.41–7.27 (m, 2H), 7.20 (t, *J* = 2.0 Hz, 1H), 3.63 (t, *J* = 4.6 Hz, 4H), 3.34 (s, 2H), 2.59–2.54 (m, 4H); ^13^C NMR (101 MHz, DMSO-*d*_6_) δ 168.94, 157.80, 152.68, 149.22, 142.71, 140.02, 135.26, 134.52, 129.56, 121.31, 120.26, 118.50, 116.71, 116.50, 108.74, 66.57, 60.81, 53.39; HRMS (*m*/*z*): calcd. For C_22_H_22_Cl_2_N_5_O_3_S [M + H]^+^ 506.0820, found 506.0850.

*N*-(4-(3-(3-(3-chloro-4-methylphenyl)ureido)phenyl)thiazol-2-yl)-2-morpholinoacetamide (**11**): White solid; yield, 74.1%; m.p.: 216.7–216.9 °C; ^1^H NMR (400 MHz, DMSO-*d*_6_) δ 12.12 (s, 1H), 8.83 (d, *J* = 4.8 Hz, 2H), 8.20 (d, *J* = 1.8 Hz, 1H), 7.76 (d, *J* = 2.1 Hz, 1H), 7.60 (s, 1H), 7.54 (d, *J* = 7.6 Hz, 1H), 7.40–7.17 (m, 4H), 3.63 (t, *J* = 4.5 Hz, 4H), 3.34 (s, 2H), 2.59–2.54 (m, 4H), 2.29 (s, 3H); ^13^C NMR (101 MHz, DMSO-*d*_6_) δ 168.94, 157.78, 152.85, 149.29, 140.34, 139.28, 135.23, 133.54, 131.61, 129.54, 128.69, 119.94, 118.53, 118.25, 117.38, 116.25, 109.99, 108.68, 66.57, 60.80, 53.38, 19.25; HRMS (*m/z*): calcd. For C_23_H_25_ClN_5_O_3_S [M + H]^+^ 486.1367, found 486.1421.

*N*-(4-(3-(3-(4-chloro-3-(trifluoromethyl)phenyl)ureido)phenyl)thiazol-2-yl)-2-morpholinoacetamide (**12**): White solid; yield, 75.7%; m.p.: 186.7–188.9 °C; ^1^H NMR (400 MHz, DMSO-*d*_6_) δ 12.14 (s, 1H), 9.21 (s, 1H), 8.95 (s, 1H), 8.22–8.14 (m, 2H), 7.65 (s, 2H), 7.60 (s, 1H), 7.55 (d, *J* = 7.6 Hz, 2H), 7.40–7.25 (m, 2H), 3.62 (t, *J* = 4.5 Hz, 4H), 3.33 (s, 2H), 2.54 (d, *J* = 4.5 Hz, 3H); ^13^C NMR (101 MHz, DMSO-*d*_6_) δ 164.35, 153.22, 148.22, 144.63, 135.46, 135.18, 130.68, 127.85, 124.98, 122.66, 122.36, 120.02, 118.90, 118.12, 118.11, 117.31, 115.66, 113.94, 112.62, 112.57, 112.50, 112.44, 111.88, 104.18, 61.98, 56.19, 48.79; HRMS (*m*/*z*): calcd. For C_23_H_22_ClF_3_N_5_O_3_S [M + H]^+^ 540.1084, found 540.1176.

*N*-(4-(3-(3-(4-bromophenyl)ureido)phenyl)thiazol-2-yl)-2-morpholinoacetamide (**13**): White solid; yield, 74.1%; m.p.: 227.8–228.9 °C; ^1^H NMR (400 MHz, DMSO-*d*_6_) δ 12.12 (s, 1H), 8.88 (s, 1H), 8.82 (s, 1H), 8.17 (s, 1H), 7.59 (s, 1H), 7.54 (d, *J* = 7.6 Hz, 1H), 7.48 (s, 4H), 7.40–7.26 (m, 2H), 3.63 (t, *J* = 4.5 Hz, 4H), 3.34 (s, 2H), 2.59–2.54 (m, 4H); ^13^C NMR (101 MHz, DMSO-*d*_6_) δ 168.95, 157.79, 152.81, 149.28, 140.32, 139.53, 135.24, 131.95, 129.56, 120.56, 119.97, 118.26, 116.26, 113.66, 108.68, 66.57, 60.80, 53.39; HRMS (m/z): calcd. For C_22_H_23_BrN_5_O_3_S [M + H]^+^ 516.0705, found 516.0745.

*N*-(4-(3-(3-(4-fluorophenyl)ureido)phenyl)thiazol-2-yl)-2-morpholinoacetamide (**14**): White solid; yield, 72.7%; m.p.: 214.8–215.9 °C; ^1^H NMR (400 MHz, DMSO-*d*_6_) δ 12.14 (s, 1H), 8.77 (d, *J* = 4.7 Hz, 2H), 8.16 (t, *J* = 1.9 Hz, 1H), 7.58 (s, 1H), 7.54–7.44 (m, 3H), 7.39–7.25 (m, 2H), 7.15 (t, *J* = 8.9 Hz, 2H), 3.62 (t, *J* = 4.5 Hz, 4H), 3.33 (s, 2H), 2.54 (d, *J* = 4.5 Hz, 4H); ^13^C NMR (101 MHz, DMSO-*d*_6_) δ 164.34, 154.34, 153.19, 151.97, 148.44, 144.72, 135.92, 131.84, 131.82, 130.63, 124.94, 115.83, 115.75, 115.21, 113.58, 111.57, 111.25, 111.03, 104.06, 61.98, 56.20, 48.79; HRMS (*m/z*): calcd. For C_22_H_23_FN_5_O_3_S [M + H]^+^ 456.1506, found 456.1577.

*N*-(4-(3-(3-(2,4-difluorophenyl)ureido)phenyl)thiazol-2-yl)-2-morpholinoacetamide (**15**): White solid; yield, 75.6%; m.p.: 217.0–218.8 °C; ^1^H NMR (400 MHz, DMSO-*d*_6_) δ 12.14 (s, 1H), 9.13 (s, 1H), 8.57 (d, *J* = 2.4 Hz, 1H), 8.17 (d, *J* = 1.8 Hz, 1H), 8.11 (td, *J* = 9.2, 6.1 Hz, 1H), 7.60 (s, 2H), 7.54 (d, *J* = 7.6 Hz, 1H), 7.41–7.26 (m, 3H), 7.09 (tt, *J* = 8.8, 2.3 Hz, 1H), 3.63 (t, *J* = 4.5 Hz, 4H), 3.34 (s, 2H), 2.59–2.54 (m, 3H); ^13^C NMR (101 MHz, DMSO-*d*_6_) δ 168.95, 158.49, 158.38, 157.80, 156.10, 155.98, 153.89, 153.77, 152.71, 151.46, 151.34, 149.23, 140.24, 135.29, 129.64, 124.52, 124.49, 124.42, 124.38, 122.47, 122.44, 122.38, 122.35, 120.04, 118.06, 116.03, 111.62, 111.58, 111.40, 111.37, 108.73, 104.49, 104.25, 104.22, 103.98, 66.57, 60.79, 53.38; HRMS (*m/z*): calcd. For C_22_H_22_F_2_N_5_O_3_S [M + H]^+^ 474.1411, found 474.1512.

2-morpholino-*N*-(4-(3-(3-(4-(trifluoromethyl)phenyl)ureido)phenyl)thiazol-2-yl)acetamide (**16**): White solid; yield, 75.8%; m.p.: 220.4–221.2 °C; ^1^H NMR (400 MHz, DMSO-*d*_6_) δ 12.15 (s, 1H), 9.21 (s, 1H), 8.95 (s, 1H), 8.19 (s, 1H), 7.74–7.63 (m, 4H), 7.60 (s, 1H), 7.56 (d, *J* = 7.5 Hz, 1H), 7.35 (dt, *J* = 16.7, 8.2 Hz, 2H), 3.63 (t, *J* = 4.5 Hz, 4H), 3.34 (s, 2H), 2.57–2.54 (m, 4H); ^13^C NMR (101 MHz, DMSO-*d*_6_) δ 168.97, 157.82, 152.71, 149.23, 143.87, 140.13, 135.26, 129.60, 126.60, 126.56, 126.53, 126.49, 126.33, 123.64, 122.32, 122.00, 120.17, 118.39, 118.25, 116.34, 108.75, 66.56, 60.78, 53.38; HRMS (*m/z*): calcd. For C_23_H_23_F_3_N_5_O_3_S [M + H]^+^ 506.1474, found 506.15334.

2-morpholino-*N*-(4-(3-(3-(m-tolyl)ureido)phenyl)thiazol-2-yl)acetamide (**17**): White solid; yield, 77.2%; m.p.: 197.5–198.0 °C; ^1^H NMR (400 MHz, DMSO-*d*_6_) δ 12.15 (s, 1H), 8.77 (s, 1H), 8.66 (s, 1H), 8.19 (d, *J* = 1.8 Hz, 1H), 7.59 (s, 1H), 7.52 (d, *J* = 7.6 Hz, 1H), 7.39–7.15 (m, 5H), 6.82 (d, *J* = 7.3 Hz, 1H), 3.63 (t, *J* = 4.6 Hz, 4H), 3.34 (s, 2H), 2.58–2.54 (m, 4H), 2.31 (s, 3H); ^13^C NMR (101 MHz, DMSO-*d*_6_) δ 168.94, 157.78, 152.91, 149.33, 140.55, 139.99, 138.40, 135.22, 129.54, 129.08, 123.03, 119.75, 119.10, 118.07, 116.06, 115.78, 108.65, 66.56, 60.80, 53.38, 21.67; HRMS (*m/z*): calcd. For C_23_H_26_N_5_O_3_S [M + H]^+^ 452.1756, found 452.1830.

*N*-(4-(3-(3-(3-methoxyphenyl)ureido)phenyl)thiazol-2-yl)-2-morpholinoacetamide (**18**): White solid; yield, 73.0%; m.p.: 195.0–196.9 °C; ^1^H NMR (400 MHz, DMSO-*d*_6_) δ 12.15 (s, 1H), 8.77 (d, *J* = 7.2 Hz, 2H), 8.16 (t, *J* = 1.9 Hz, 1H), 7.59 (s, 1H), 7.55–7.48 (m, 1H), 7.38–7.25 (m, 2H), 7.25–7.14 (m, 2H), 6.95 (dd, *J* = 7.9, 1.9 Hz, 1H), 6.57 (dd, *J* = 8.3, 2.5 Hz, 1H), 3.75 (s, 3H), 3.62 (t, *J* = 4.6 Hz, 5H), 3.33 (s, 2H), 2.54 (d, *J* = 4.5 Hz, 3H); ^13^C NMR (101 MHz, DMSO-*d*_6_) δ 164.34, 155.51, 153.20, 148.27, 144.72, 136.73, 135.88, 130.64, 125.41, 124.95, 115.24, 113.57, 111.53, 106.31, 104.08, 103.07, 99.70, 61.98, 56.19, 50.76, 48.79; HRMS (*m*/*z*): calcd. For C_23_H_26_N_5_O_4_S [M + H]^+^ 468.1706, found 468.1736.

*N*-(4-(3-(3-(4-methoxyphenyl)ureido)phenyl)thiazol-2-yl)-2-morpholinoacetamide (**19**): White solid; yield, 74.5%; m.p.: 193.6–195.8 °C; ^1^H NMR (400 MHz, DMSO-*d*_6_) δ 12.07 (s, 1H), 8.62 (s, 1H), 8.46 (s, 1H), 8.10 (s, 1H), 7.52 (s, 1H), 7.45 (dt, *J* = 7.5, 1.5 Hz, 1H), 7.38–7.29 (m, 2H), 7.32–7.19 (m, 2H), 6.89–6.80 (m, 2H), 3.69 (s, 3H), 3.58 (t, *J* = 4.6 Hz, 4H), 3.28 (s, 2H), 2.49 (t, *J* = 4.6 Hz, 4H); ^13^C NMR (101 MHz, DMSO-*d*_6_) δ 168.93, 157.77, 154.90, 153.12, 149.38, 140.72, 135.20, 133.08, 129.50, 120.44, 119.61, 118.03, 116.04, 114.41, 108.60, 66.58, 60.80, 55.60, 53.39; HRMS (*m/z*): calcd. For C_23_H_26_N_5_O_4_S [M + H]^+^ 468.1706, found 468.1732.

2-morpholino-*N*-(4-(3-(3-(4-(trifluoromethoxy)phenyl)ureido)phenyl)thiazol-2-yl)acetamide (**20**): White solid; yield, 72.7%; m.p.: 213.4–214.9 °C; ^1^H NMR (400 MHz, DMSO-*d*_6_) δ 12.07 (s, 1H), 8.89 (s, 1H), 8.78 (s, 1H), 8.12 (s, 1H), 7.59–7.51 (m, 3H), 7.48 (d, *J* = 7.4 Hz, 1H), 7.35–7.22 (m, 4H), 3.58 (t, *J* = 4.6 Hz, 4H), 3.28 (s, 2H), 2.50 (t, *J* = 4.6 Hz, 4H); ^13^C NMR (101 MHz, DMSO-*d*_6_) δ 168.94, 157.80, 152.88, 149.29, 143.01, 142.99, 140.34, 139.42, 135.26, 129.55, 122.18, 121.89, 119.99, 119.80, 119.35, 118.28, 116.26, 108.69, 66.58, 60.80, 53.39; HRMS (*m/z*): calcd. For C_23_H_23_F_3_N_5_O_4_S [M + H]^+^ 522.1423, found 522.14583.

*N*-(4-(3-(3-benzylureido)phenyl)thiazol-2-yl)-2-morpholinoacetamide (**21**): White solid; yield, 88.7%; m.p.: 191.0–192.9 °C; ^1^H NMR (400 MHz, DMSO-*d*_6_) δ 12.14 (s, 1H), 8.67 (s, 1H), 8.11 (s, 1H), 7.54 (s, 1H), 7.44 (dt, *J* = 7.0, 1.9 Hz, 1H), 7.40–7.22 (m, 8H), 6.68 (t, *J* = 6.0 Hz, 1H), 4.32 (d, *J* = 5.8 Hz, 2H), 3.61 (t, *J* = 4.6 Hz, 4H), 3.32 (s, 2H), 2.54 (d, *J* = 4.2 Hz, 3H); ^13^C NMR (101 MHz, DMSO-*d*_6_) δ 164.31, 153.13, 151.00, 144.88, 136.67, 136.25, 130.51, 124.80, 124.15, 122.93, 122.55, 114.52, 113.04, 111.11, 103.85, 61.97, 56.18, 48.78, 38.59; HRMS (*m/z*): calcd. For C_23_H_26_N_5_O_3_S [M + H]^+^ 452.1756, found 452.1780.

*N*-(4-(3-(3-(4-chlorophenyl)ureido)phenyl)thiazol-2-yl)-2-(piperidin-1-yl)acetamide (**22**): White solid; yield, 79.6%; m.p.205.4–206.2 °C; ^1^H NMR (400 MHz, DMSO-*d*_6_) δ 11.92 (s, 1H),8.88–8.76 (m, 2H), 7.78 (d, *J* = 8.7 Hz, 1H), 7.56–7.43 (m, 5H), 7.34–7.23 (m, 3H), 3.23 (d, *J* = 3.5 Hz, 2H), 2.45 (d, *J* = 7.0 Hz, 4H), 1.49 (t, *J* = 5.6 Hz, 4H), 1.35 (s, 2H); ^13^C NMR (101 MHz, DMSO-*d*_6_) δ 169.27, 157.73, 152.77, 149.17, 140.37, 139.64, 139.09, 135.24, 129.08, 128.61, 126.71, 125.79, 120.15, 118.70, 106.83, 61.38, 54.23, 25.94, 23.94; HRMS (*m*/*z*): calcd. For C_23_H_25_ClN_5_O_2_S [M + H]^+^ 470.1418, found 470.1495.

*N*-(4-(3-(3-(3-chlorophenyl)ureido)phenyl)thiazol-2-yl)-2-(piperidin-1-yl)acetamide (**23**): White solid; yield, 73.6%; m.p.: 201.5–202.6 °C; ^1^H NMR (400 MHz, DMSO-*d*_6_) δ 11.77 (s, 1H),8.95 (s, 1H), 8.88 (s, 1H), 7.82–7.76 (m, 2H), 7.70 (t, *J* = 2.0 Hz, 1H), 7.52–7.45 (m, 3H), 7.29–7.23 (m, 2H), 6.99 (dt, *J* = 7.4, 1.9 Hz, 1H), 3.24 (s, 2H), 2.46 (d, *J* = 1.9 Hz, 2H), 1.50 (p, *J* = 5.5 Hz, 4H), 1.36 (dd, *J* = 9.0, 4.6 Hz, 2H); ^13^C NMR (101 MHz, DMSO-*d*_6_) δ 169.18, 157.72, 152.73, 149.16, 141.65, 139.52, 133.63, 130.86, 128.71, 126.71, 121.91, 118.78, 117.96, 117.07, 106.89, 63.48, 61.30, 54.22, 25.87, 23.89; HRMS (*m*/*z*): calcd. For C_23_H_25_ClN_5_O_2_S [M + H]^+^ 470.1418, found 470.1493.

*N*-(4-(3-(3-(2,4-dichlorophenyl)ureido)phenyl)thiazol-2-yl)-2-(piperidin-1-yl)acetamide (**24**): Yellow solid; yield, 75.2%; m.p.: 213.9–215.6 °C; ^1^H NMR (400 MHz, DMSO-*d*_6_) δ 11.91 (s, 1H), 9.52 (s, 1H), 8.40 (s, 1H), 8.19 (d, J = 8.9 Hz, 1H), 8.14 (t, J = 1.9 Hz, 1H), 7.61 (d, J = 2.4 Hz, 1H), 7.55 (s, 1H), 7.50 (d, J = 7.5 Hz, 1H), 7.37 (dd, J = 9.0, 2.5 Hz, 1H), 7.32 (t, J = 7.8 Hz, 1H), 7.28–7.22 (m, 1H), 3.23 (s, 2H), 2.45 (t, J = 5.0 Hz, 4H), 1.50 (p, J = 5.5 Hz, 4H), 1.35 (s, 2H); ^13^C NMR (101 MHz, DMSO-*d*_6_) δ 169.35, 157.81, 152.40, 149.19, 140.11, 135.63, 135.34, 129.70, 129.02, 128.11, 126.52, 123.01, 122.47, 120.21, 118.11, 116.13, 108.76, 61.39, 54.23, 25.95, 23.93; HRMS (*m*/*z*): calcd. For C_23_H_24_Cl_2_N_5_O_2_S [M + H]^+^ 504.1028, found 504.1080.

*N*-(4-(3-(3-(3,5-dichlorophenyl)ureido)phenyl)thiazol-2-yl)-2-(piperidin-1-yl)acetamide (**25**): White solid; yield, 71.9%; m.p.: 201.7–203.0 °C; ^1^H NMR (400 MHz, DMSO-*d*_6_) δ 9.07 (d, *J* = 7.6 Hz, 1H), 8.97 (d, *J* = 11.0 Hz, 1H), 8.14 (t, *J* = 2.0 Hz, 1H), 7.83–7.76 (m, 1H), 7.56–7.46 (m, 4H), 7.35–7.21 (m, 1H), 7.14 (q, *J* = 1.7 Hz, 1H), 3.22 (d, *J* = 3.6 Hz, 2H), 2.43 (d, *J* = 5.6 Hz, 4H), 1.49 (p, *J* = 5.5 Hz, 4H), 1.35 (d, *J* = 5.3 Hz, 2H); ^1 13^C NMR (101 MHz, DMSO-*d*_6_) δ 169.38, 157.81, 152.67, 149.19, 142.70, 142.66, 140.02, 139.25, 135.26, 134.52, 129.58, 128.96, 126.70, 121.35, 120.27, 119.02, 118.51, 116.75, 116.50, 108.73, 106.99, 61.42, 54.23, 25.95, 23.95; HRMS (*m*/*z*): calcd. For C_23_H_24_Cl_2_N_5_O_2_S [M + H]^+^ 504.1028, found 504.1094.

*N*-(4-(3-(3-(3-chloro-4-methylphenyl)ureido)phenyl)thiazol-2-yl)-2-(piperidin-1-yl)acetamide (**26**): Yellow solid; yield, 73.8%; m.p.: 181.9–183.1 °C; ^1^H NMR (400 MHz, DMSO-*d*_6_) δ 11.84 (s, 1H), 8.79 (d, *J* = 4.7 Hz, 2H), 7.86–7.78 (m, 2H), 7.71 (d, *J* = 2.1 Hz, 1H), 7.55–7.46 (m, 3H), 7.28–7.16 (m, 2H), 3.25 (s, 2H), 2.52–2.45 (m, 4H), 2.27 (s, 3H), 1.54 (dq, *J* = 11.1, 5.2 Hz, 4H), 1.39 (tt, *J* = 8.5, 4.1 Hz, 2H); ^13^C NMR (101 MHz, DMSO-*d*_6_) δ 169.34, 157.77, 152.82, 149.26, 139.67, 139.31, 133.60, 131.64, 128.77, 128.72, 126.75, 118.80, 118.66, 117.48, 106.84, 61.49, 54.31, 26.02, 24.00, 19.28; HRMS (*m/z*): calcd. For C_24_H_27_ClN_5_O_2_S [M + H]^+^ 484.1574, found 484.1566.

*N*-(4-(3-(3-(4-bromophenyl)ureido)phenyl)thiazol-2-yl)-2-(piperidin-1-yl)acetamide (**27**): White solid; yield, 86.2%; m.p.: 210.2–212.0 °C; ^1^H NMR (400 MHz, DMSO-*d*_6_) δ 11.85 (s, 1H), 8.87–8.75 (m, 2H), 7.86–7.78 (m, 2H), 7.58–7.41 (m, 4H), 7.45 (s, 3H), 3.26 (d, *J* = 3.6 Hz, 2H), 2.48 (dd, *J* = 7.6, 2.7 Hz, 4H), 1.54 (p, *J* = 5.5 Hz, 4H), 1.39 (q, *J* = 4.9 Hz, 2H); ^13^C NMR (101 MHz, DMSO-*d*_6_) δ 169.34, 157.77, 152.78, 149.26, 140.39, 139.57, 135.32, 131.99, 129.57, 128.74, 126.76, 120.65, 120.04, 118.79, 118.33, 116.36, 113.71, 108.67, 106.86, 61.48, 54.31, 26.02, 24.00.; HRMS (*m*/*z*): calcd. For C_23_H_25_BrN_5_O_2_S [M + H]^+^ 514.0912, found 514.0905, 516.0885.

2-(piperidin-1-yl)-*N*-(4-(3-(3-(4-(trifluoromethyl)phenyl)ureido)phenyl)thiazol-2-yl)acetamide (**28**): White solid; yield, 70.8%; m.p.: 196.6–198.2 °C; ^1^H NMR (400 MHz, DMSO-*d*_6_) δ 10.98 (s, 1H), 9.20 (s, 1H), 8.97 (s, 1H), 7.85 (d, *J* = 8.5 Hz, 1H), 7.68 (d, *J* = 7.1 Hz, 5H), 7.56 (q, *J* = 10.3, 7.3 Hz, 3H), 3.28 (d, *J* = 4.2 Hz, 2H), 2.53 (s, 4H), 1.55 (t, *J* = 5.6 Hz, 4H), 1.40 (d, *J* = 7.4 Hz, 2H); ^13^C NMR (101 MHz, DMSO-*d*_6_) δ 169.18, 157.72, 152.73, 149.16, 141.65, 139.52, 133.63, 130.86, 128.71, 126.71, 121.91, 118.78, 117.96, 117.07, 106.89, 63.48, 61.30, 54.22, 25.87, 23.89; HRMS (*m*/*z*): calcd. For C_24_H_25_F_3_N_5_O_2_S [M + H]^+^ 504.1681, found 504.1693.

*N*-(4-(3-(3-(4-chloro-3-(trifluoromethyl)phenyl)ureido)phenyl)thiazol-2-yl)-2-(piperidin-1-yl)acetamide (**29**): White solid; yield, 84.2%; m.p. 201.3–203.0. °C; ^1^H NMR (400 MHz, DMSO-*d*_6_) δ 11.85 (s, 1H), 9.19 (s, 1H), 8.94 (s, 1H), 8.14 (d, *J* = 2.3 Hz, 1H), 7.87–7.79 (m, 2H), 7.69–7.58 (m, 2H), 7.57–7.47 (m, 3H), 3.25 (s, 2H), 2.56–2.41 (m, 4H), 1.53 (q, *J* = 5.3 Hz, 4H), 1.39 (p, *J* = 6.2 Hz, 2H); ^13^C NMR (101 MHz, DMSO-*d*_6_) δ 169.35, 157.80, 152.79, 149.19, 139.80, 139.34, 132.47, 129.02, 127.35, 127.05, 126.75, 124.66, 123.55, 122.80, 121.95, 119.09, 117.23, 107.00, 61.49, 54.30, 26.01, 24.00.; HRMS (*m*/*z*): calcd. For C_24_H_24_ClF_3_N_5_O_2_S [M + H]^+^ 538.12913, found 538.1284.

*N*-(4-(3-(3-(3-methoxyphenyl)ureido)phenyl)thiazol-2-yl)-2-(piperidin-1-yl)acetamide (**30**): Yellow solid; yield, 84.3%; m.p.: 176.9–177.6 °C; ^1^H NMR (400 MHz, DMSO-d6) δ 11.94 (s, 1H), 8.70 (d, *J* = 10.6 Hz, 2H), 8.10 (d, *J* = 2.0 Hz, 1H), 7.54 (s, 1H), 7.47 (dt, *J* = 7.5, 1.7 Hz, 1H), 7.33–7.19 (m, 2H), 7.22–7.09 (m, 2H), 6.91 (dd, *J* = 7.9, 2.0 Hz, 1H), 6.52 (dd, *J* = 8.2, 2.5 Hz, 1H), 3.70 (s, 3H), 3.23 (s, 2H), 2.45 (d, *J* = 5.3 Hz, 4H), 1.49 (p, *J* = 5.5 Hz, 4H), 1.35 (q, *J* = 6.7 Hz, 2H); ^13^C NMR (101 MHz, DMSO-*d*_6_) δ 169.35, 160.12, 157.80, 152.87, 149.30, 141.33, 140.48, 135.24, 130.01, 129.56, 119.85, 118.17, 116.13, 110.91, 108.66, 107.68, 104.30, 61.39, 55.36, 55.34, 54.23, 25.95, 23.95; HRMS (*m*/*z*): calcd. For C_24_H_28_N_5_O_3_S [M + H]^+^ 466.1913, found 466.1953.

### 3.2. Biology

#### 3.2.1. In Vitro Cytotoxic Activity

The assessment of the cytotoxicity of the investigational compounds was conducted across a spectrum of cancer cell lines, namely HepG2, QGY7703, SMMC-7721, Huh-7, PLC, and MCF-7, utilizing the standard CCK8 assay. The HepG2, Huh-7, PLC and MCF7 cell lines were provided by Wuhan Pricella Biotechnology Co., Ltd. (Wuhan, China), while the SMMC-7721 and QGY7703 cell lines were provided by Shanghai Fuheng Biotechnology Co., Ltd. (Shanghai, China). Cells were cultured in triplicate within a 96-well plate, with an initial seeding of 5 × 10^3^ cells in 1.0 mL, followed by a 24 h incubation period at 37 °C to ensure adhesion. The test compounds were introduced at the specified concentrations and allowed to incubate for 48 h at 37 °C. Subsequently, CCK8 reagent was administered, and the absorbance was measured at 450 nm following a 1 h incubation. IC_50_ values were determined using GraphPad 9.0 software.

#### 3.2.2. Colony-Forming Assay

HepG2 cells were seeded in triplicate in 6-well plates at a density of 800 cells per well during the logarithmic growth phase. Following an overnight incubation at 37 °C to promote cell attachment, the cells were treated either with the test compound or DMSO as a control. The incubation continued for a duration of 14 days. After the incubation period, the cells were fixed, stained, and photographed. The quantification of colony areas was performed using ImageJ Version 1.54i software.

#### 3.2.3. Scratch Migration Assay

Cells in the logarithmic growth phase were seeded in triplicate at a density of 5 × 10^5^ cells per well and incubated overnight at 37 °C. A straight scratch was intentionally generated to create a cell-free area. Subsequently, following a PBS-washing step, the cells were treated with varying concentrations of compound **27**. Images were captured at 0, 24, and 48 h using an inverted microscope. The analysis of these images was performed using ImageJ software.

#### 3.2.4. Cell Cycle Experiment

HepG2 cells (2 × 10^5^ cells/well) in logarithmic growth were treated with test compounds (0.0, 1.0, 5.0, 10.0 μM) for 48 h. Following trypsinization, fixation, and staining with 7-AAD, flow cytometry and ModFit LT 3.0 software were employed for analysis.

#### 3.2.5. Apoptosis Experiment

Logarithmically growing HepG2 cells (2 × 10^5^ cells/well) were exposed to test compounds (0.0, 1.0, 5.0, 10.0 μM) for 48 h. After trypsin digestion, Annexin V FITC and 7-AAD staining were performed, followed by flow cytometry analysis using FlowJo v10.10 software.

#### 3.2.6. Kinase Assay

A 10.0 μM concentration of compound **27** was tested in DMSO using a single-dose repeat mode. The experiment comprised two parts, utilizing assay buffers for both the HTRF and ADP-Glo kinase assays. For the HTRF assay, 50 nL of compound was transferred to a 384-well plate using Echo 655. After a 10 min incubation with kinase/metal solution, substrate, and ATP, fluorescence signals were recorded. In the ADP-Glo assay, 40 nL of compound was similarly transferred, and luminescence signals were recorded after incubation with kinase/metal solution, substrate, and ATP. Inhibition percentages for each test solution were calculated, setting the reading value of the reaction control (1% DMSO) as 0% inhibition and the background (10 µM positive control) as 100% inhibition.

### 3.3. Molecular Docking

The molecular docking investigation was conducted using the Discovery Studio 2017 software package developed by Accelrys. The generation of molecular conformations and tautomeric forms was performed using the Prepare Ligands module with default parameter settings. Crystal protein structures relevant to the study were retrieved from the Protein Data Bank (PDB) at https://www.rcsb.org/ (Accessed on 3 April 2024). These structures underwent preprocessing steps, including the removal of crystalline water and hydrogenation, followed by optimization through minimization operations. The binding site of the protein–ligand complex was specifically designated as the active site. Molecular docking simulations were carried out using the Dock Ligands (CDOCKER) module, with the active site serving as the central focus for the analysis.

## 4. Conclusions

Guided by the structural features of the multi-kinase inhibitors Sorafenib, Sunitinib, and Nintedanib, we incorporated morpholine/piperidine moieties into the 4-phenylthiazole scaffold to improve physicochemical properties and binding interactions. Biological evaluation revealed that many of the synthesized compounds exhibited a potent antiproliferative activity comparable to or exceeding Sorafenib against various liver cancer cell lines. Notably, compound **27** emerged as the most promising candidate, potently inhibiting HepG2’s cell proliferation, migration, and colony formation abilities. Mechanistic studies demonstrated that compound **27** induced G2/M cell cycle arrest and apoptosis in HepG2 cells. Kinase profiling identified IGF1R as a key target of compound **27**, which exhibited significant inhibition (76.84%) against this kinase. Molecular docking studies further corroborated the strong binding interactions of compound **27** with the IGF1R kinase domain via multiple hydrogen bonds. Collectively, these findings highlight compound **27** as a promising lead molecule for hepatocellular carcinoma therapeutics.

## Figures and Tables

**Table 1:** Antiproliferative activity of the target compounds (growth inhibition, %) ^a^.

Compound	HuH-7		HepG2	
	1 μM	5 μM	1 μM	5 μM
**5**	25.38	64.17	43.43	69.48
**6**	50.38	63.72	58.06	71.66
**7**	29.30	74.46	39.74	74.99
**8**	26.22	61.54	39.59	69.42
**9**	35.88	70.02	34.71	70.79
**10**	62.24	62.24	53.24	71.43
**11**	74.68	73.75	70.23	72.28
**12**	75.10	75.49	75.25	74.38
**13**	73.42	74.03	72.06	71.89
**14**	74.03	75.33	71.43	70.68
**15**	24.83	74.20	76.47	76.60
**16**	48.34	73.38	70.05	70.68
**17**	32.88	69.88	42.80	69.91
**18**	42.40	80.07	38.13	74.47
**19**	10.52	67.52	11.78	73.56
**20**	61.94	62.24	50.42	77.30
**21**	61.92	62.31	72.69	71.37
**22**	35.60	69.18	42.26	70.40
**23**	38.22	70.21	38.93	70.00
**24**	42.70	59.22	31.94	70.57
**25**	40.37	80.80	39.26	74.47
**26**	24.59	71.62	34.91	71.96
**27**	39.53	70.84	39.47	71.13
**28**	39.25	70.63	41.13	70.25
**29**	30.82	68.65	37.65	70.89
**30**	36.51	74.68	36.35	72.78
Sorafenib	21.28	78.48	31.45	71.01

^a^. the values are averaged from at least three independent experiments; variation ± 10%.

**Table 2:** Antiproliferative activities of selected compounds (IC_50_, μM) ^a^.

Compound	HepG2	QGY7703	SMMC-7721	Huh-7	PLC	MCF7
**13**	1.41 ± 0.43	3.44 ± 0.37	1.91 ± 0.22	1.68 ± 0.37	2.05 ± 0.26	2.32 ± 0.78
**20**	1.68 ± 0.48	1.73 ± 0.58	2.81 ± 0.49	1.37 ± 0.29	2.31 ± 0.38	0.98 ± 0.70
**21**	1.73 ± 0.70	1.01 ± 0.16	1.20 ± 0.07	1.78 ± 1.04	2.24 ± 0.77	1.54 ± 0.13
**25**	1.56 ± 0.55	2.39 ± 0.42	0.90 ± 0.32	1.96 ± 0.83	2.04 ± 0.69	1.88 ± 0.50
**27**	0.62 ± 0.34	1.54 ± 0.49	1.44 ± 0.23	1.52 ± 0.57	2.05 ± 0.16	2.17 ± 0.27
**28**	1.62 ± 0.76	2.58 ± 0.34	2.35 ± 0.77	2.45 ± 0.35	3.55 ± 0.54	2.32 ± 0.22
Sorafenib	1.62 ± 0.27	1.08 ± 0.21	1.31 ± 0.12	1.56 ± 0.22	1.01 ± 0.30	0.47 ± 0.12

^a^. Values are means ±SD of three independent experiments, each performed in triplicate.

**Table 3:** Kinase profile of compound **27**.

Protein	% Inhibition at 10 μM		
	n = 1	n = 2	Average
VEGFR1	10.23	13.50	11.86
VEGFR2	−7.34	6.75	−0.30
VEGFR3	−1.73	−7.59	−4.66
c-KIT	−27.23	−16.31	−21.77
B-Raf	−1.72	−0.81	−1.27
FLT3	−9.89	−18.78	−14.34
Raf-1	6.84	5.43	6.14
PDGFRα	−9.22	−7.77	−8.50
PDGFRβ	15.95	7.49	11.72
IGF1R	79.52	74.16	76.84
EGFR	26.52	22.20	24.36

## Data Availability

The data used in this study are available within the article and in the Supplementary Materials.

## Supplementary Materials

The following supporting information can be downloaded at: https://www.mdpi.com/article/10.3390/molecules29112653/s1. The supporting information includes characterization data (NMR spectra and HRMS spectra), along with the analysis of potential target proteins and predictions of drug-likeness.
