# Supplementary material for: Discovery of Ureido-Substituted 4-Phenylthiazole Derivatives as IGF1R Inhibitors with Potent Antiproliferative Properties
Source: Molecules. 2024 Jun 4;29(11):2653. doi: 10.3390/molecules29112653 (PMC11173463; doi:10.3390/molecules29112653)
Supplement: Supplementary file 1 [file molecules-29-02653-s001.zip › molecules-3012598-supplementary.pdf]

Discovery of ureido-substituted 3-phenylthiazole derivatives as IGF-1R inhibitors with potent antiproliferative properties

Yuan Tian[1], Ni An[2], Wenru Li[1], Shixin Tang[1], Jiqi Li[1], He Wang[1], Rongjian Su[2,*], Dong Cai[1,*,1]

[1] College of Pharmacy, Jinzhou Medical University, Jinzhou 121001, China;
[2] The Key Laboratory of Molecular and Cellular Biology and Drug Development in Universities of Liaoning Province, Jinzhou Medical University, Jinzhou 121001, China
* E-mail addresses: teachersurongjian@126.com (Rongjian Su); caid@jzmu.edu.cn (Dong Cai)

Table of Contents

1. ^{1}H and ^{13}C NMR of compound (**1~30**) ….………...………...……………...…S2

2. HRMS of compound (**1~30**)…...………...……...………..………...…..…...…S34

3. Analysis of potential target proteins for target compounds…….……….……...S43

4. Network pharmacology steps…………...…...…………... …...……...……...…S47

5. Drug-likeness prediction…………...…...…………...……...……..………...…S48

1. ^{1}H and ^{13}C NMR of compound (1~30)

4-(3-nitrophenyl)thiazol-2-amine (**1**):

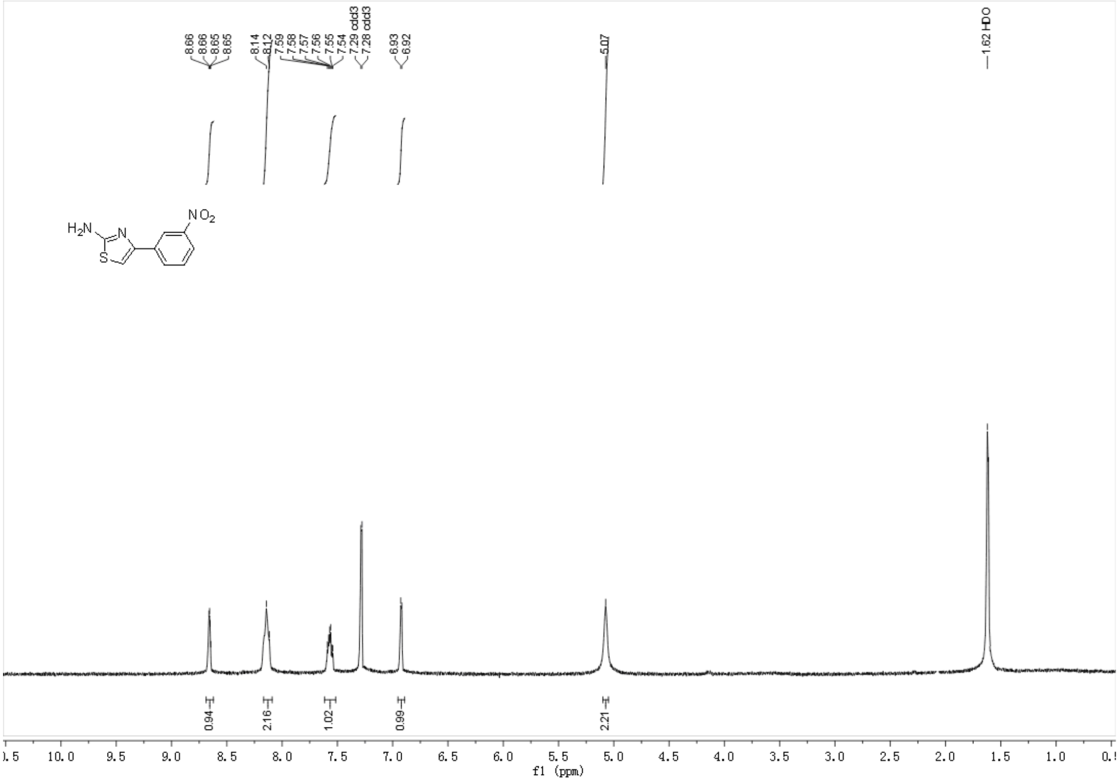

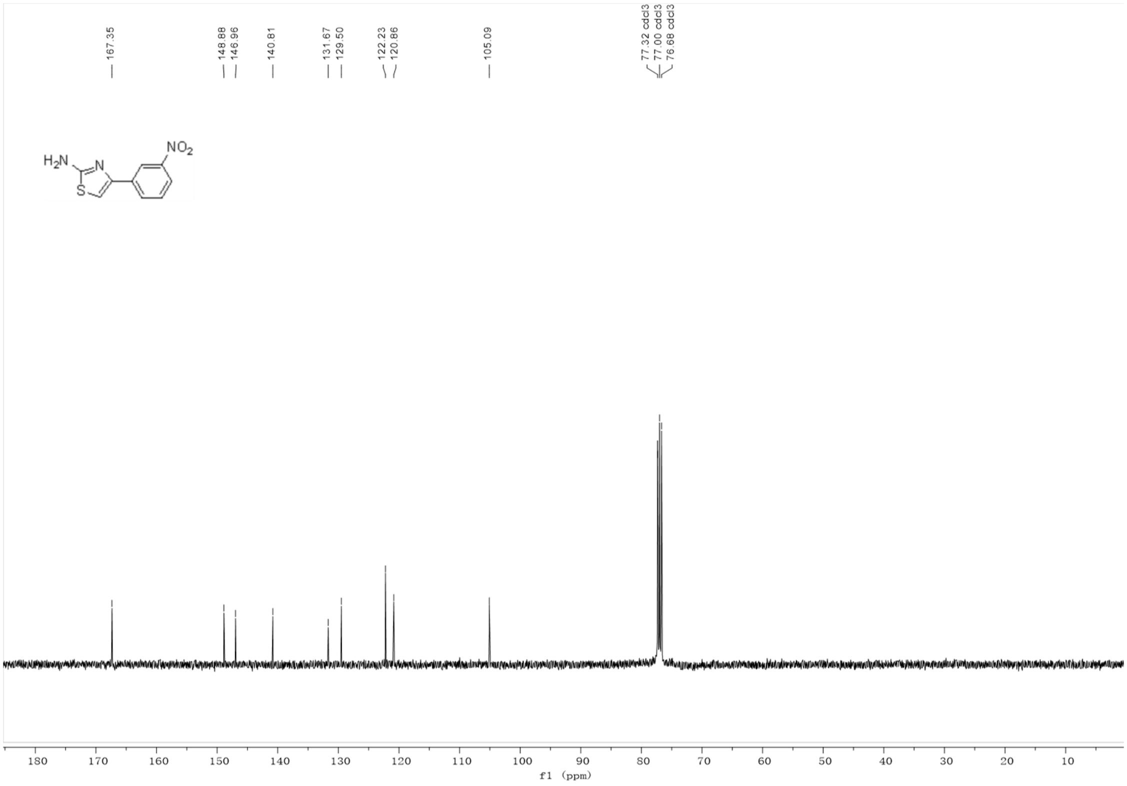

2-chloro-*N*-(4-(3-nitrophenyl)thiazol-2-yl)acetamide (**2**):

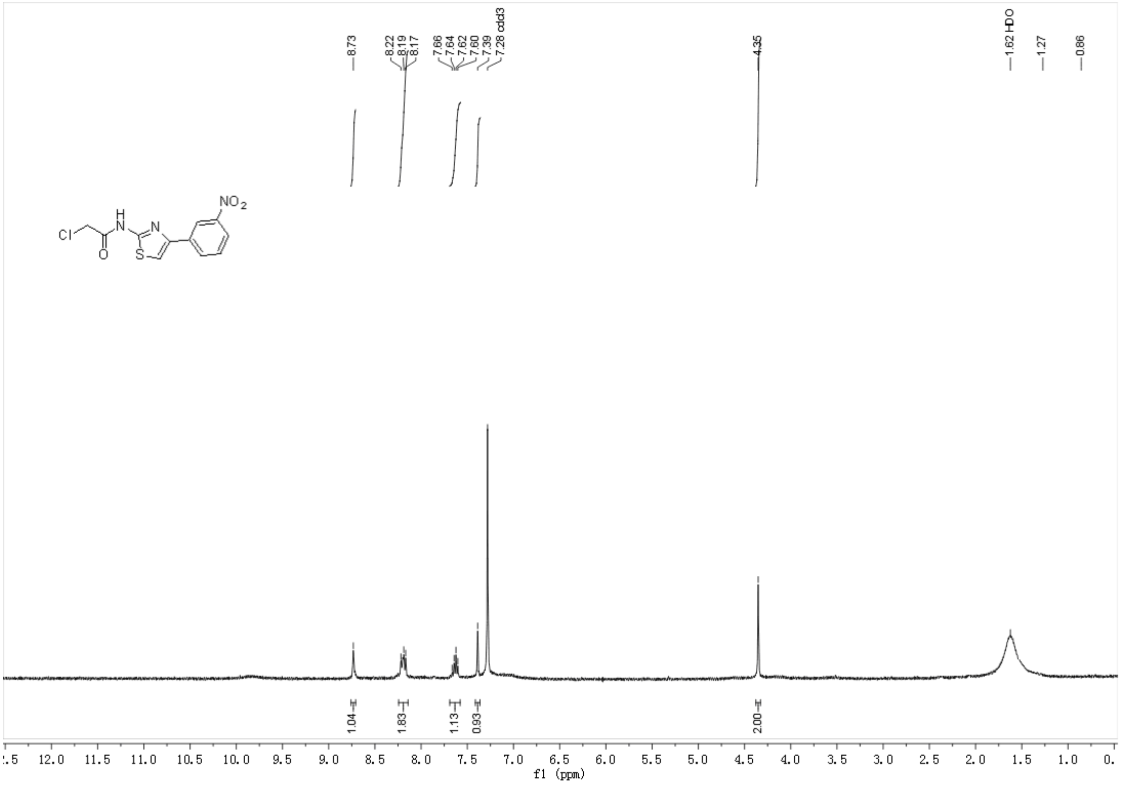

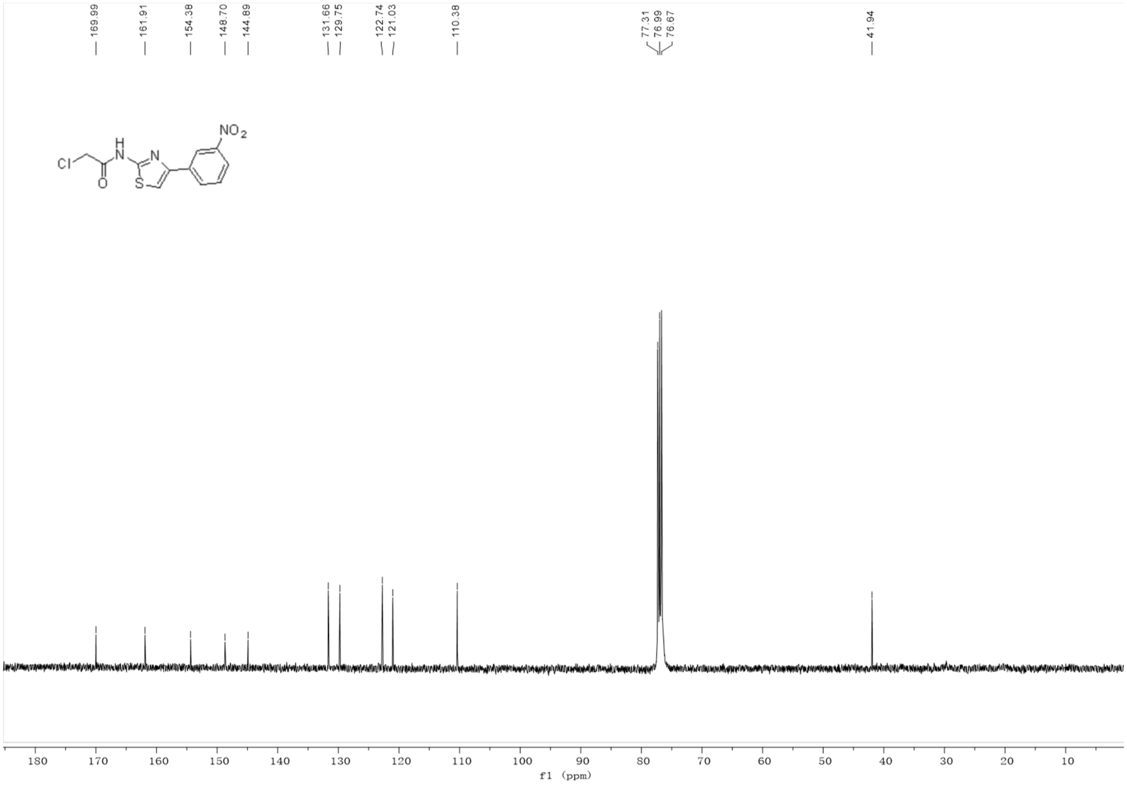

2-morpholino-*N*-(4-(3-nitrophenyl)thiazol-2-yl)acetamide (**3a**):

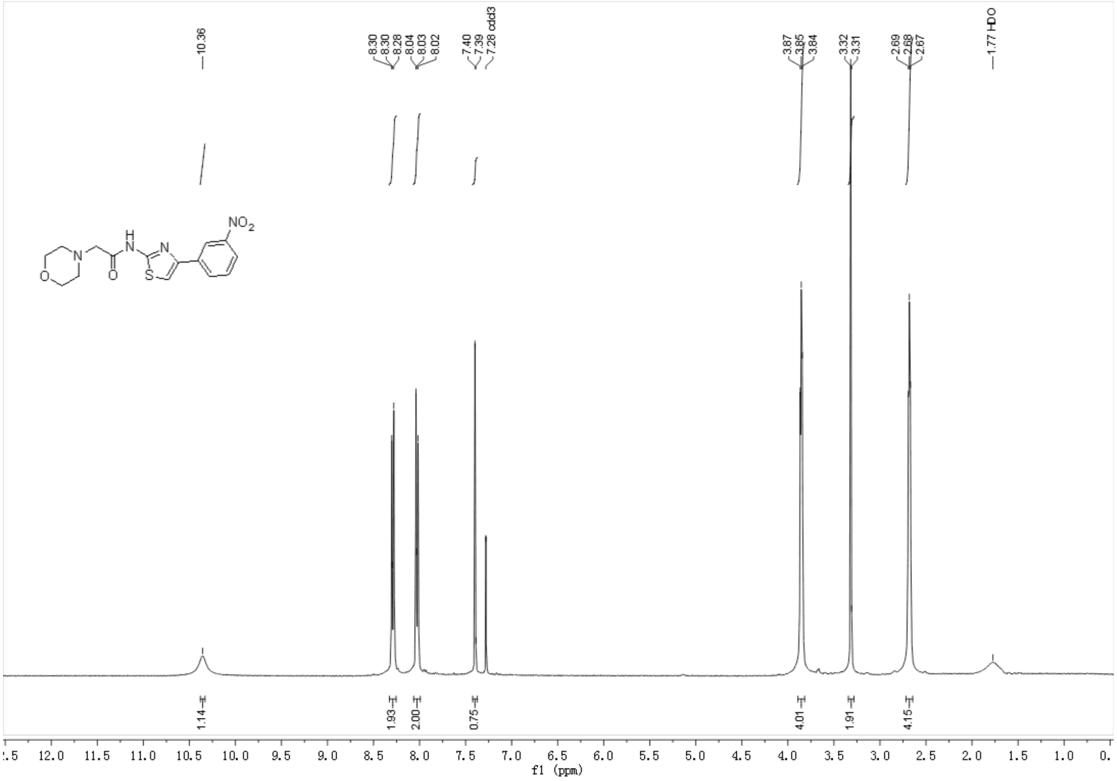

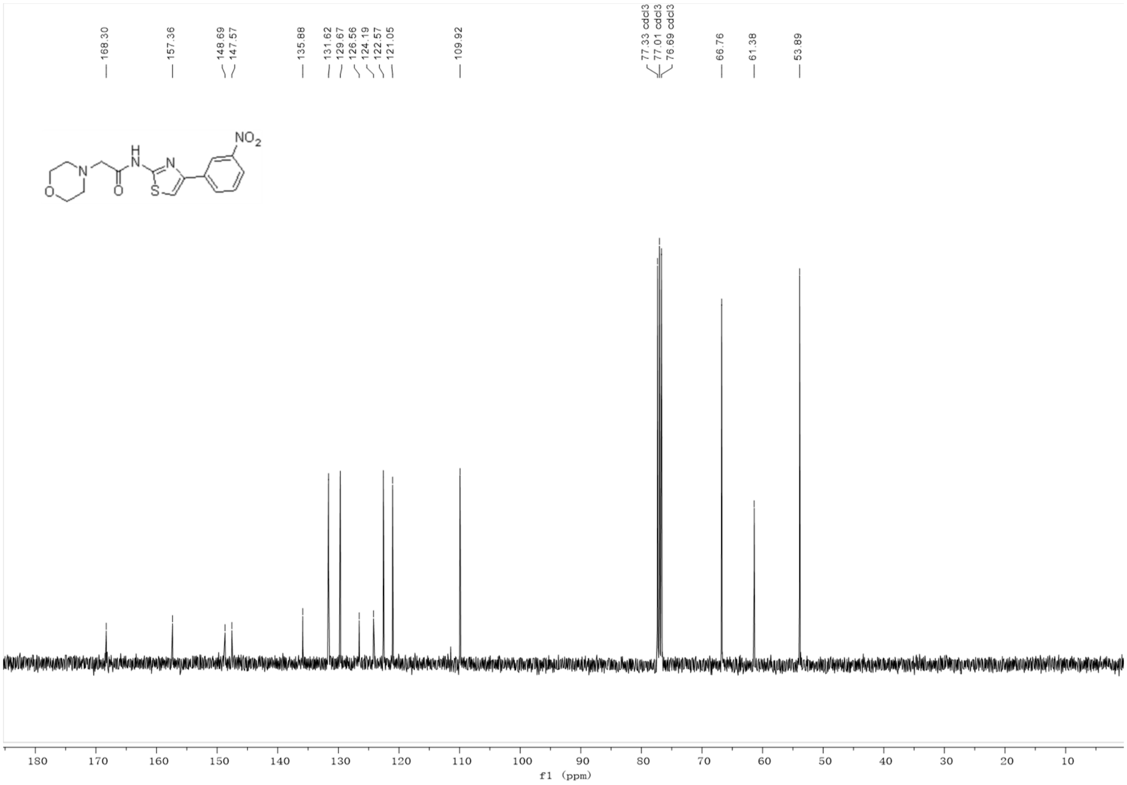

N-(4-(3-nitrophenyl)thiazol-2-yl)-2-(piperidin-1-yl)acetamide (**3b**):

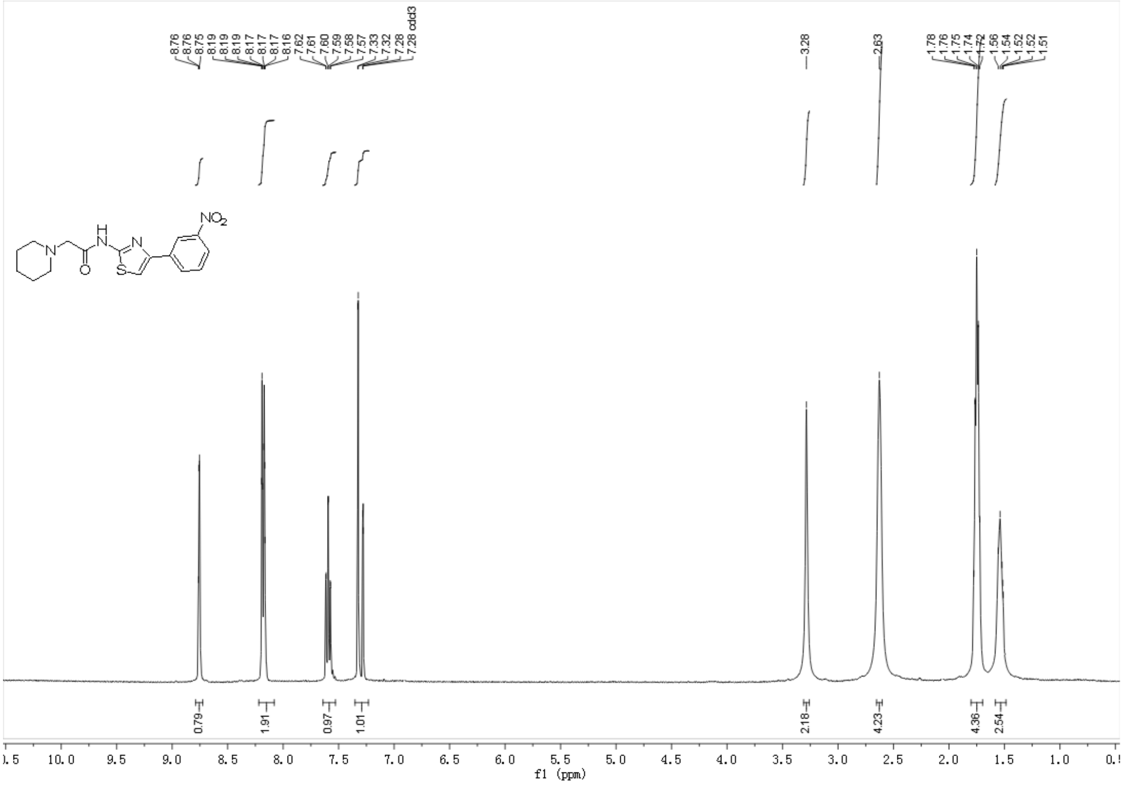

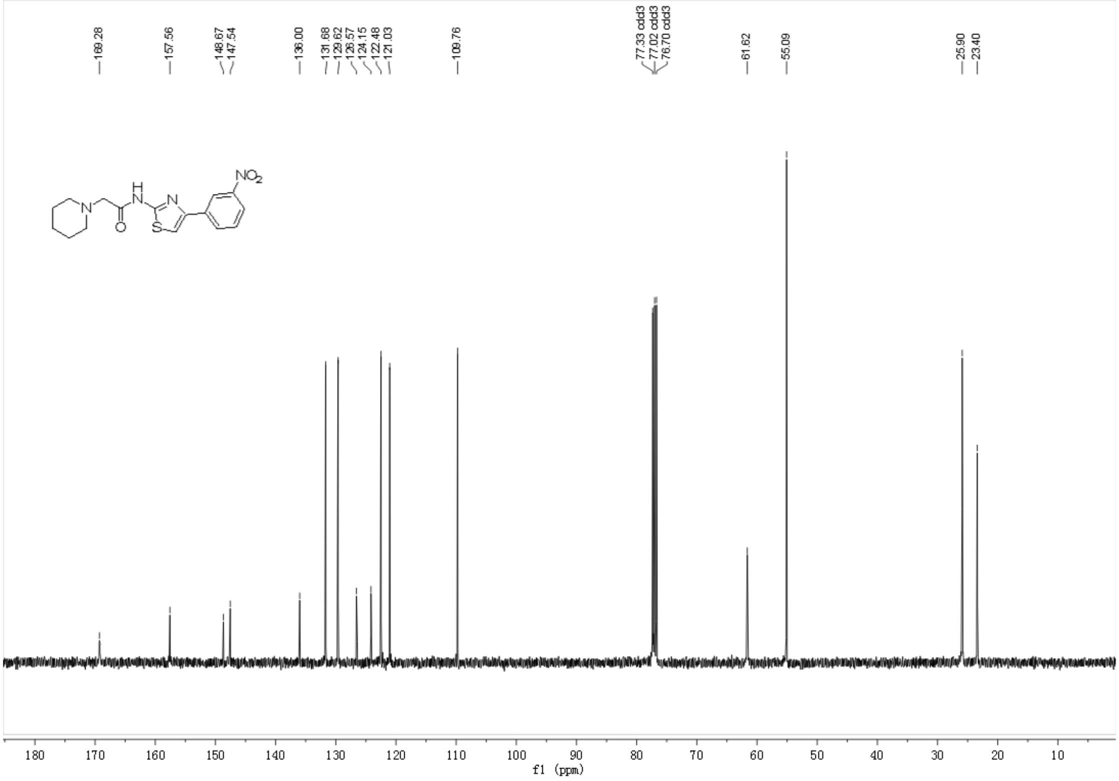

N-(4-(3-aminophenyl)thiazol-2-yl)-2-morpholinoacetamide (**4a**):

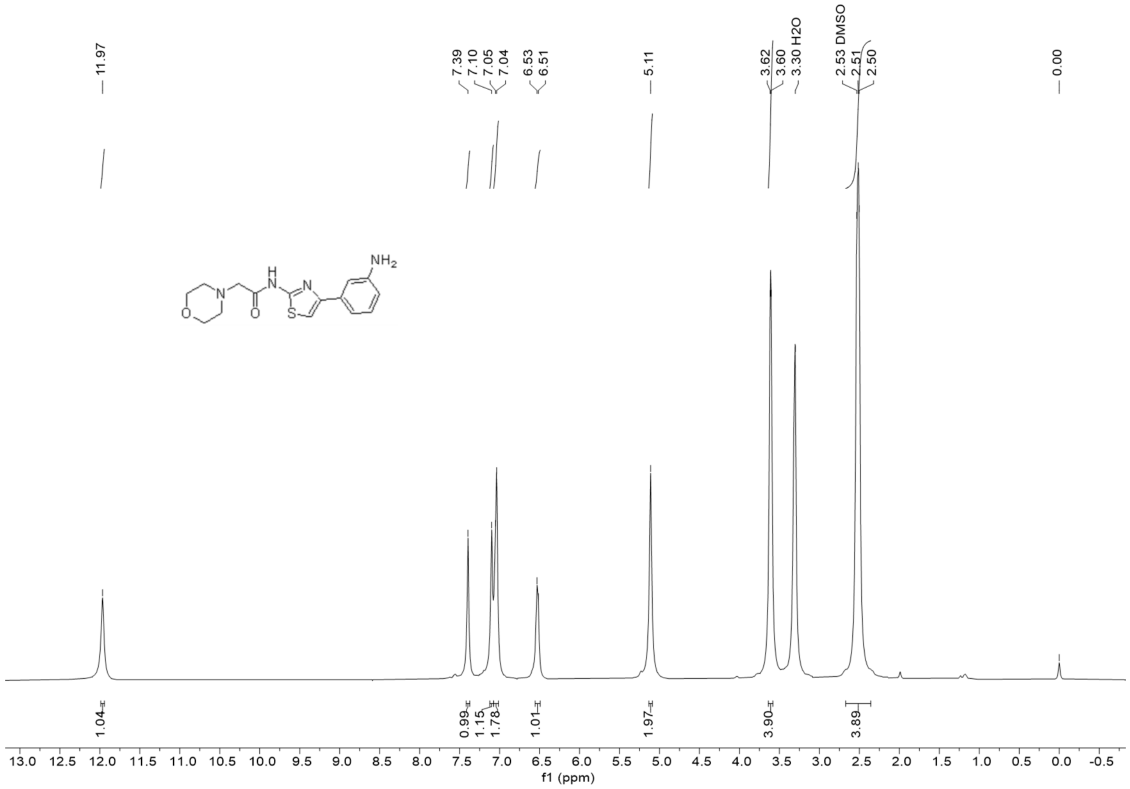

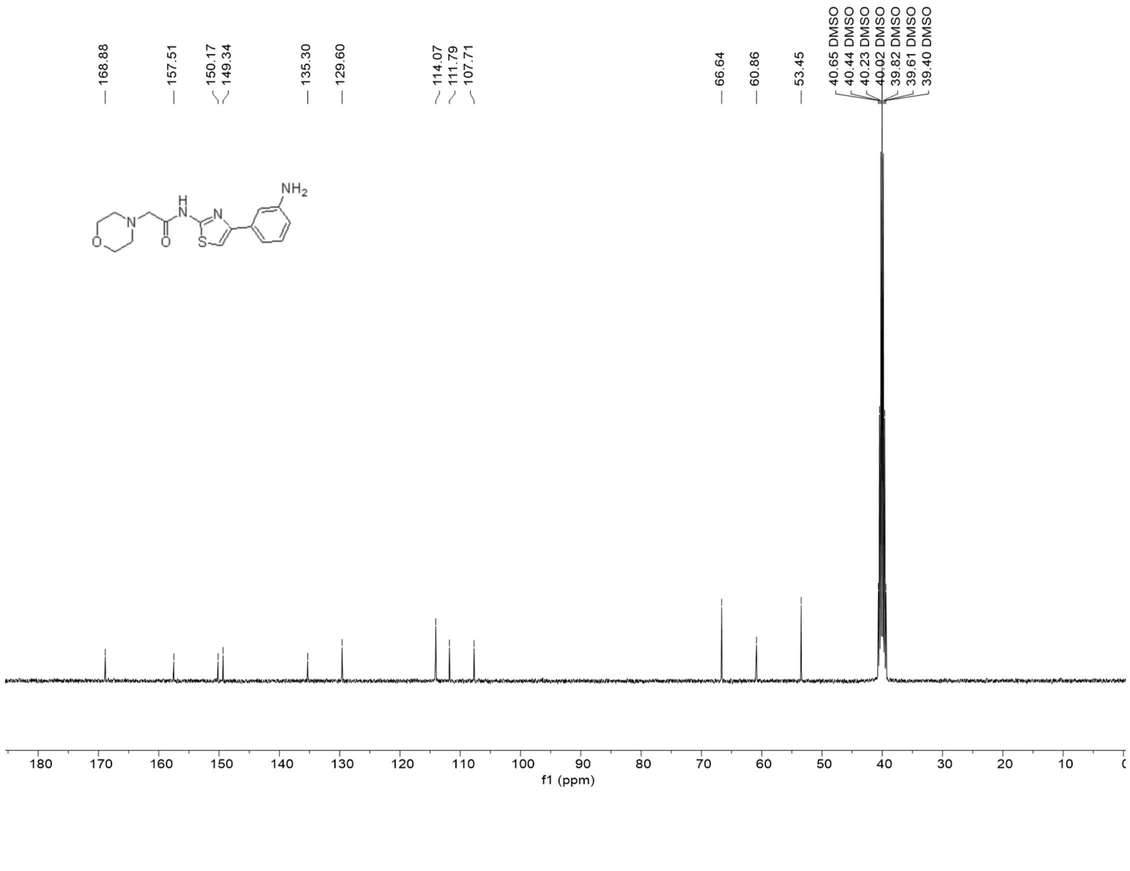

N-(4-(3-aminophenyl)thiazol-2-yl)-2-(piperidin-1-yl)acetamide (**4b**):

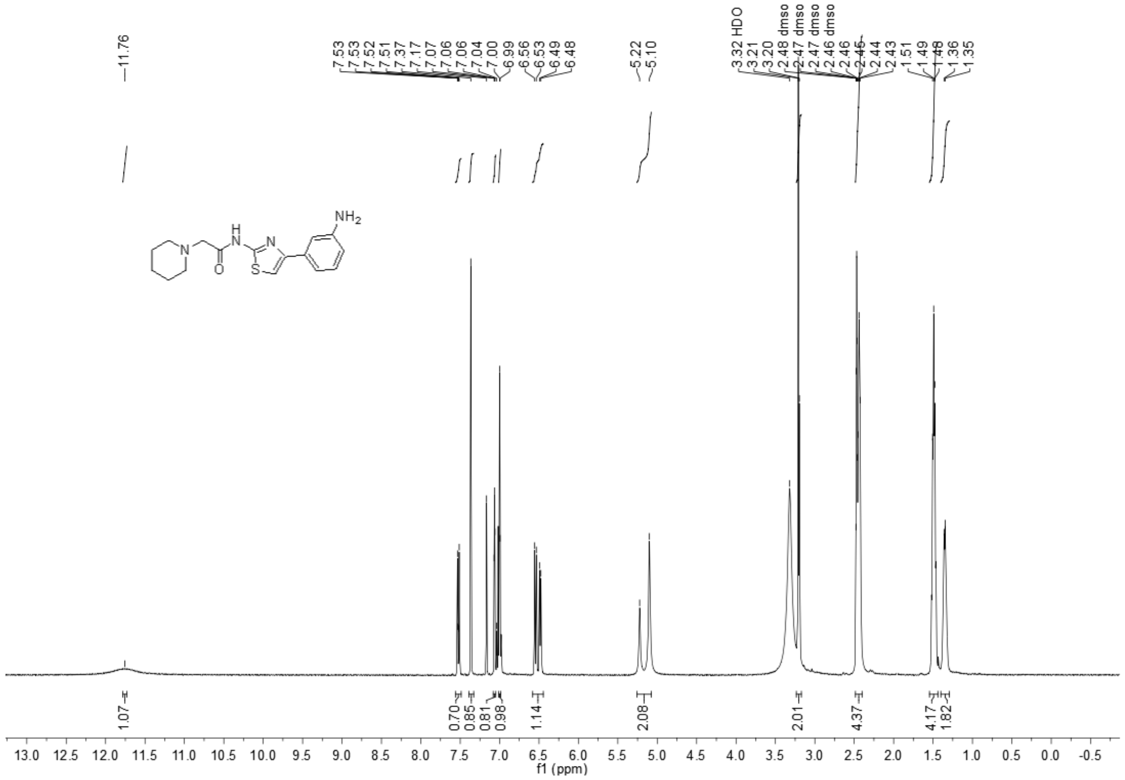

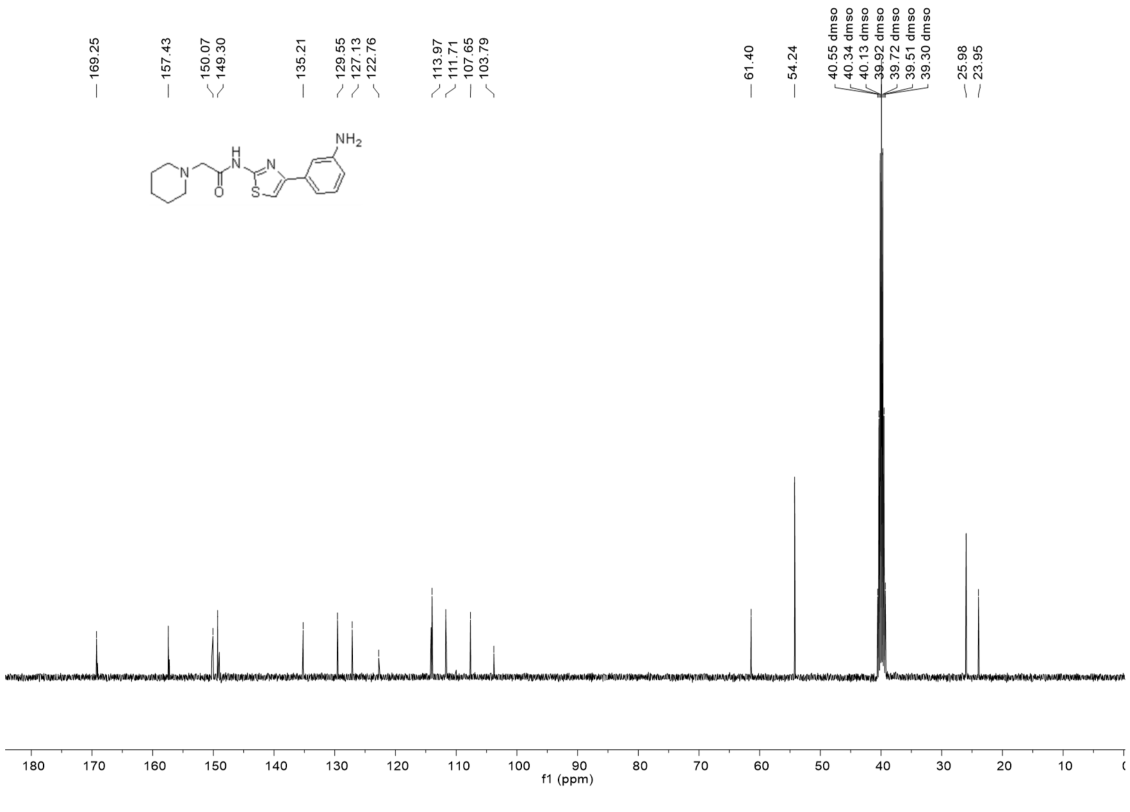

N-(4-(3-(3-cyclohexylureido)phenyl)thiazol-2-yl)-2-morpholinoacetamide (**5**):

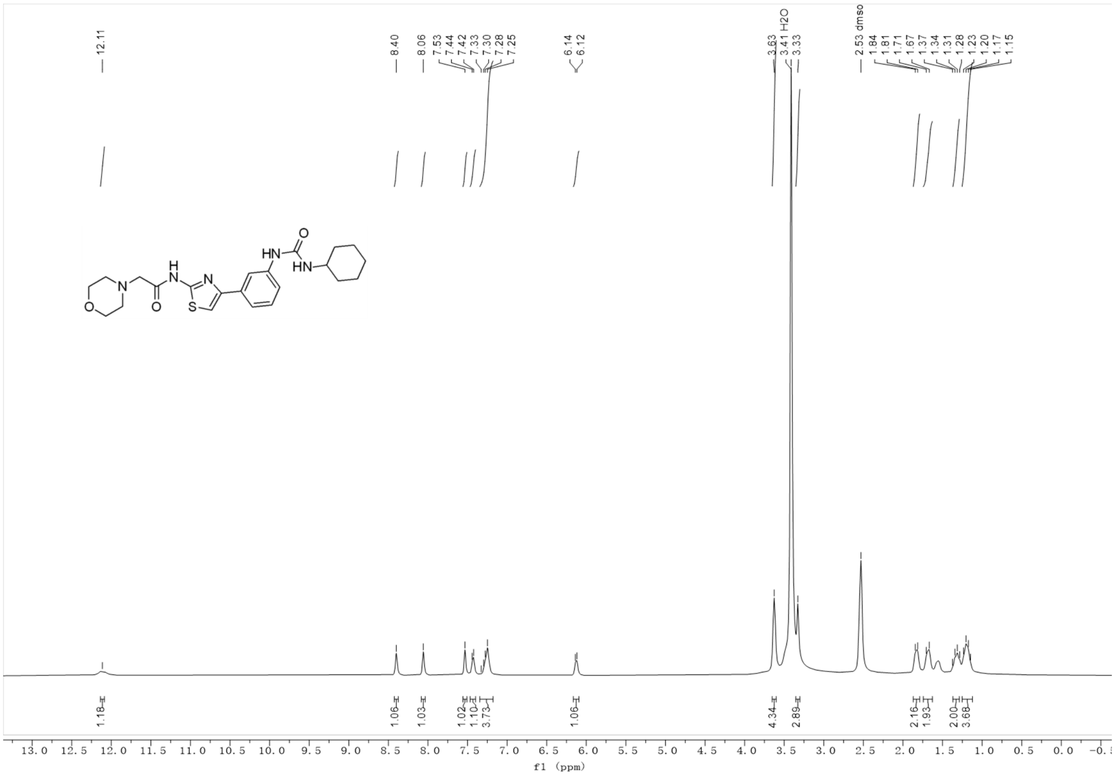

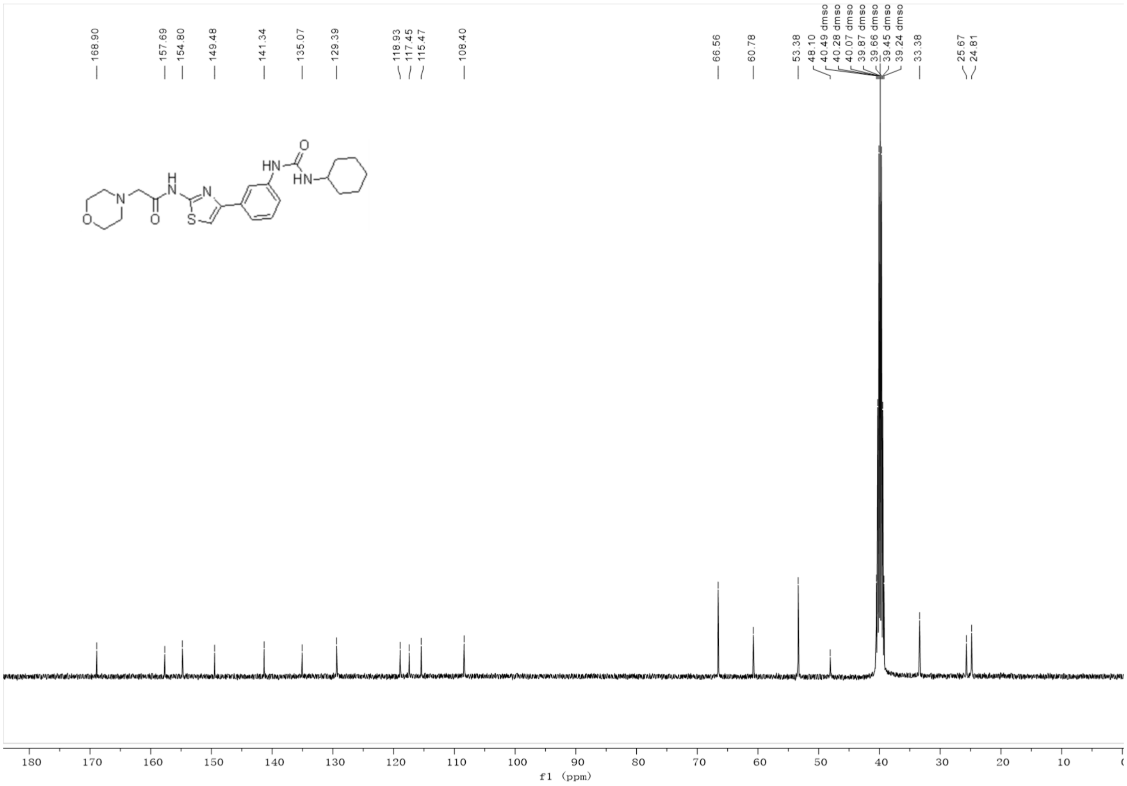

N-(4-(3-(3-(4-chlorophenyl)ureido)phenyl)thiazol-2-yl)-2-morpholinoacetamide (**6**):

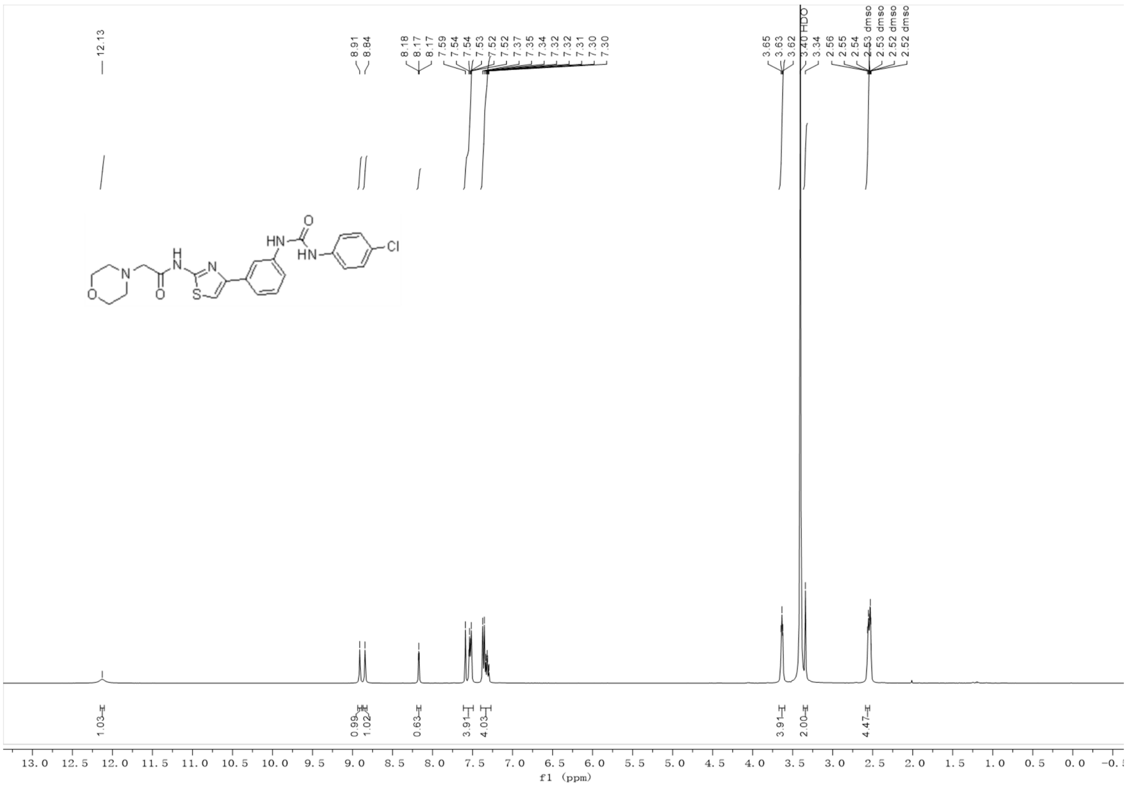

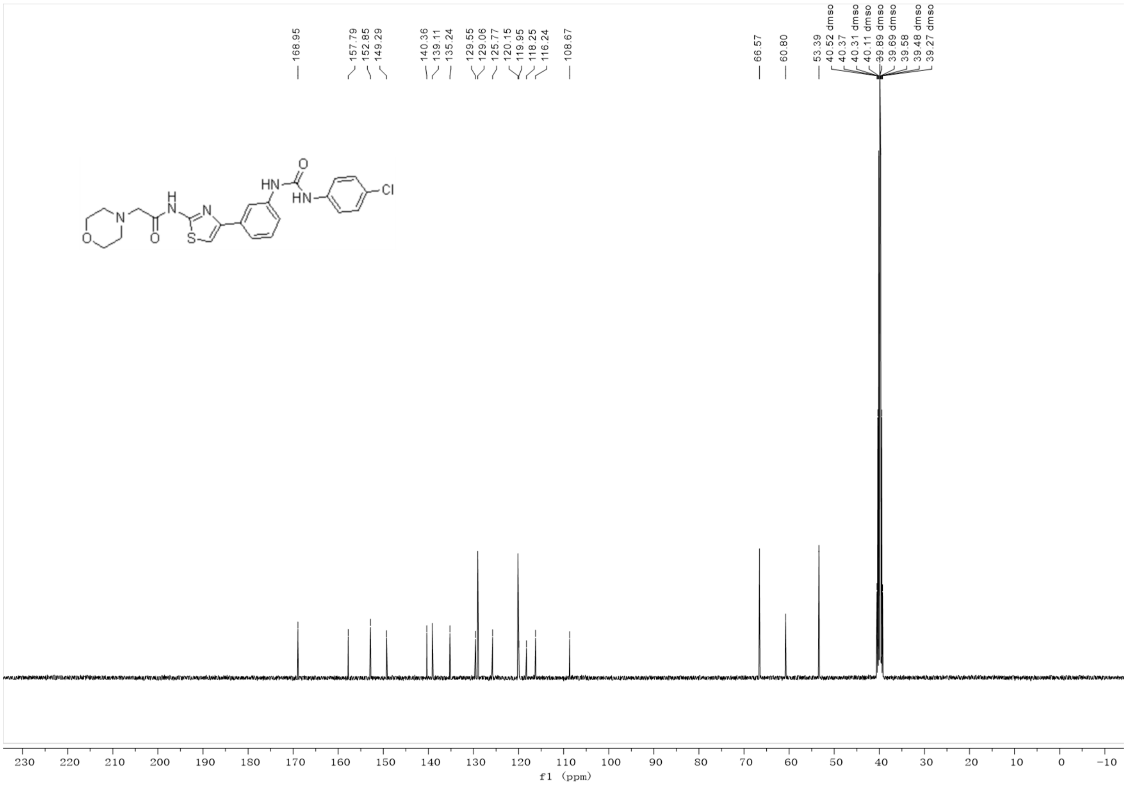

N-(4-(3-(3-(3-chlorophenyl)ureido)phenyl)thiazol-2-yl)-2-morpholinoacetamide (**7**):

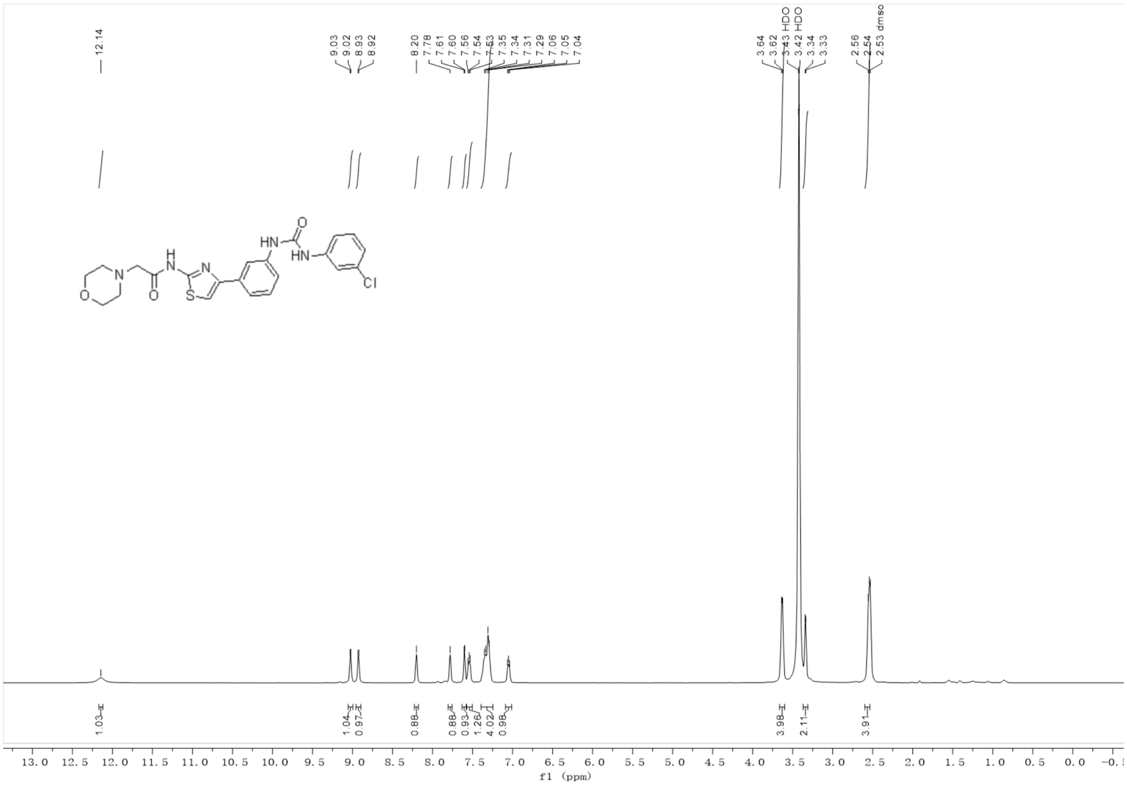

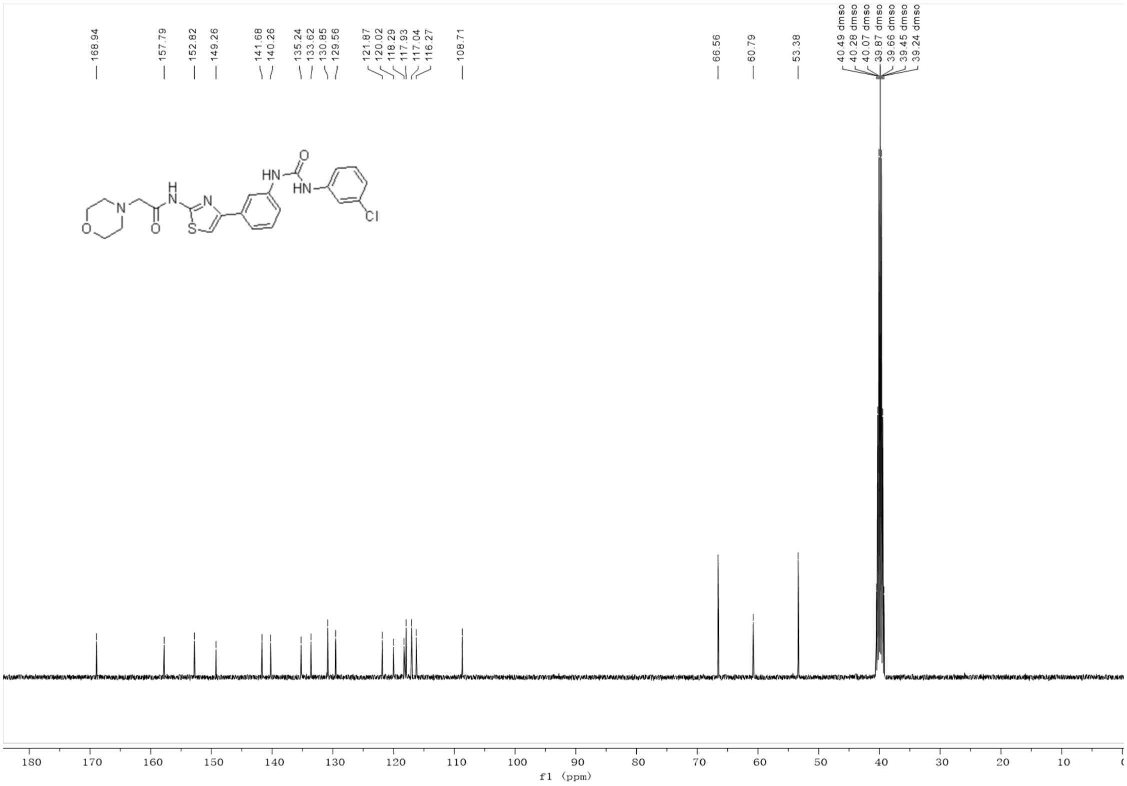

N-(4-(3-(3-(2,4-dichlorophenyl)ureido)phenyl)thiazol-2-yl)-2-morpholin-4-ylacetamide (**8**)

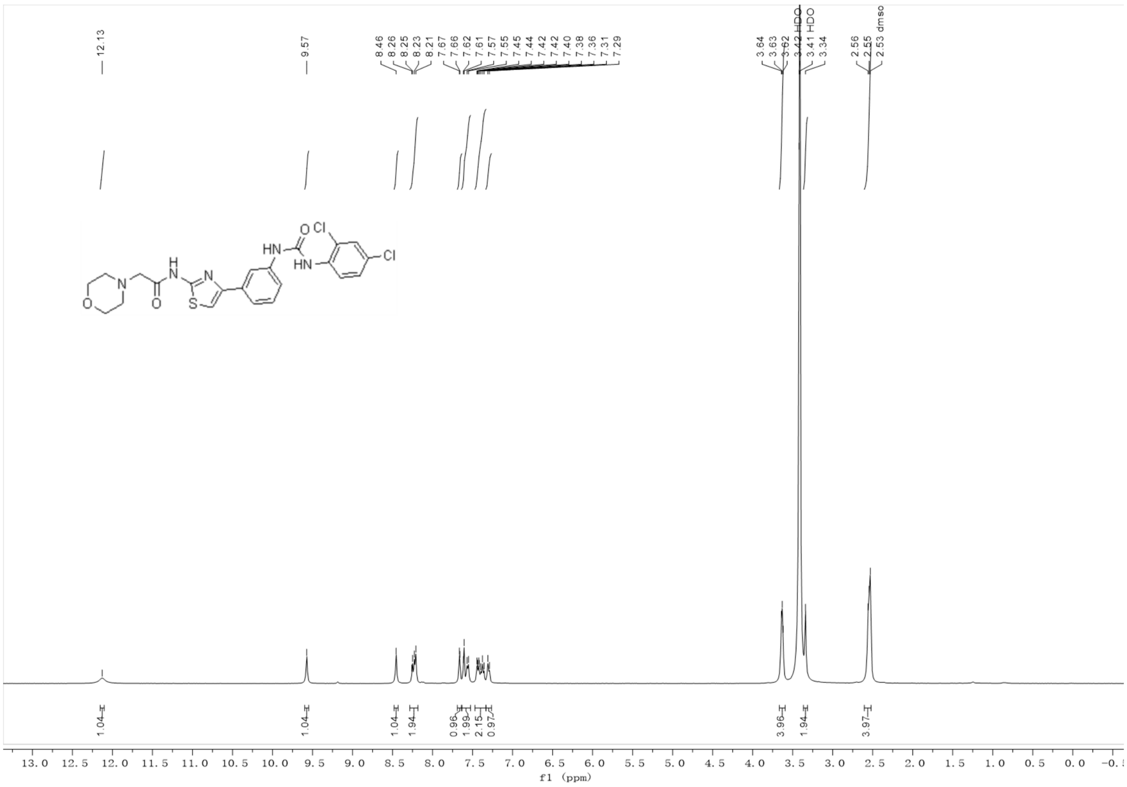

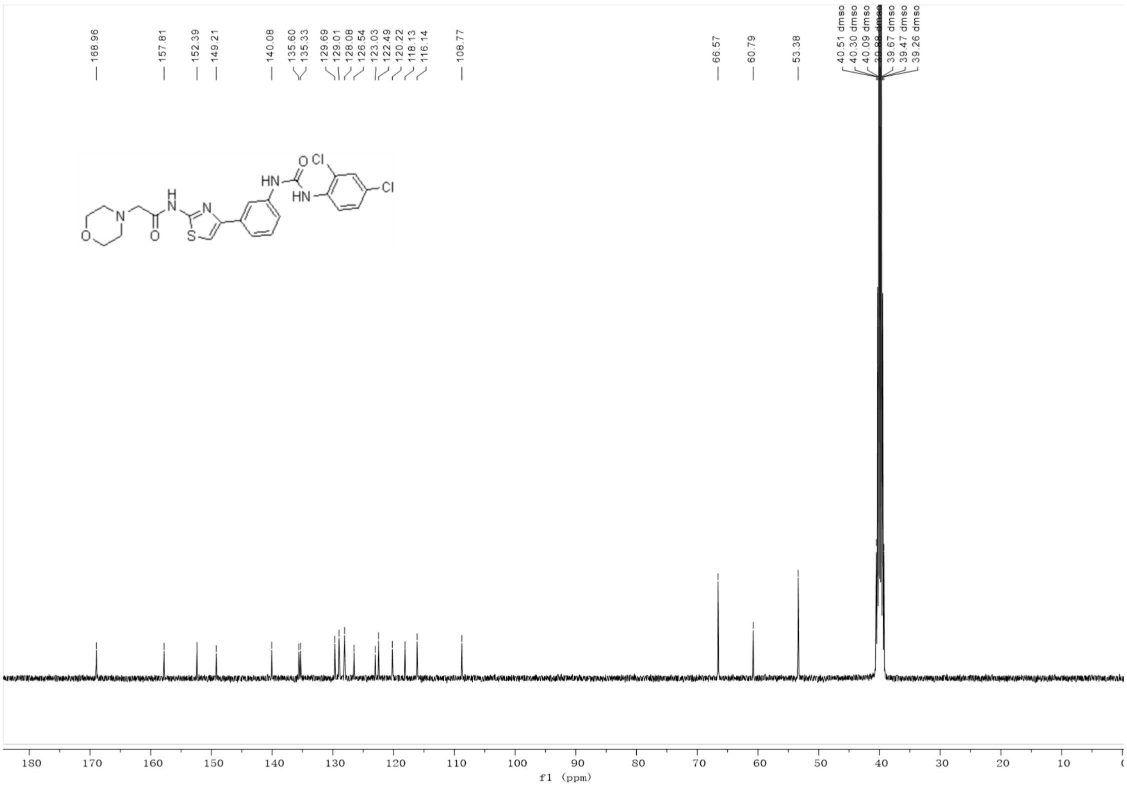

N-(4-(3-(3-(3,4-dichlorophenyl)ureido)phenyl)thiazol-2-yl)-2-morpholinoacetamide (**9**):

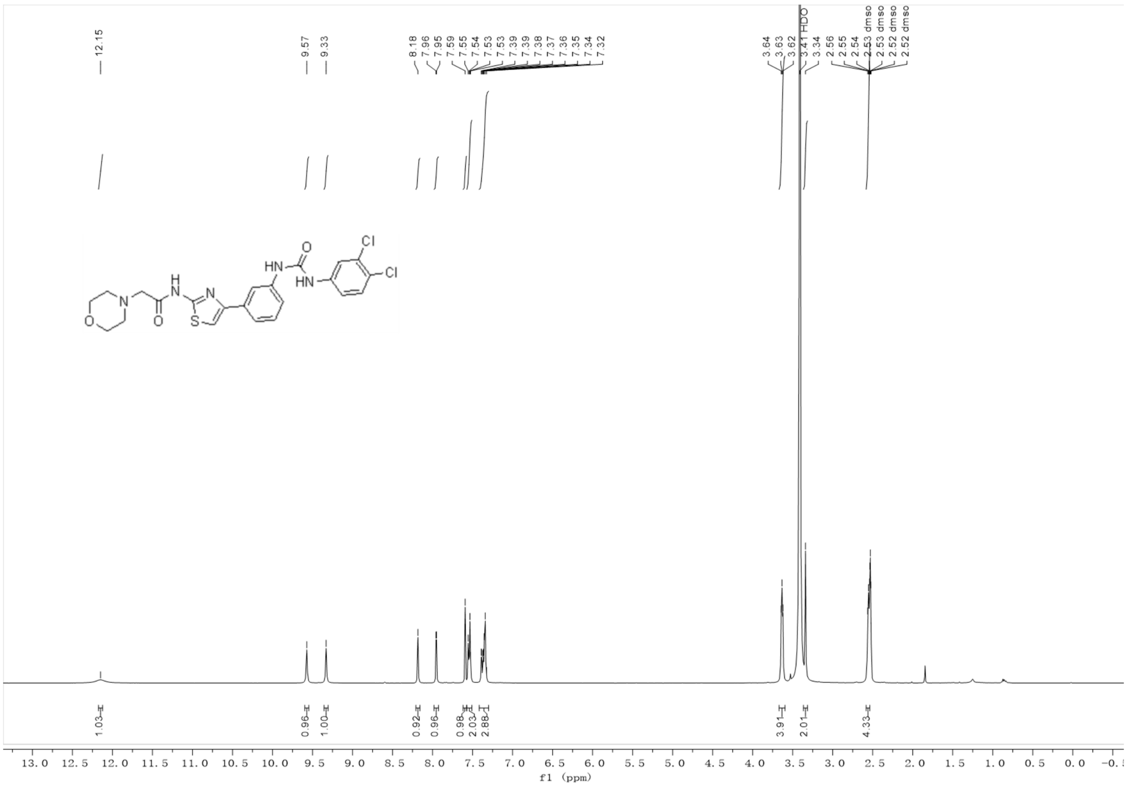

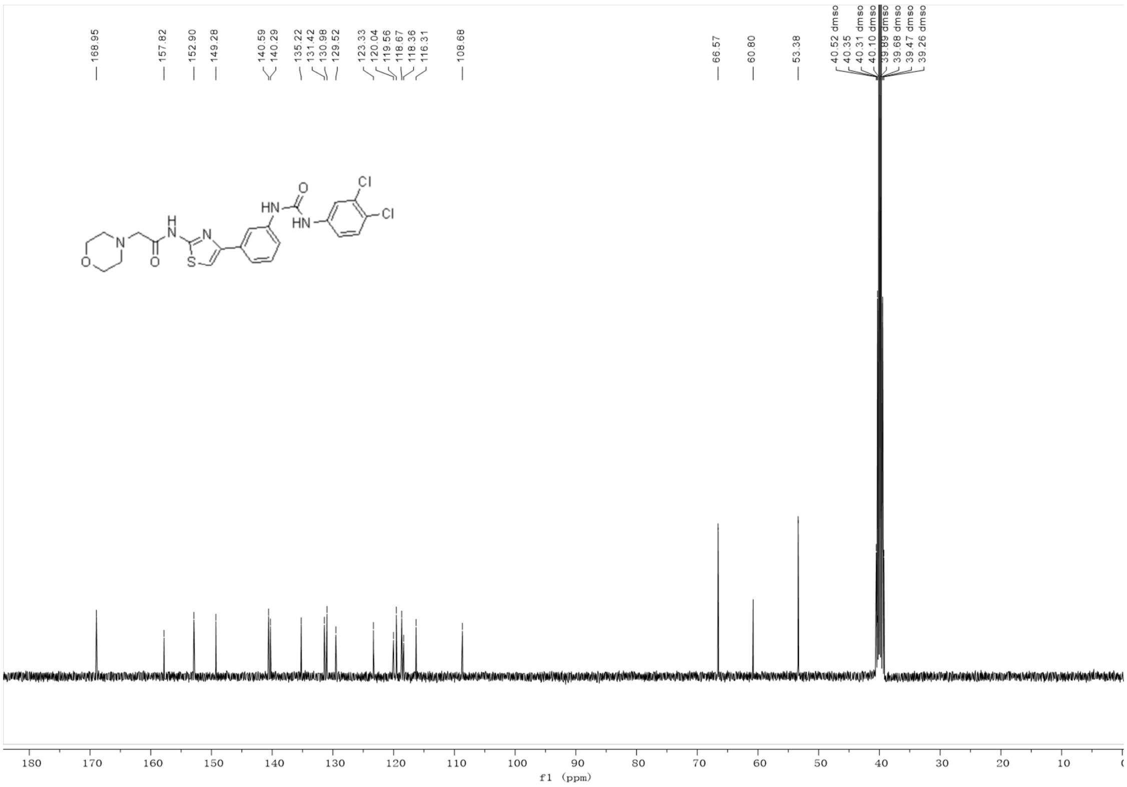

N-(4-(3-(3-(3,5-dichlorophenyl)ureido)phenyl)thiazol-2-yl)-2-morpholinoacetamide (**10**):

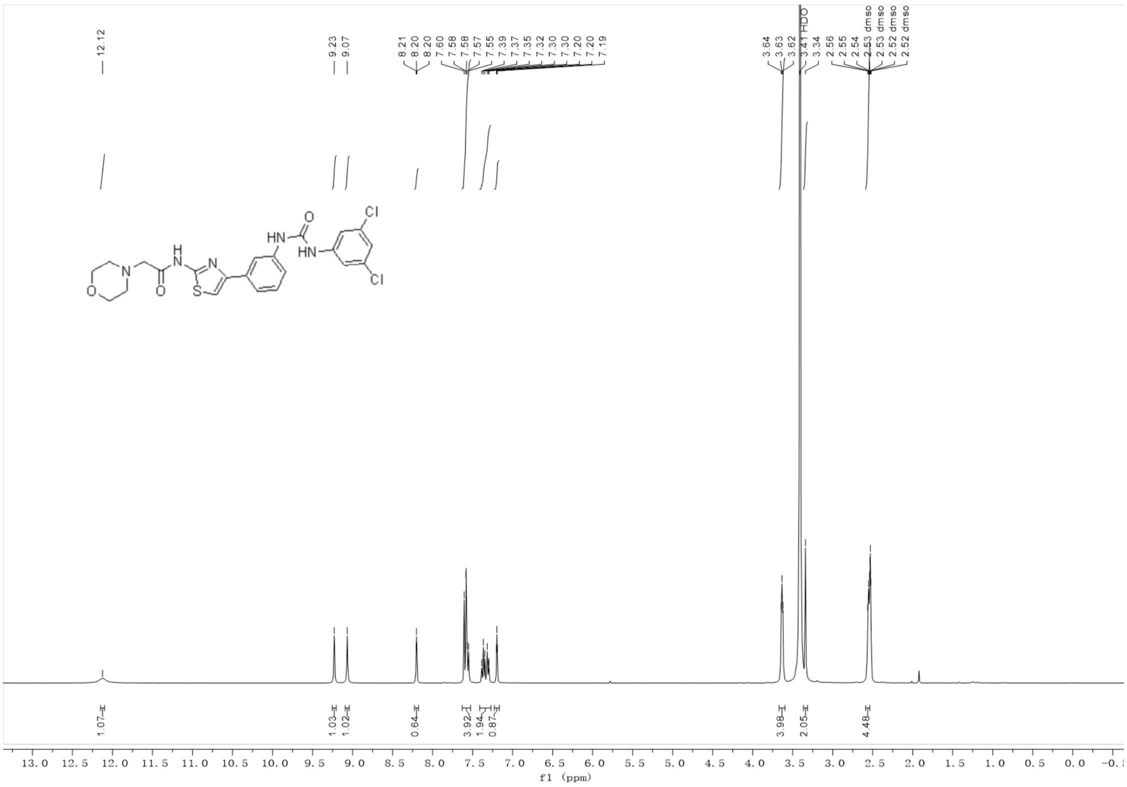

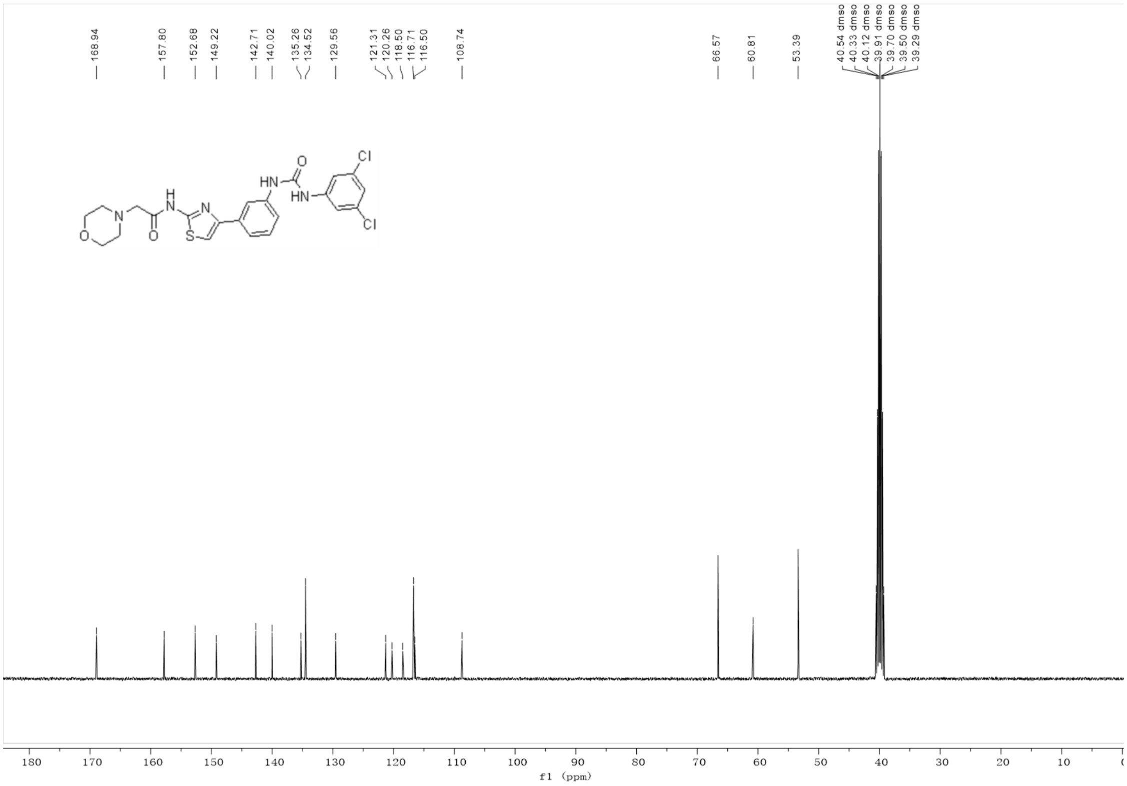

N-(4-(3-(3-(3-chloro-4-methylphenyl)ureido)phenyl)thiazol-2-yl)-2-morpholinoacetamide (**11**):

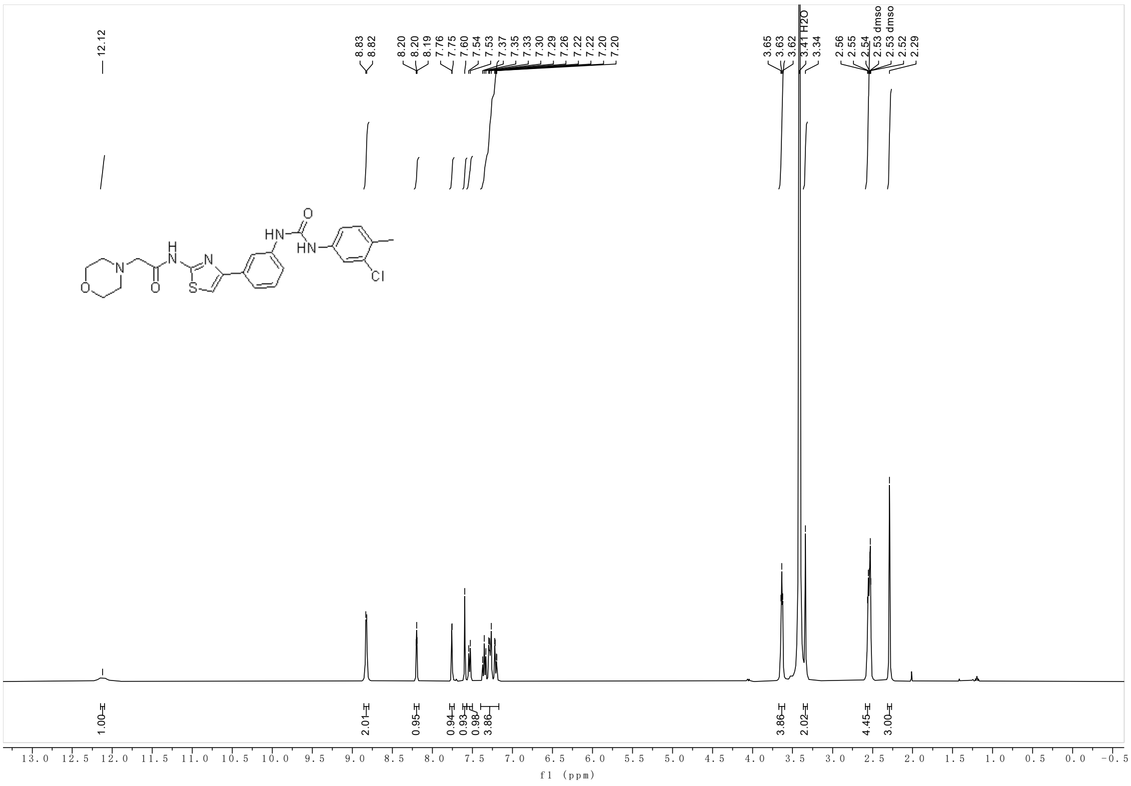

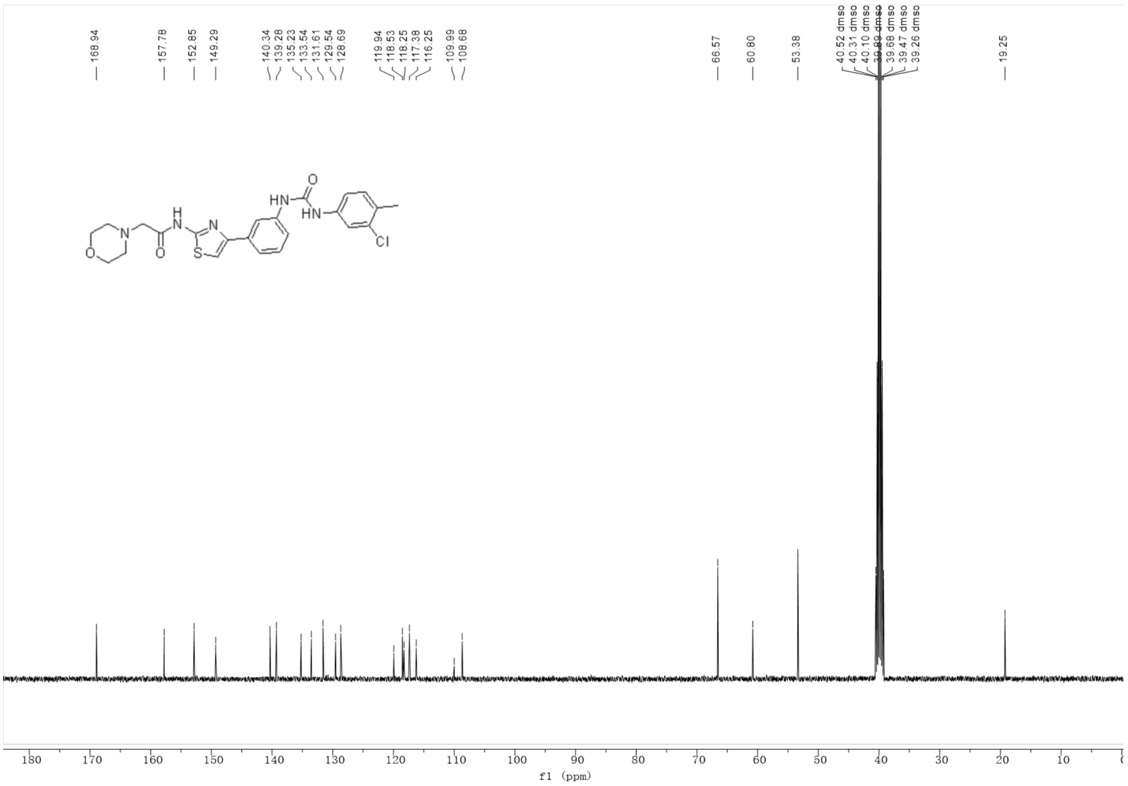

N-(4-(3-(3-(4-chloro-3-(trifluoromethyl)phenyl)ureido)phenyl)thiazol-2-yl)-2-morpholinoacetamide

(**12**):

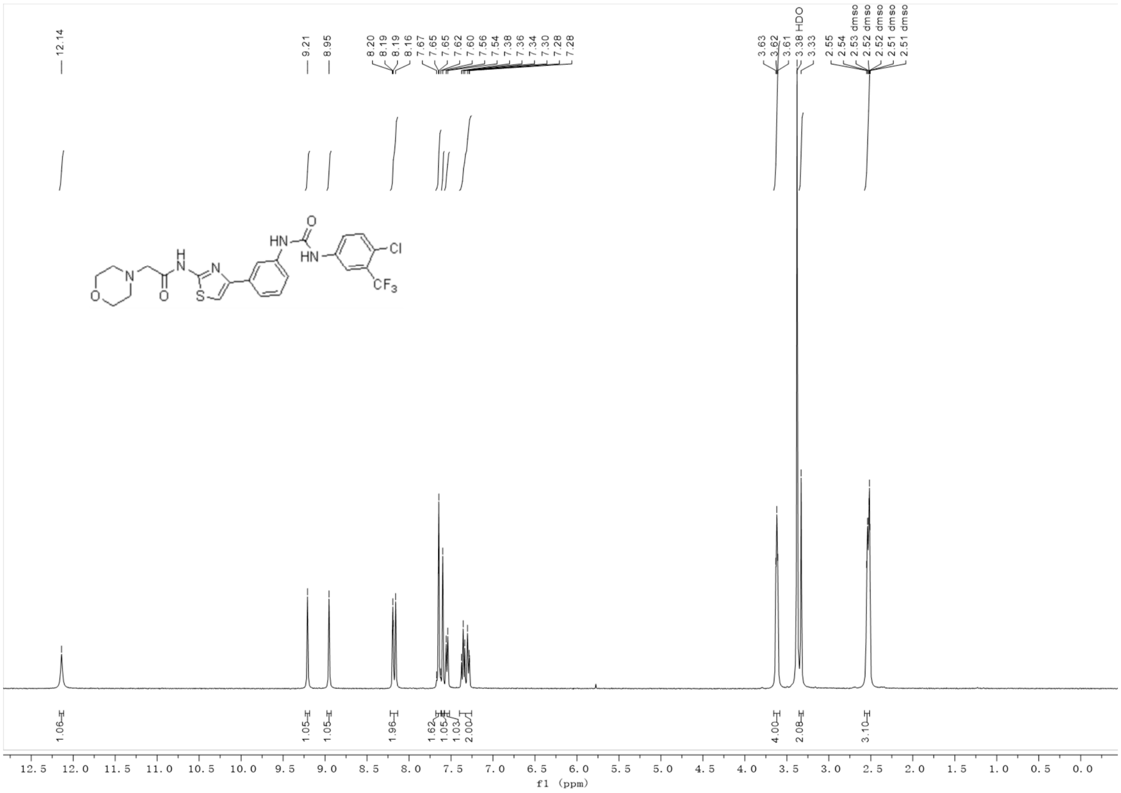

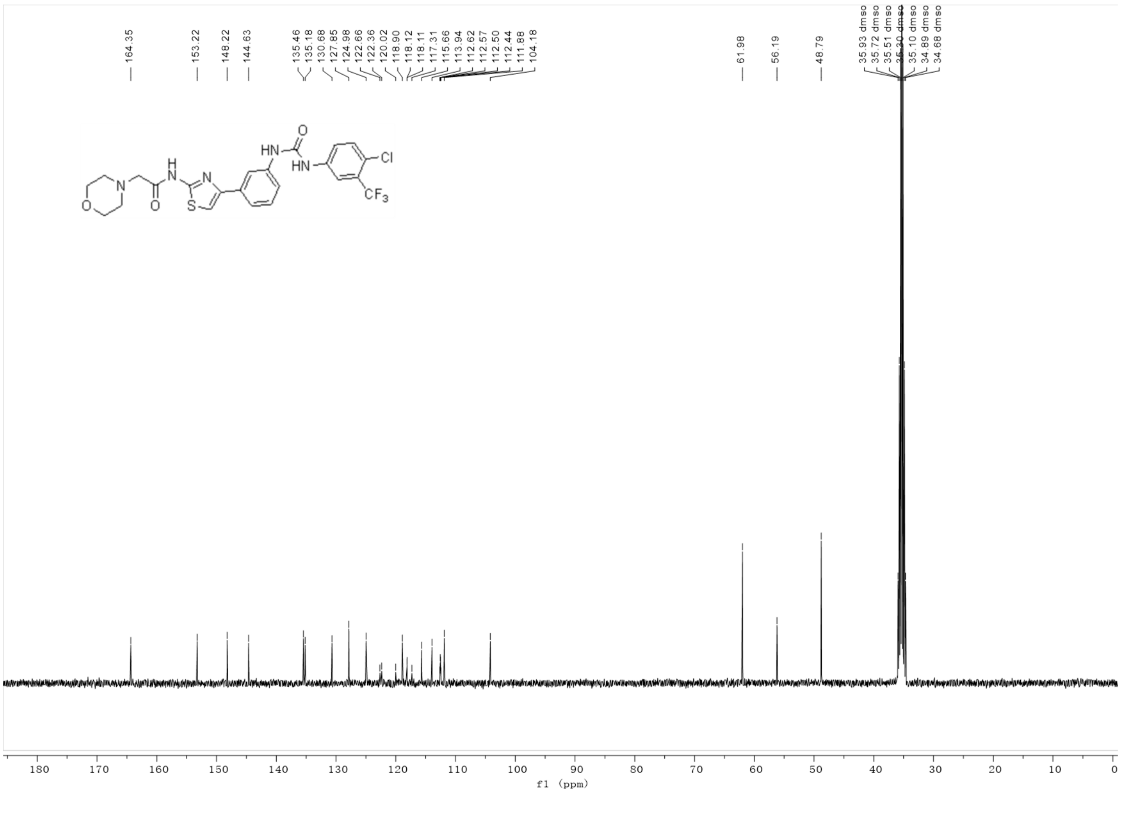

N-(4-(3-(3-(4-bromophenyl)ureido)phenyl)thiazol-2-yl)-2-morpholinoacetamide (**13**):

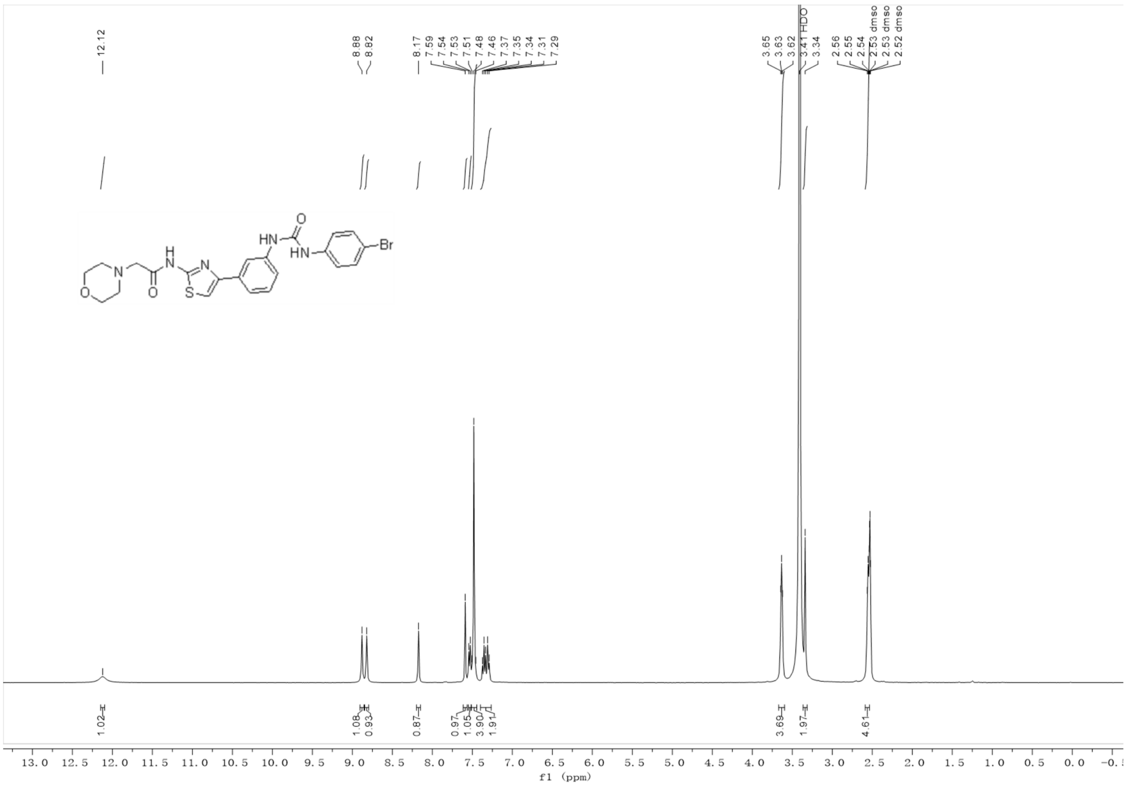

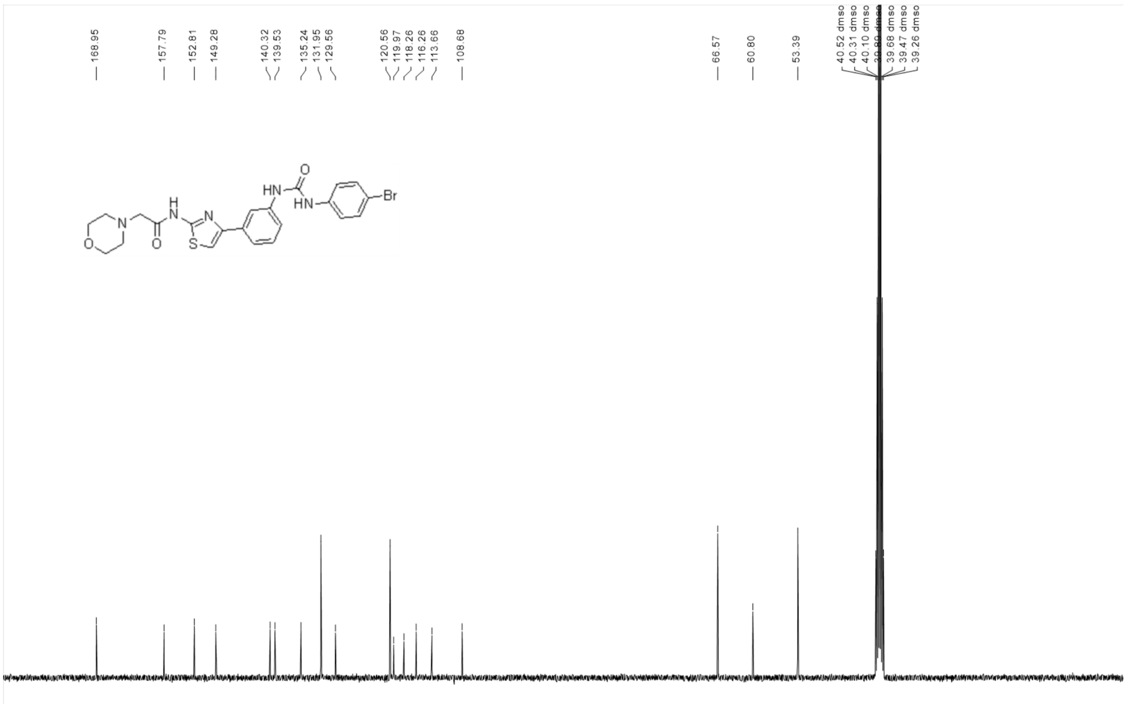

N-(4-(3-(3-(4-fluorophenyl)ureido)phenyl)thiazol-2-yl)-2-morpholinoacetamide (**14**):

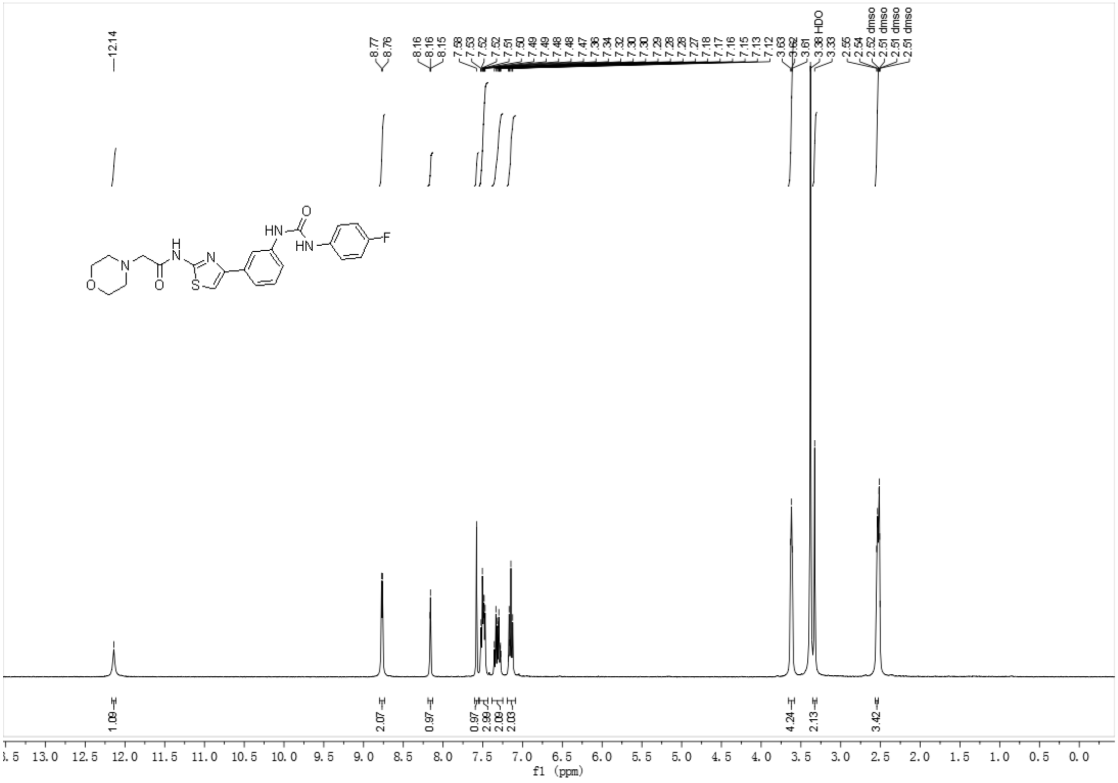

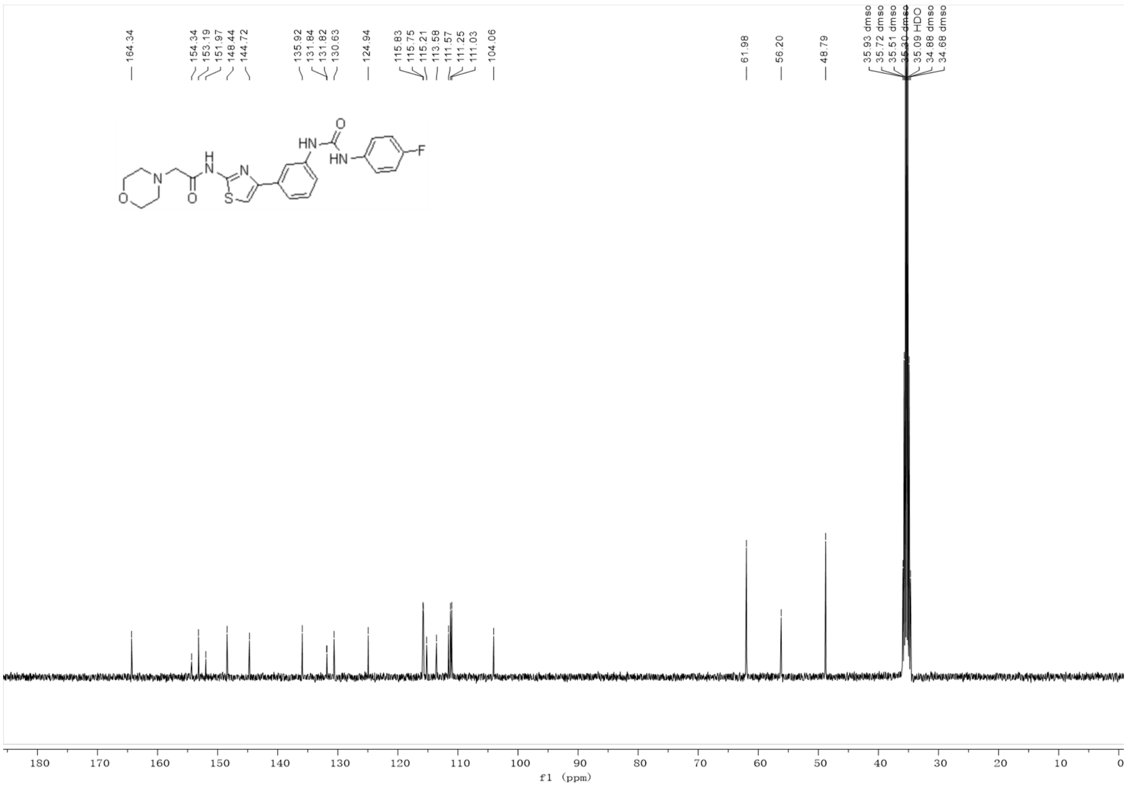

N-(4-(3-(3-(2,4-difluorophenyl)ureido)phenyl)thiazol-2-yl)-2-morpholinoacetamide (**15**):

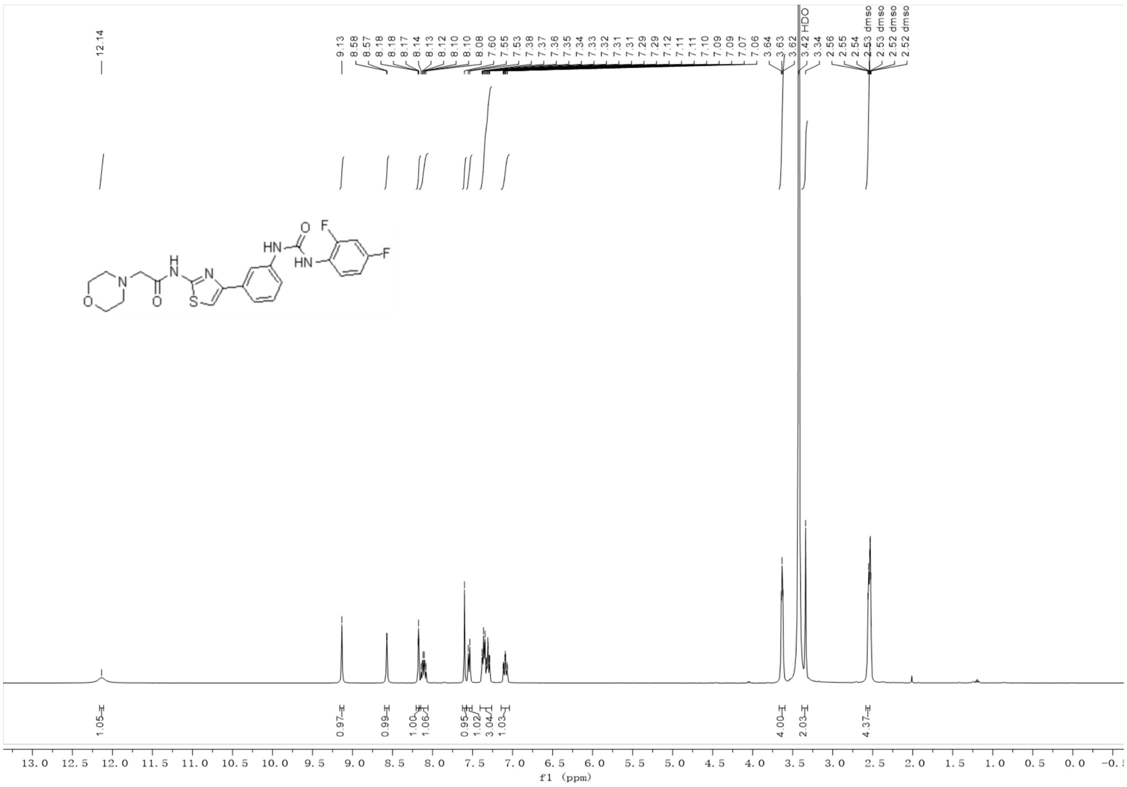

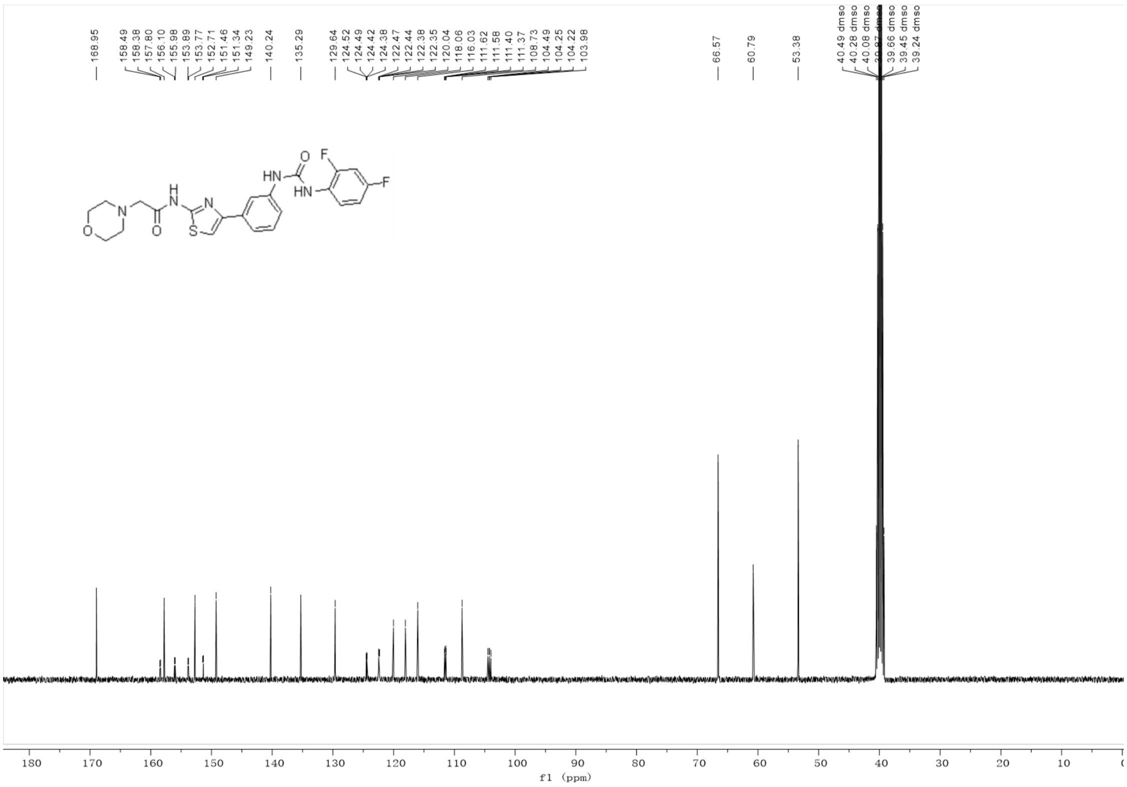

2-morpholino-*N*-(4-(3-(3-(4-(trifluoromethyl)phenyl)ureido)phenyl)thiazol-2-yl)acetamide (**16**):

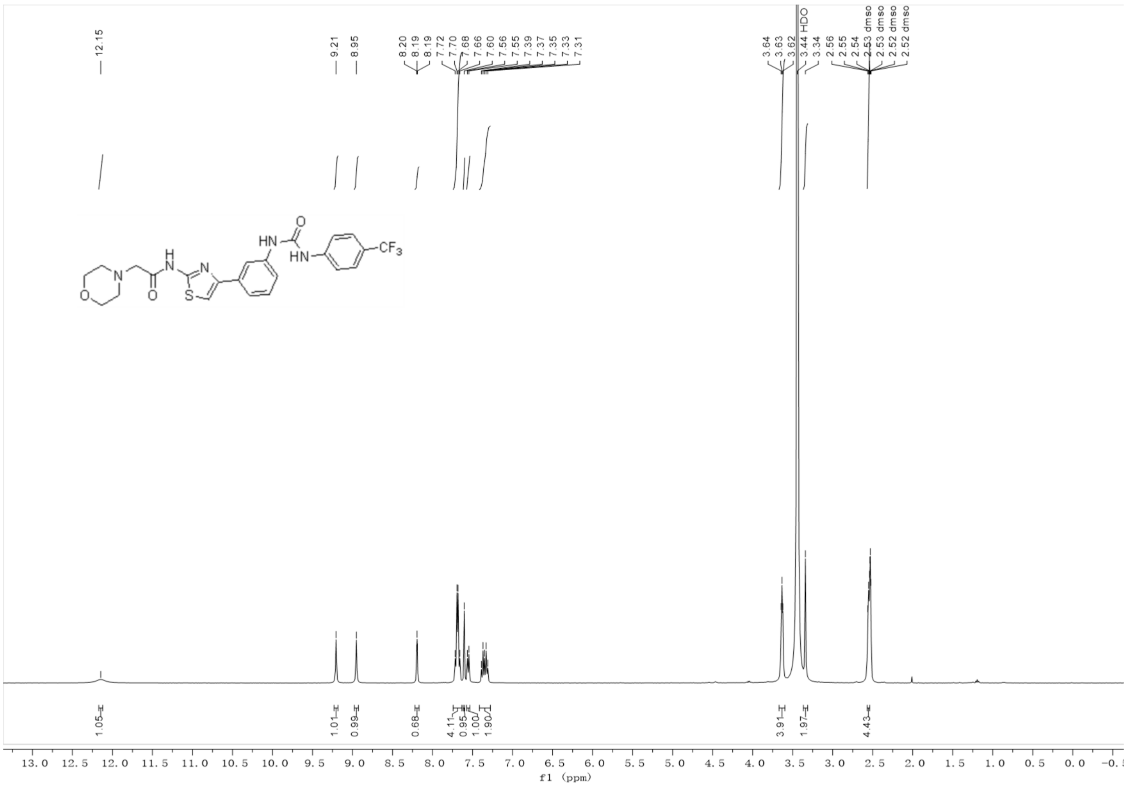

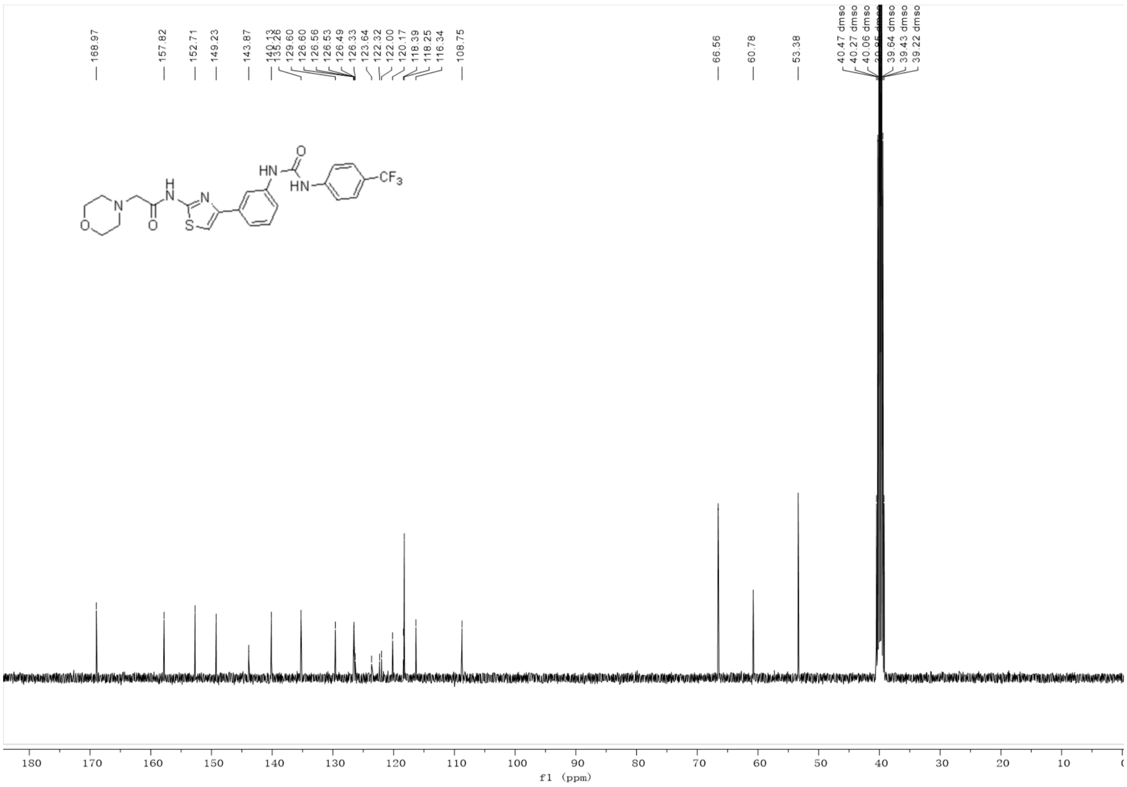

2-morpholino-*N*-(4-(3-(3-(m-tolyl)ureido)phenyl)thiazol-2-yl)acetamide (**17**):

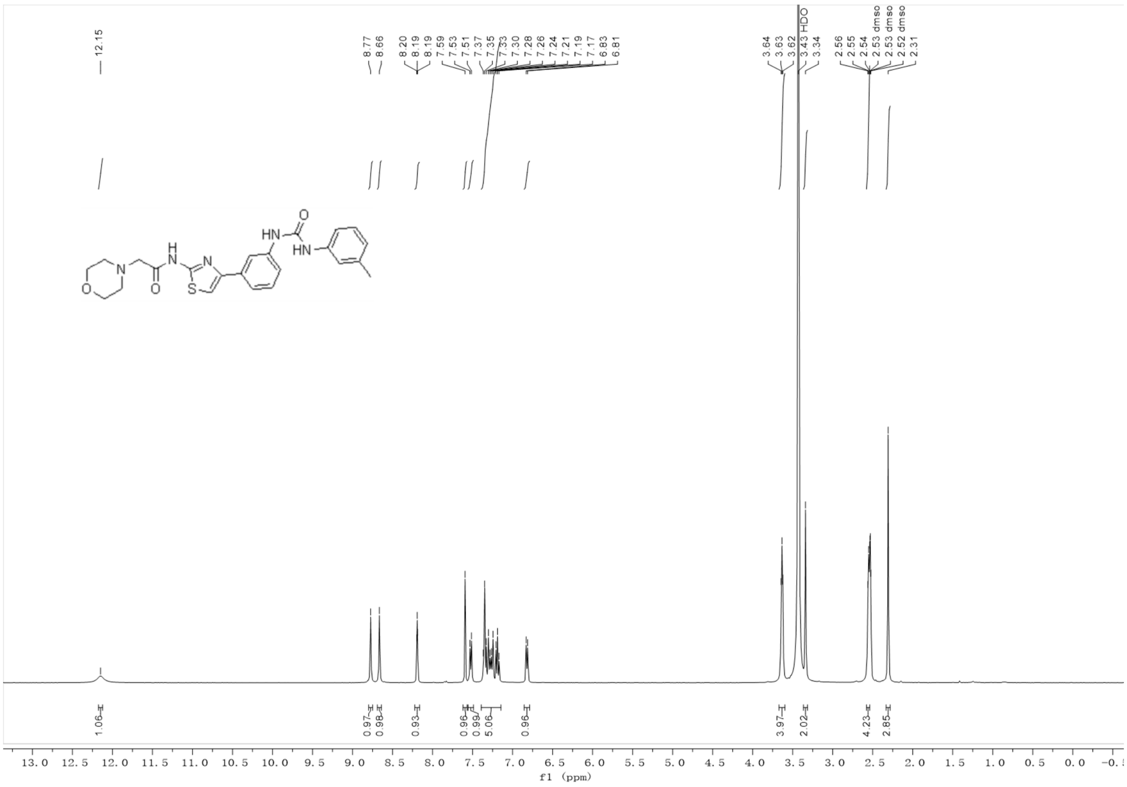

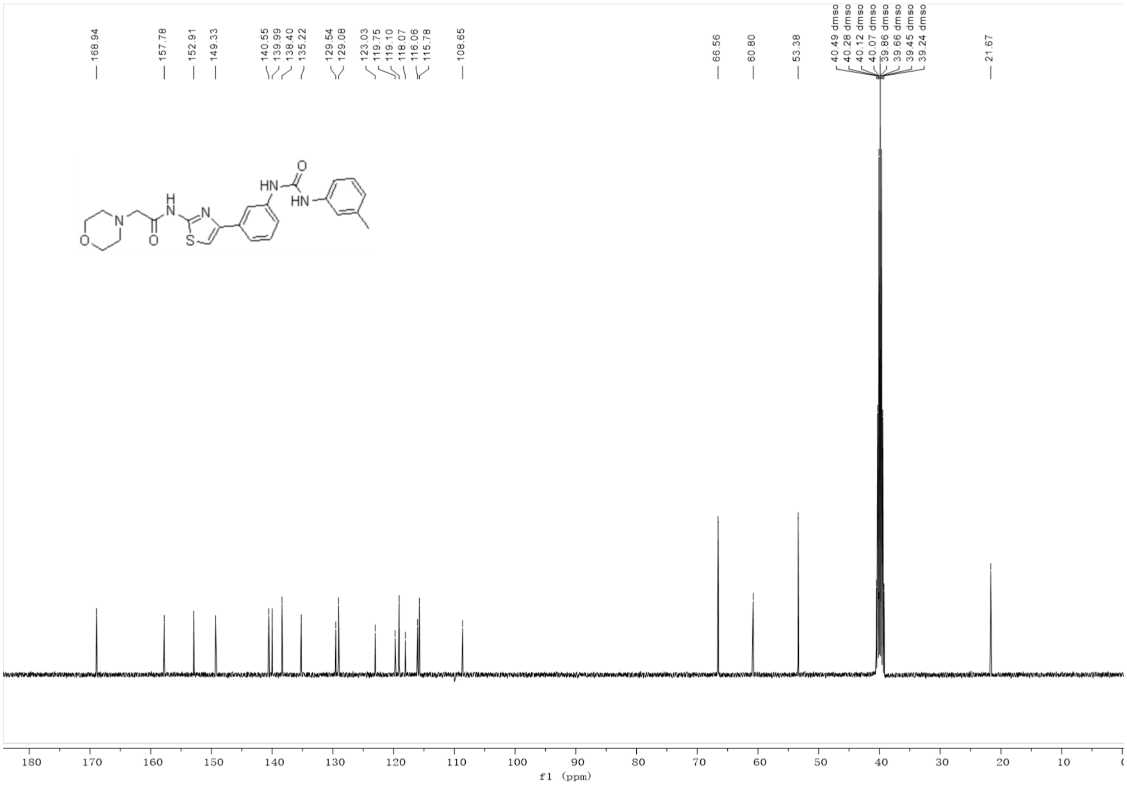

N-(4-(3-(3-(3-methoxyphenyl)ureido)phenyl)thiazol-2-yl)-2-morpholinoacetamide (**18**):

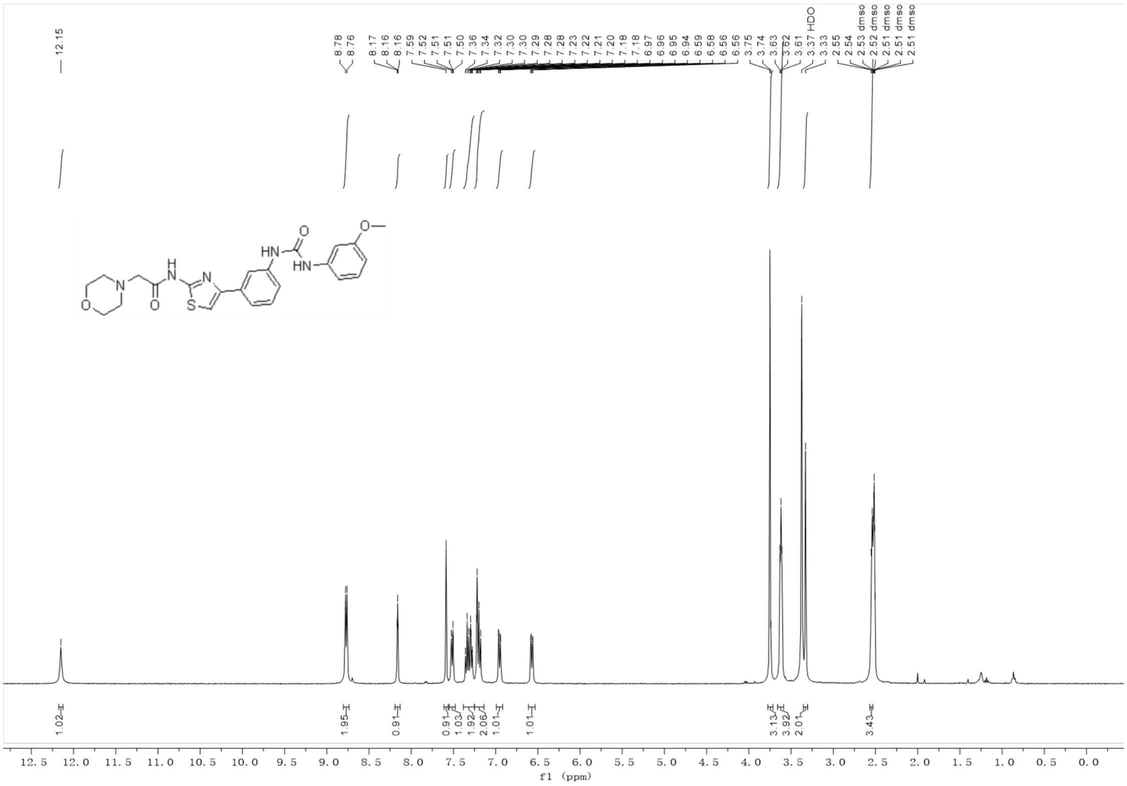

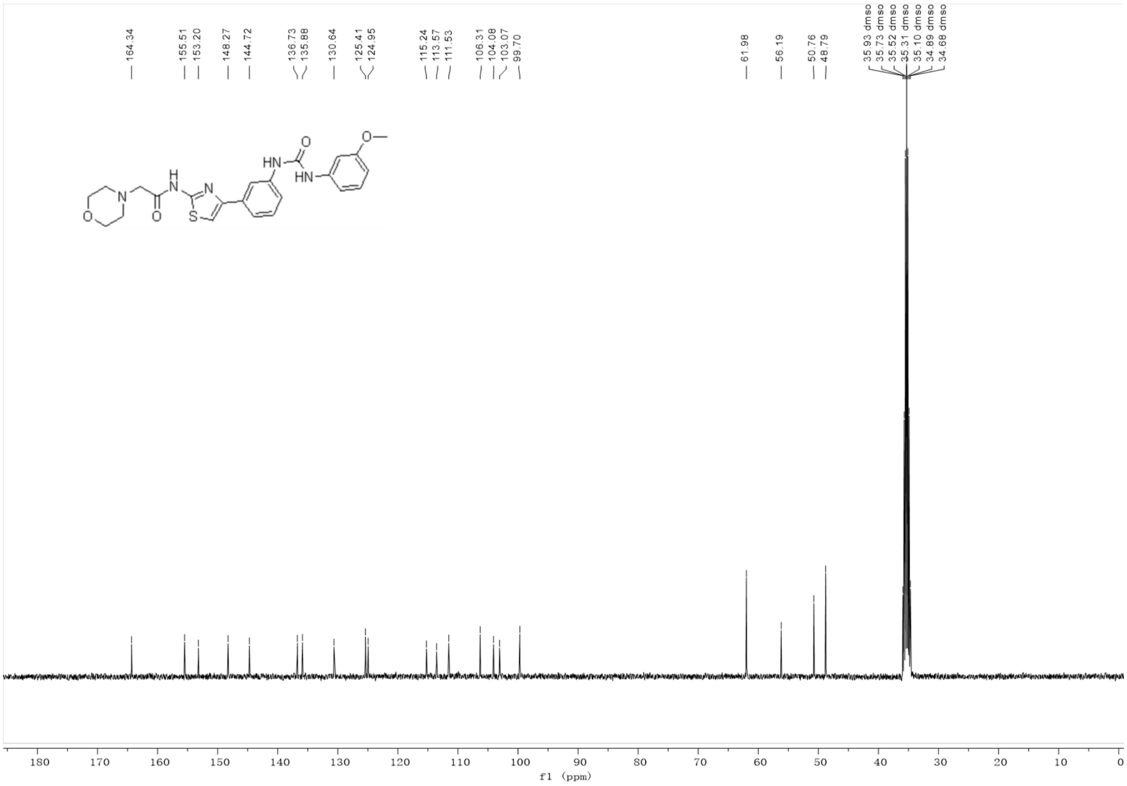

N-(4-(3-(3-(4-methoxyphenyl)ureido)phenyl)thiazol-2-yl)-2-morpholinoacetamide (**19**):

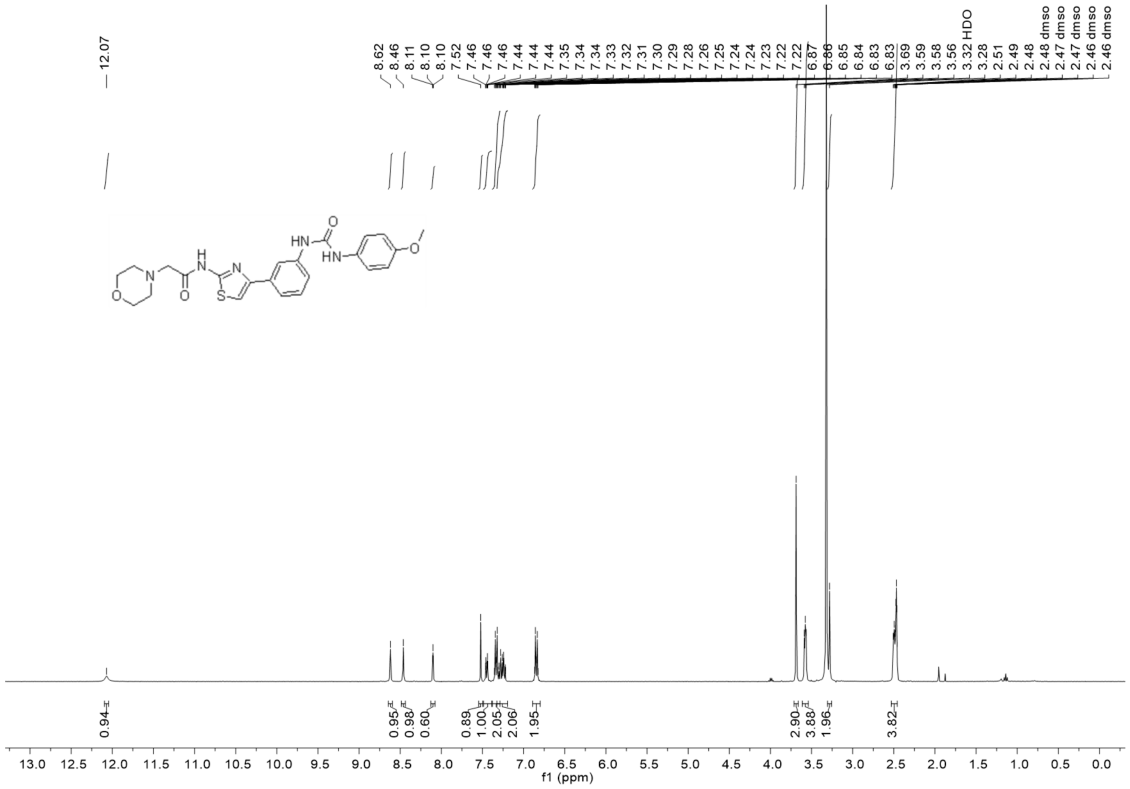

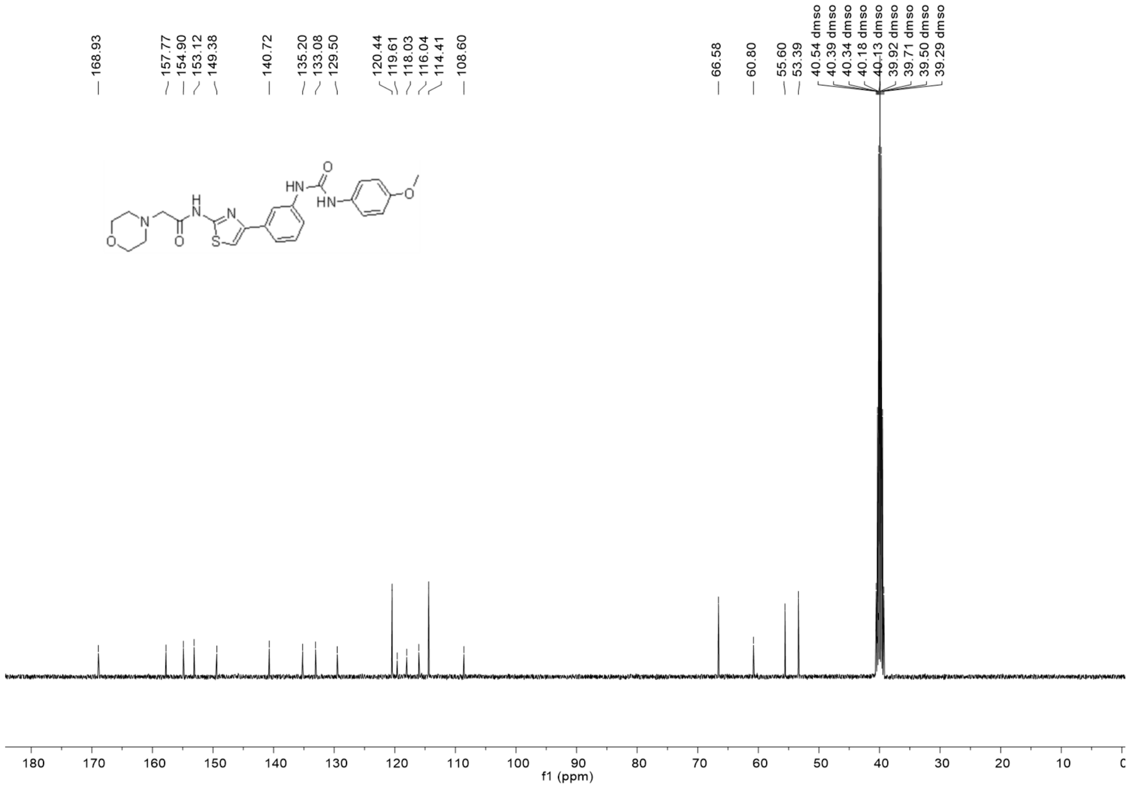

2-morpholino-*N*-(4-(3-(3-(4-(trifluoromethoxy)phenyl)ureido)phenyl)thiazol-2-yl)acetamide (**20**)

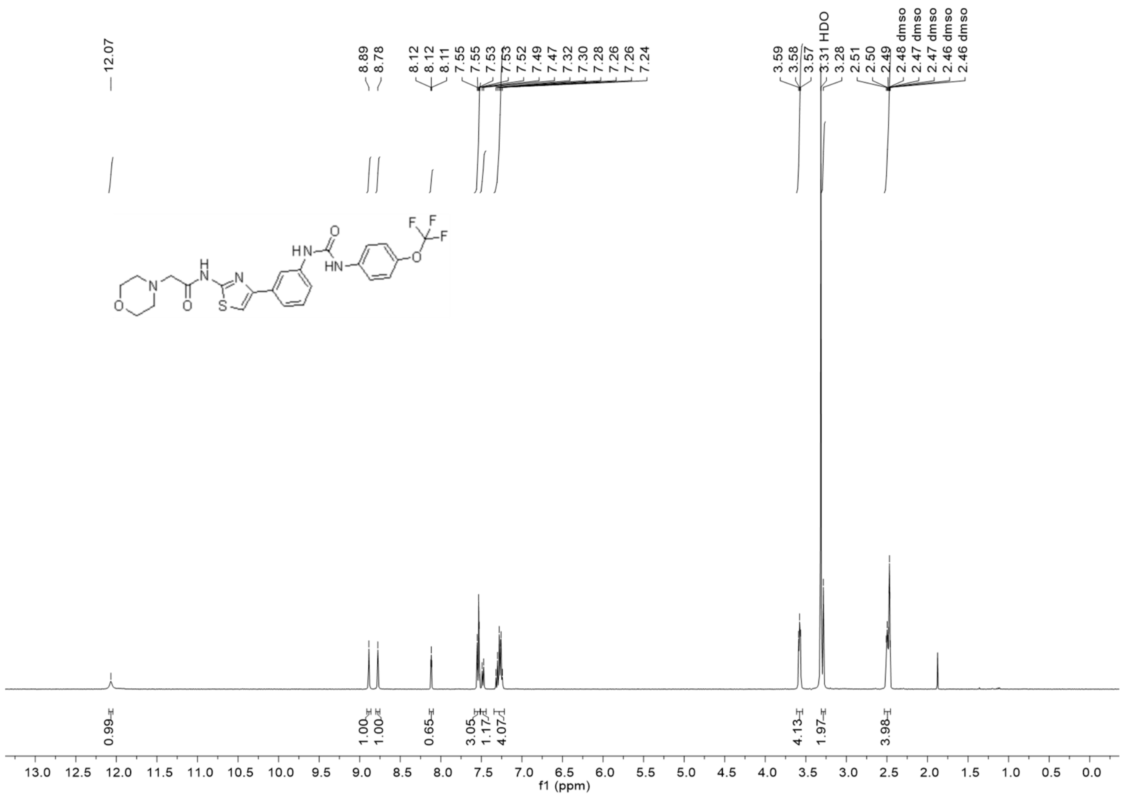

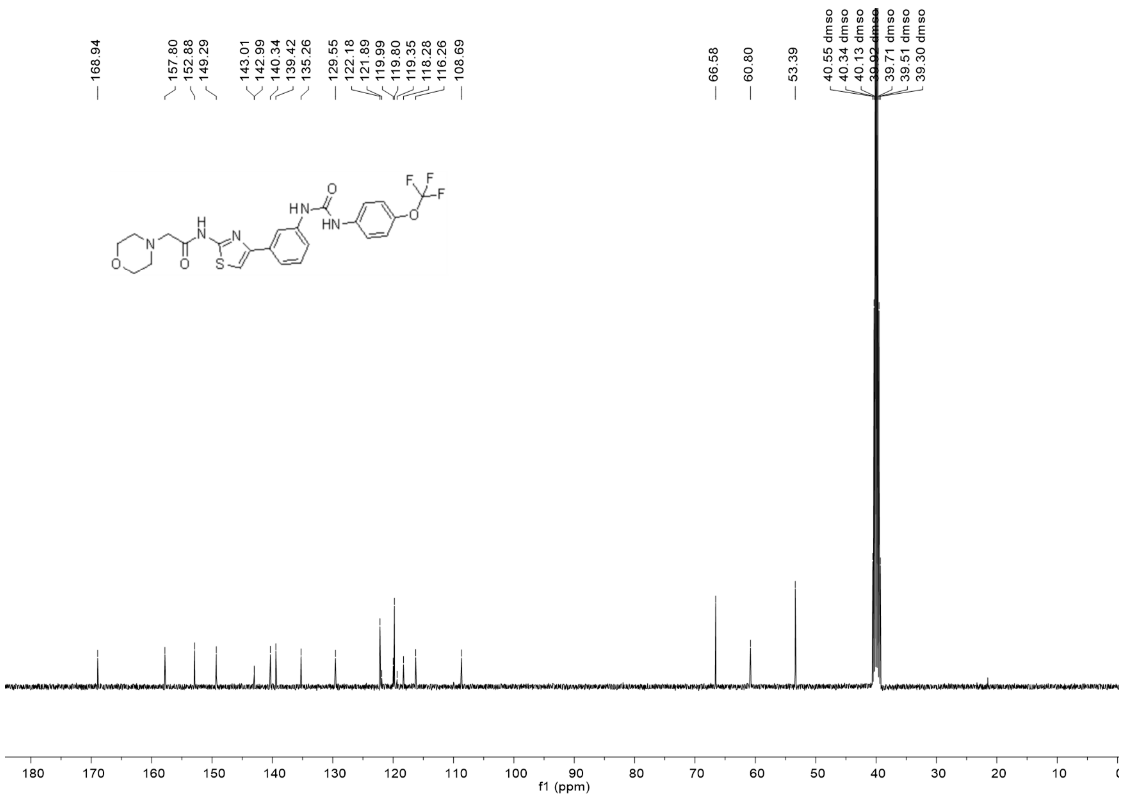

N-(4-(3-(3-benzylureido)phenyl)thiazol-2-yl)-2-morpholinoacetamide (**21**)

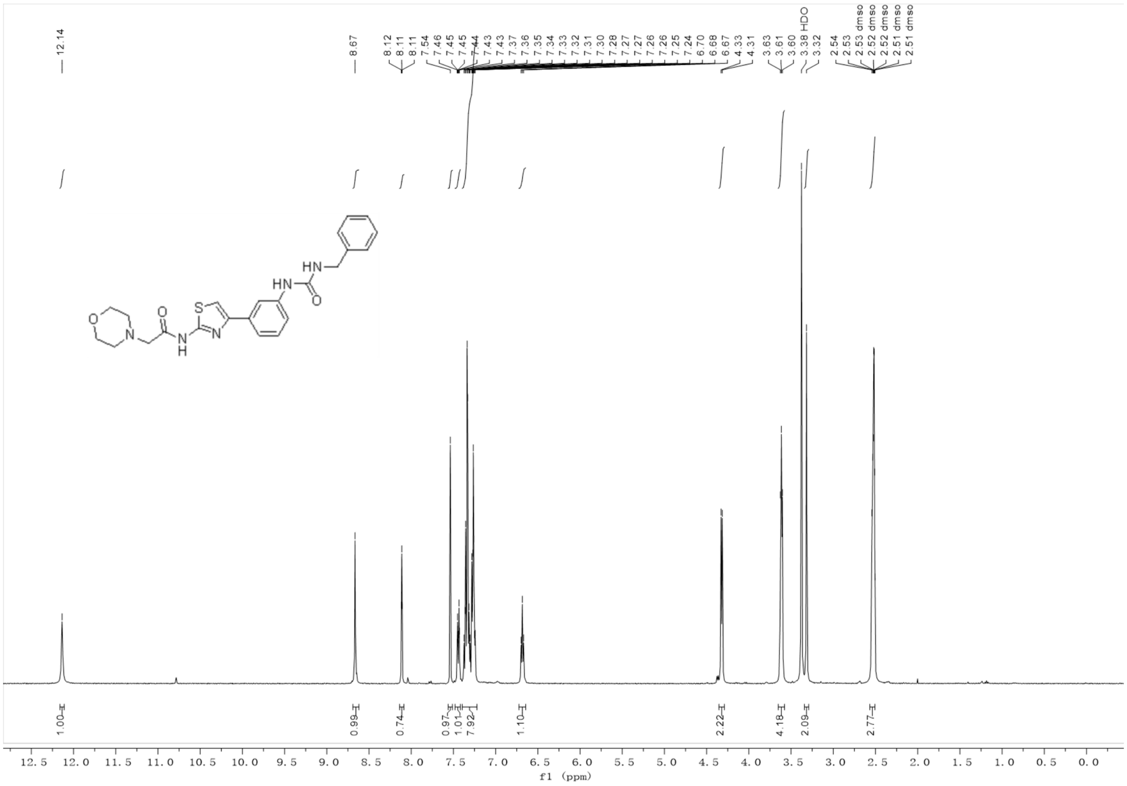

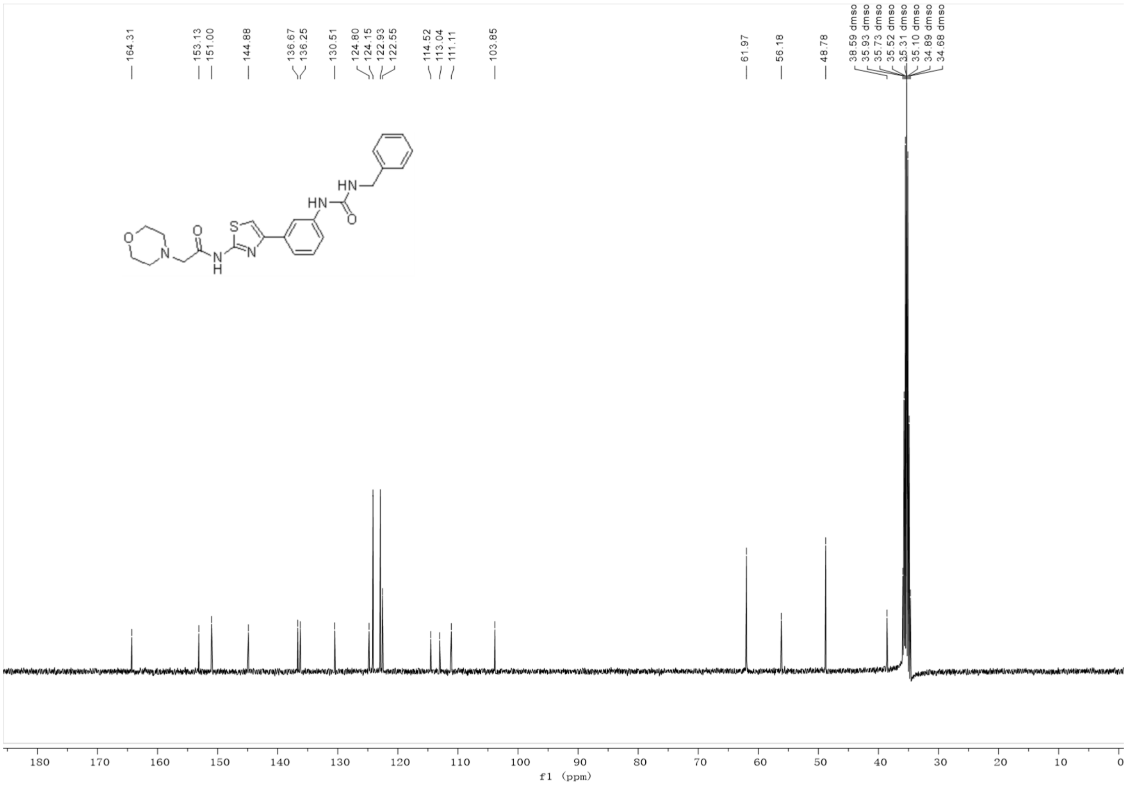

N-(4-(3-(3-(4-chlorophenyl)ureido)phenyl)thiazol-2-yl)-2-(piperidin-1-yl)acetamide (**22**):

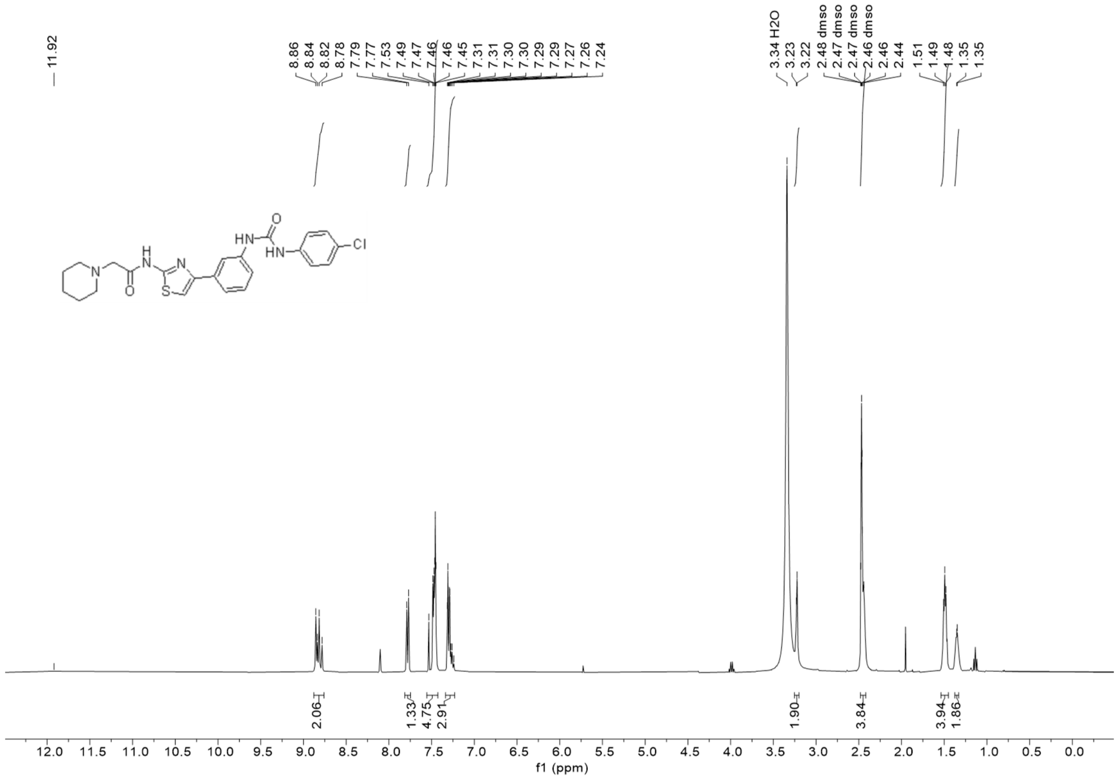

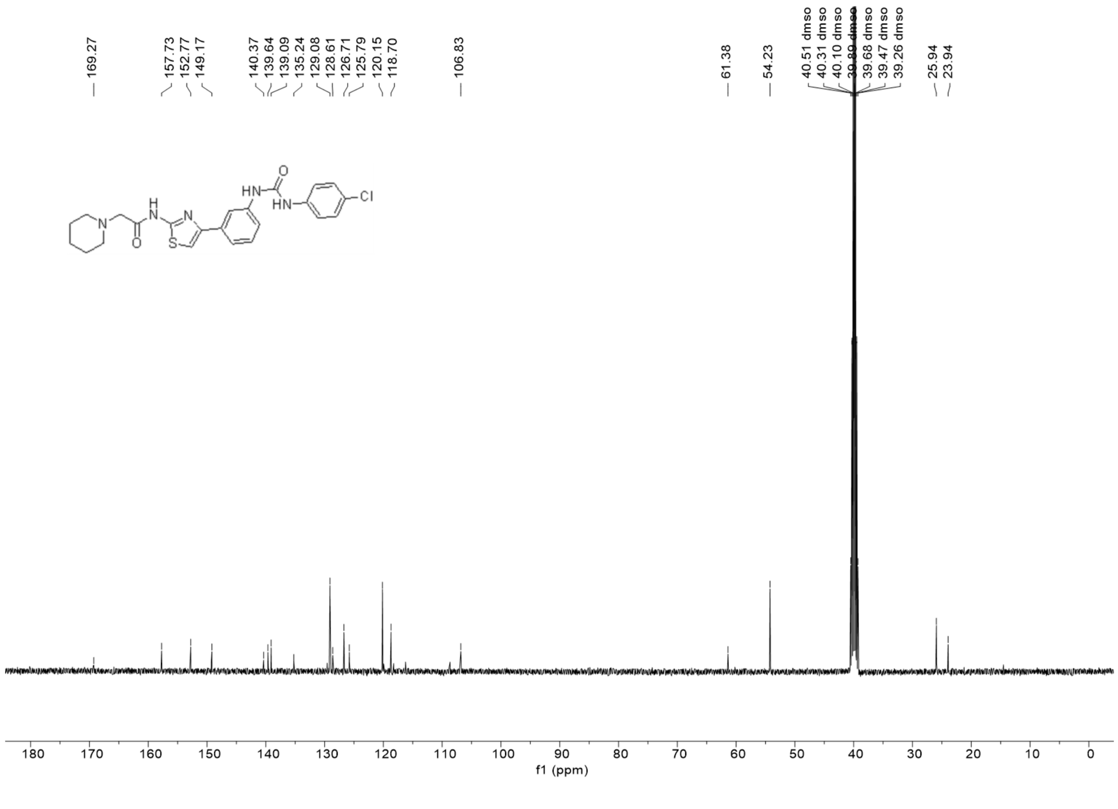

N-(4-(3-(3-(3-chlorophenyl)ureido)phenyl)thiazol-2-yl)-2-(piperidin-1-yl)acetamide (**23**):

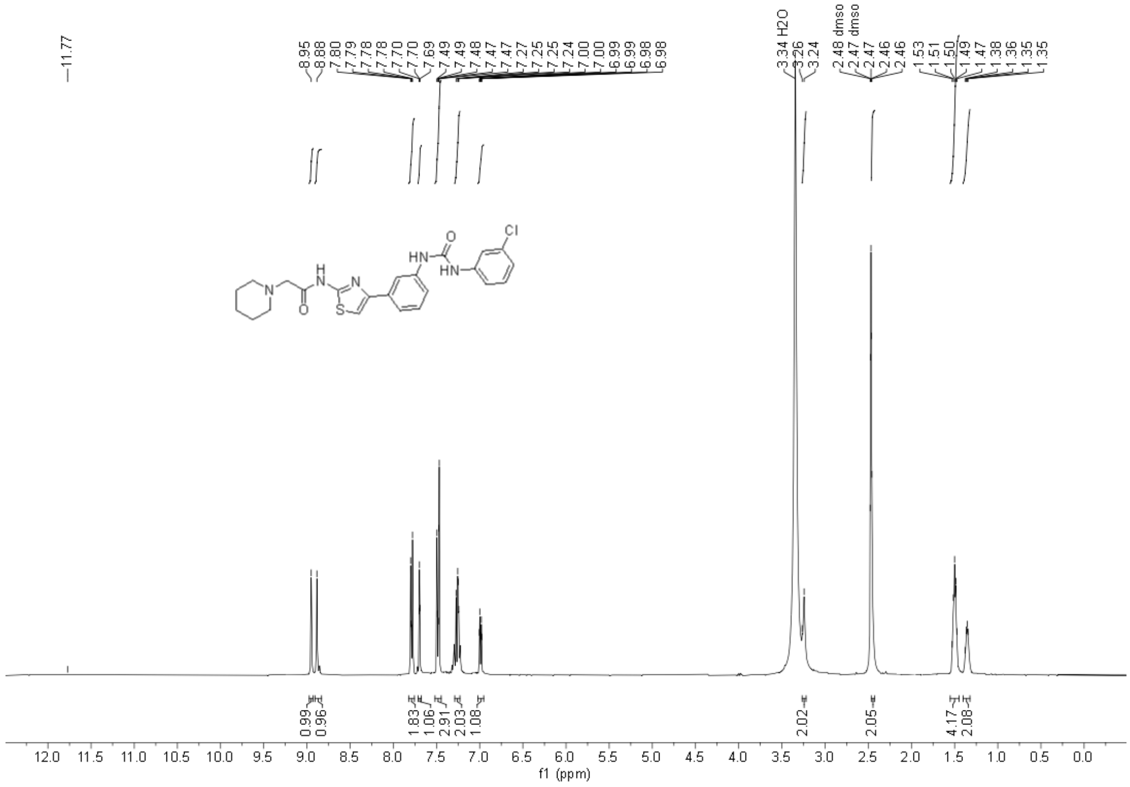

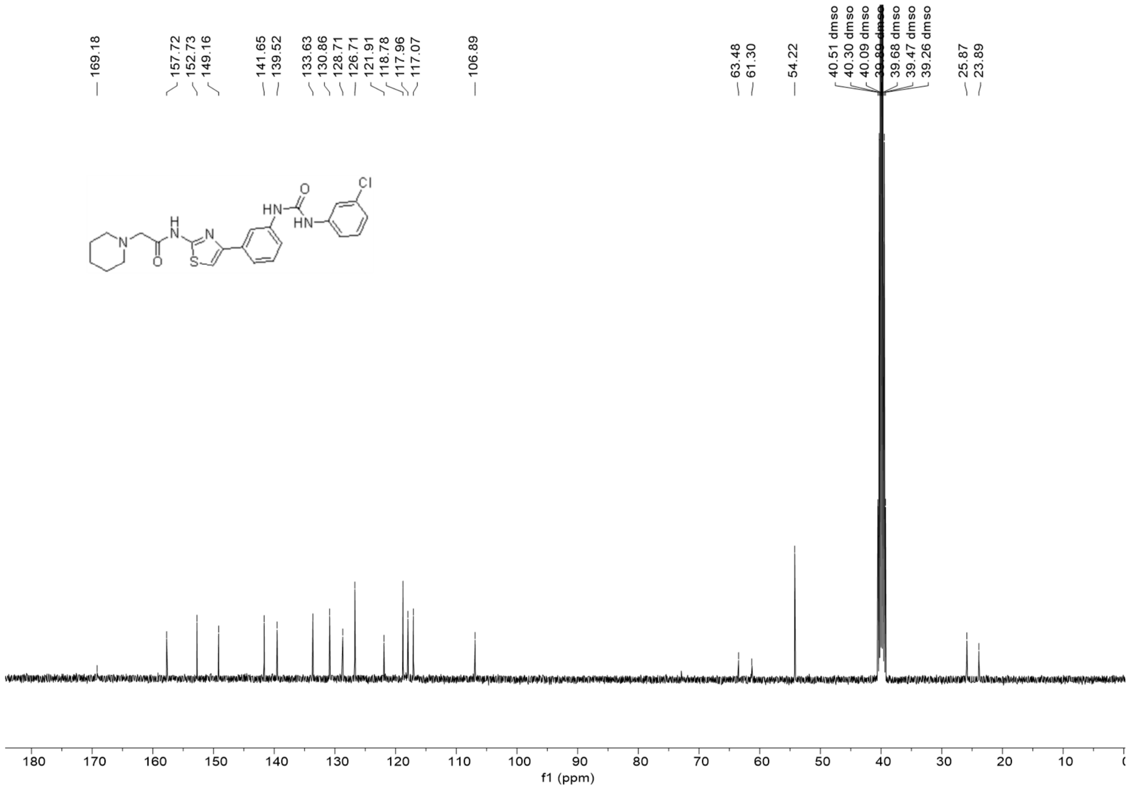

N-(4-(3-(3-(2,4-dichlorophenyl)ureido)phenyl)thiazol-2-yl)-2-(piperidin-1-yl)acetamide (**24**):

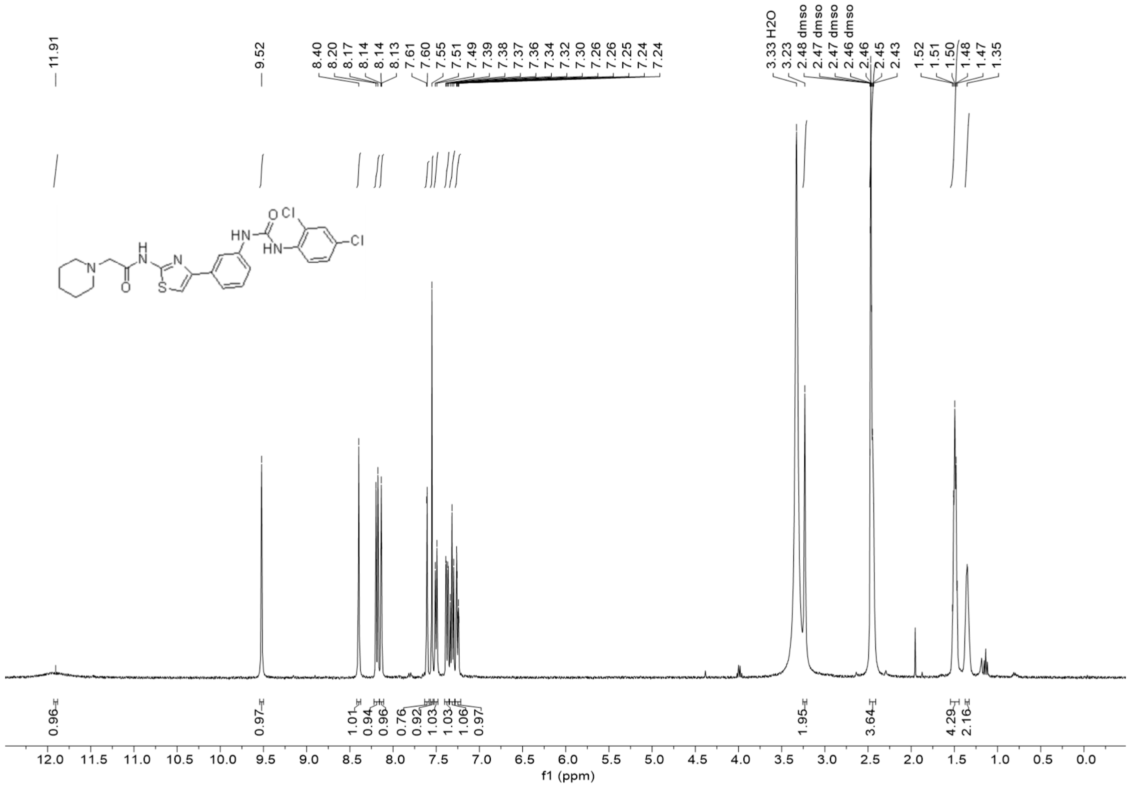

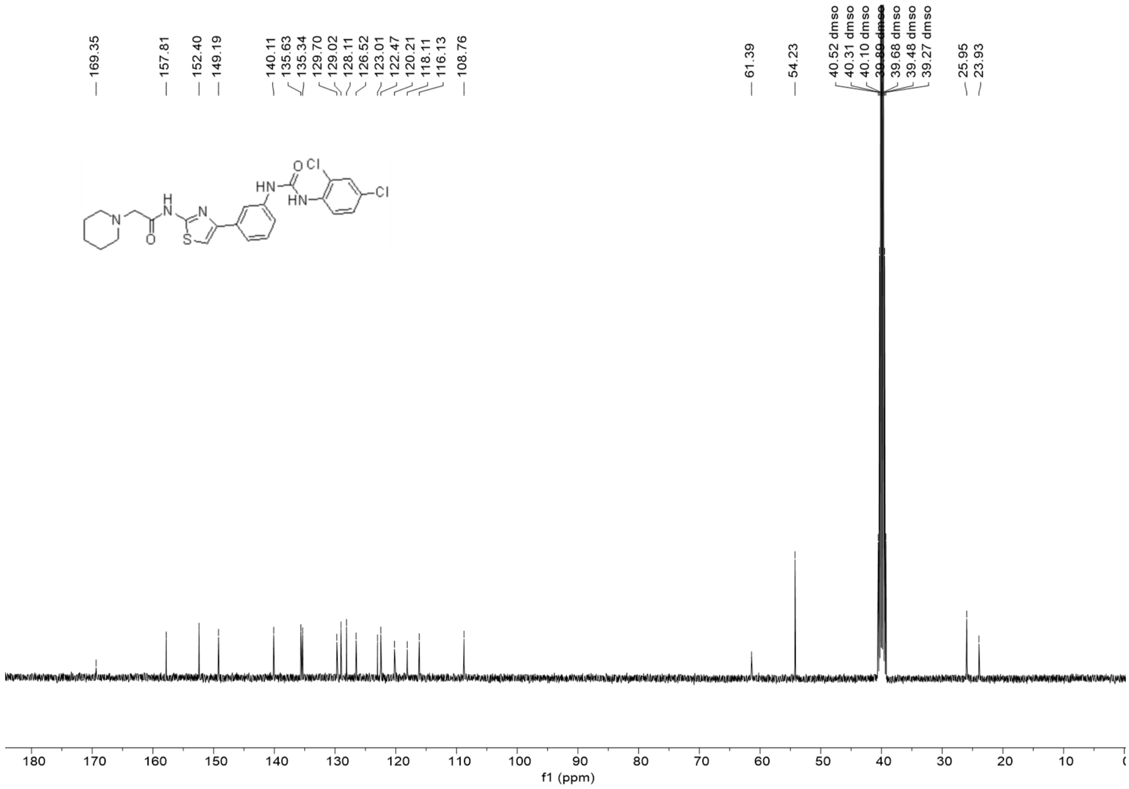

N-(4-(3-(3-(3,5-dichlorophenyl)ureido)phenyl)thiazol-2-yl)-2-(piperidin-1-yl)acetamide (**25**):

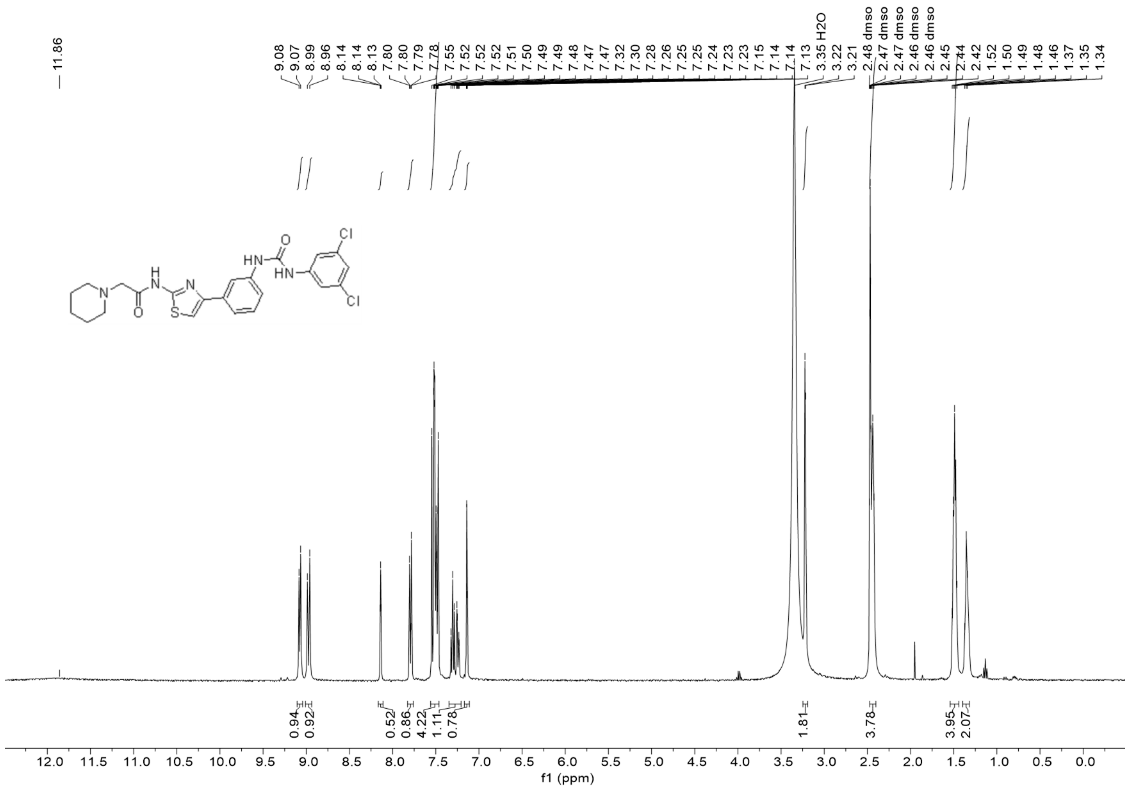

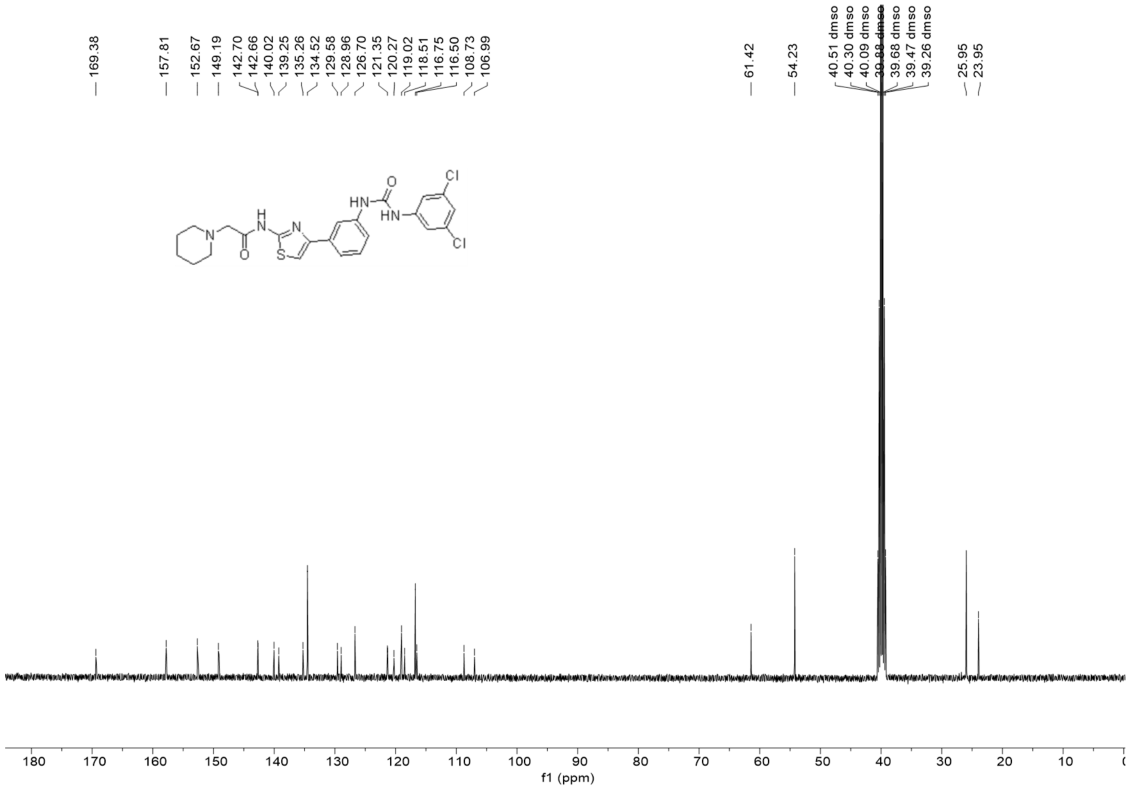

N-(4-(3-(3-(3-chloro-4-methylphenyl)ureido)phenyl)thiazol-2-yl)-2-(piperidin-1-yl)acetamide (**26**):

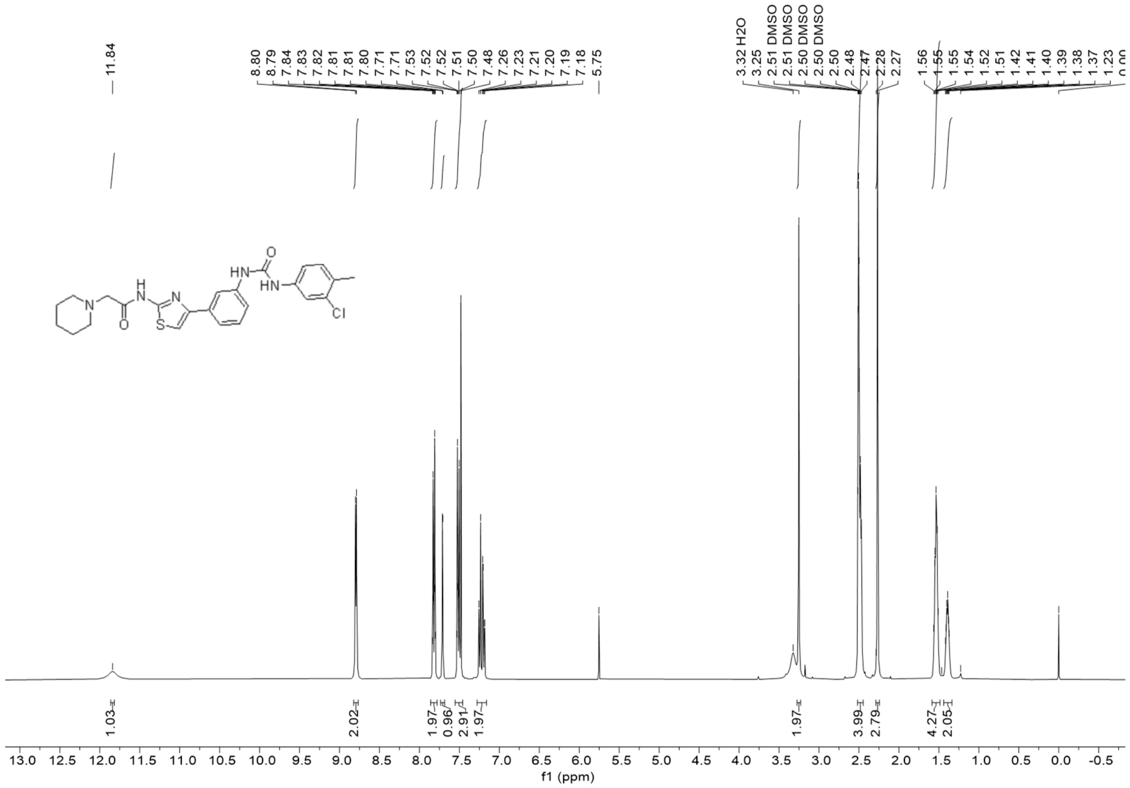

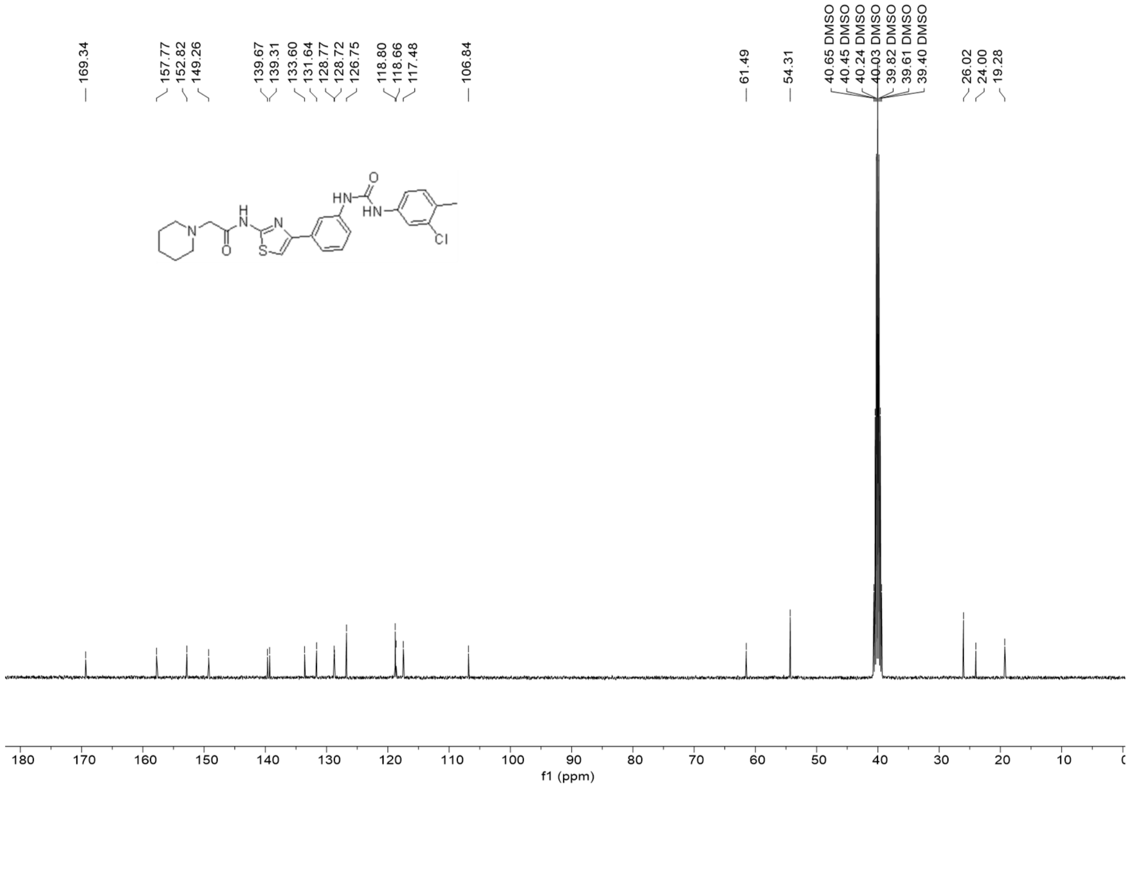

N-(4-(3-(3-(4-bromophenyl)ureido)phenyl)thiazol-2-yl)-2-(piperidin-1-yl)acetamide (**27**):

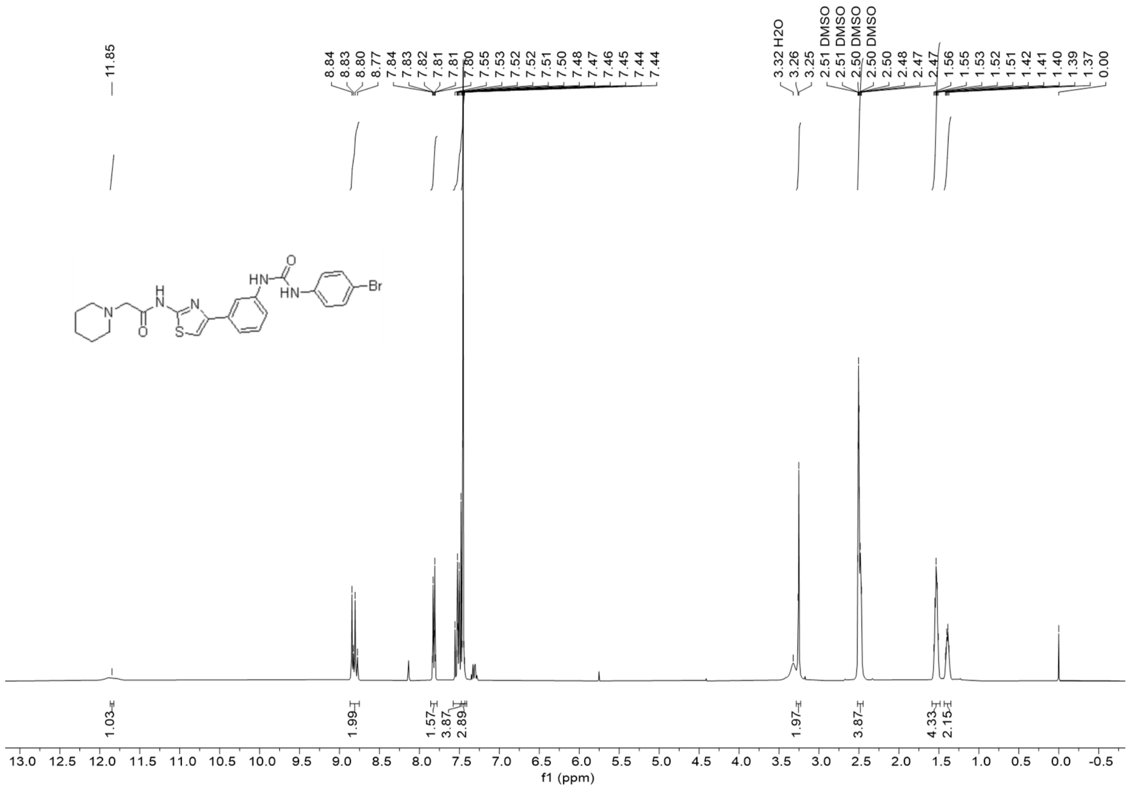

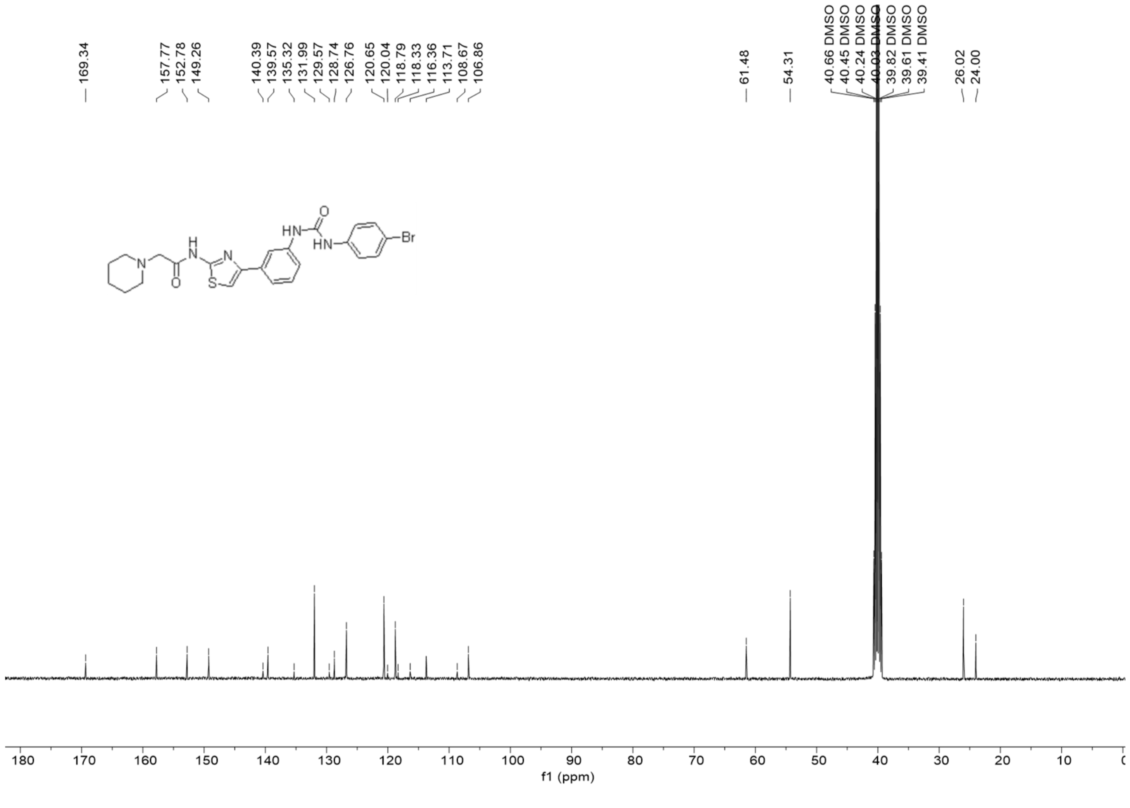

2-(piperidin-1-yl)-*N*-(4-(3-(3-(4-(trifluoromethyl)phenyl)ureido)phenyl)thiazol-2-yl)acetamide (**28**):

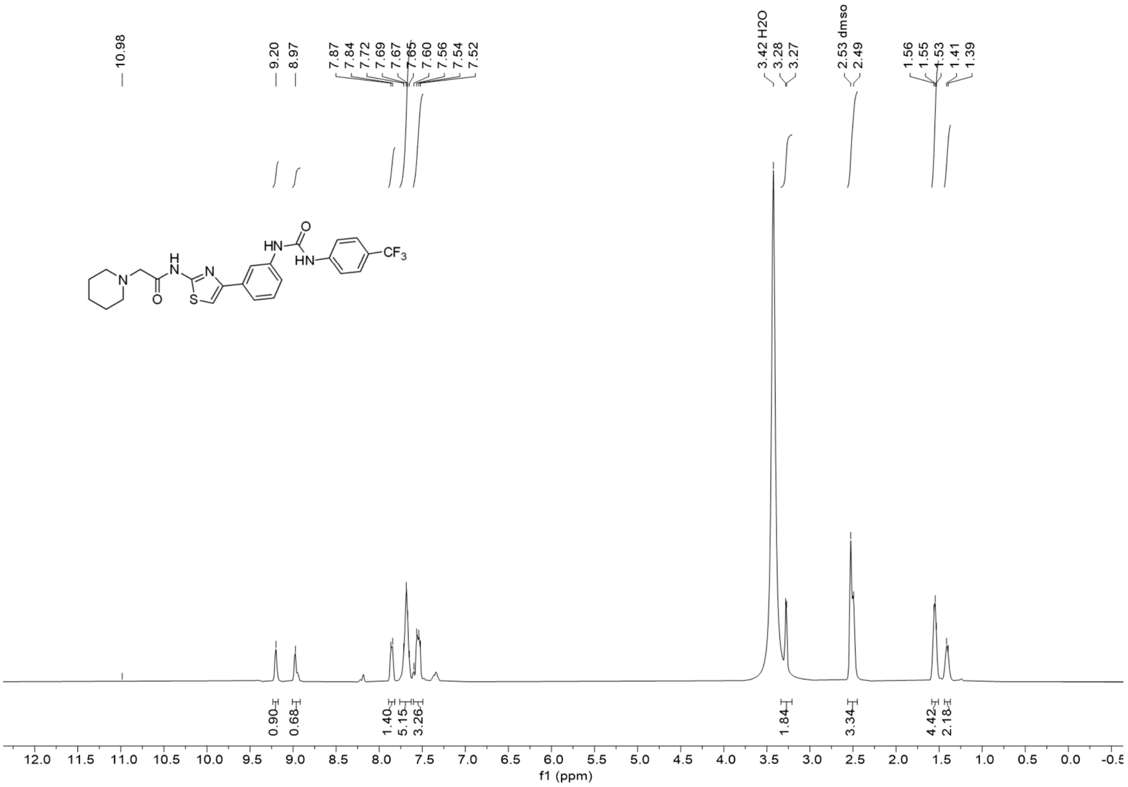

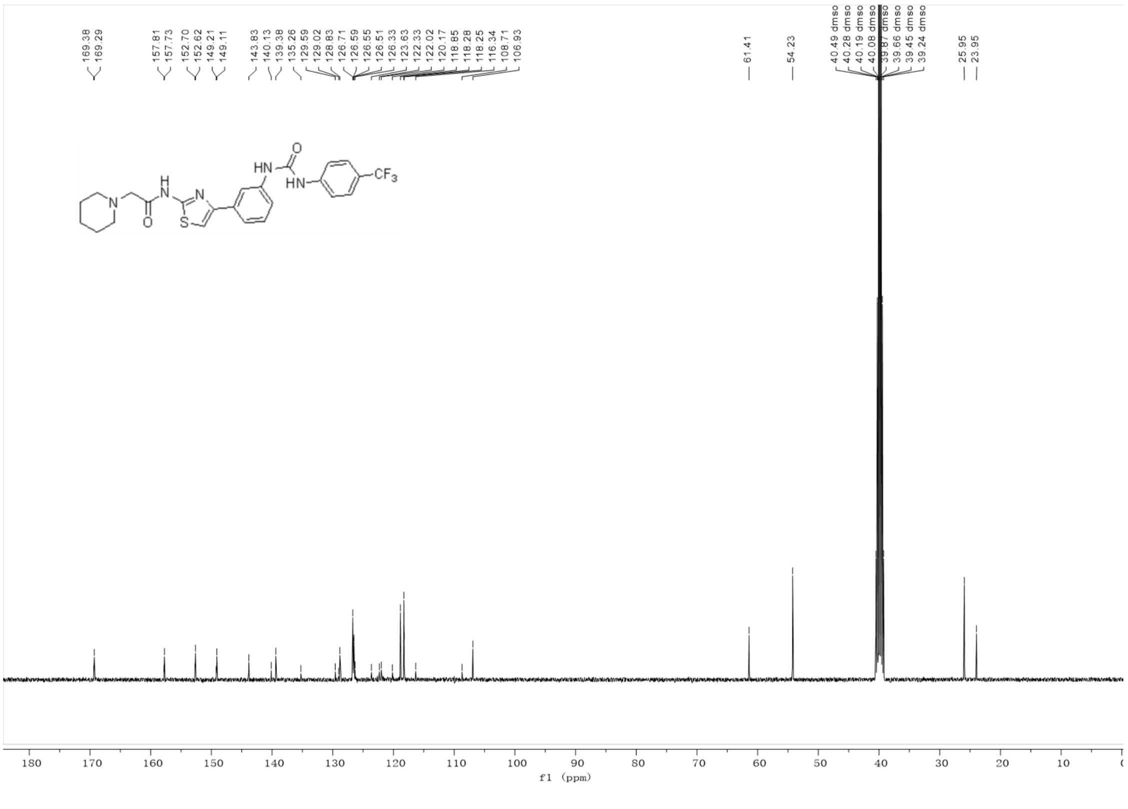

N-(4-(3-(3-(4-chloro-3-(trifluoromethyl)phenyl)ureido)phenyl)thiazol-2-yl)-2-(piperidin-1-yl)acetamide (**29**)

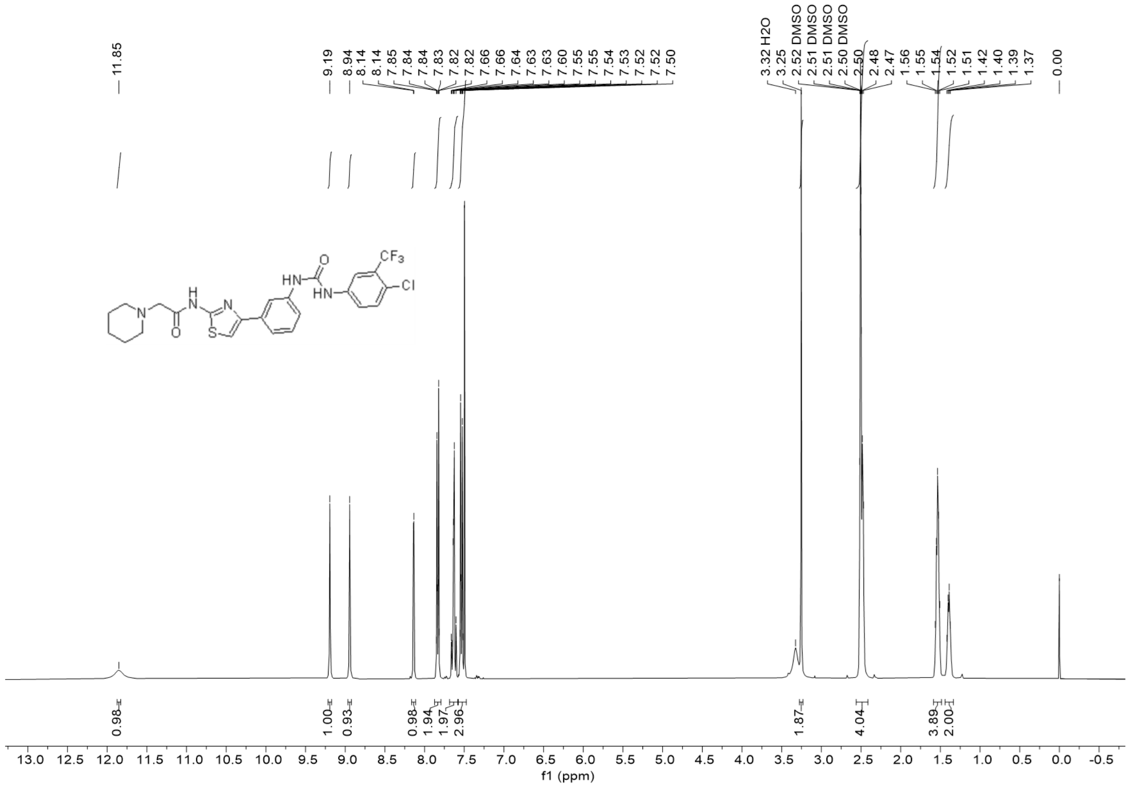

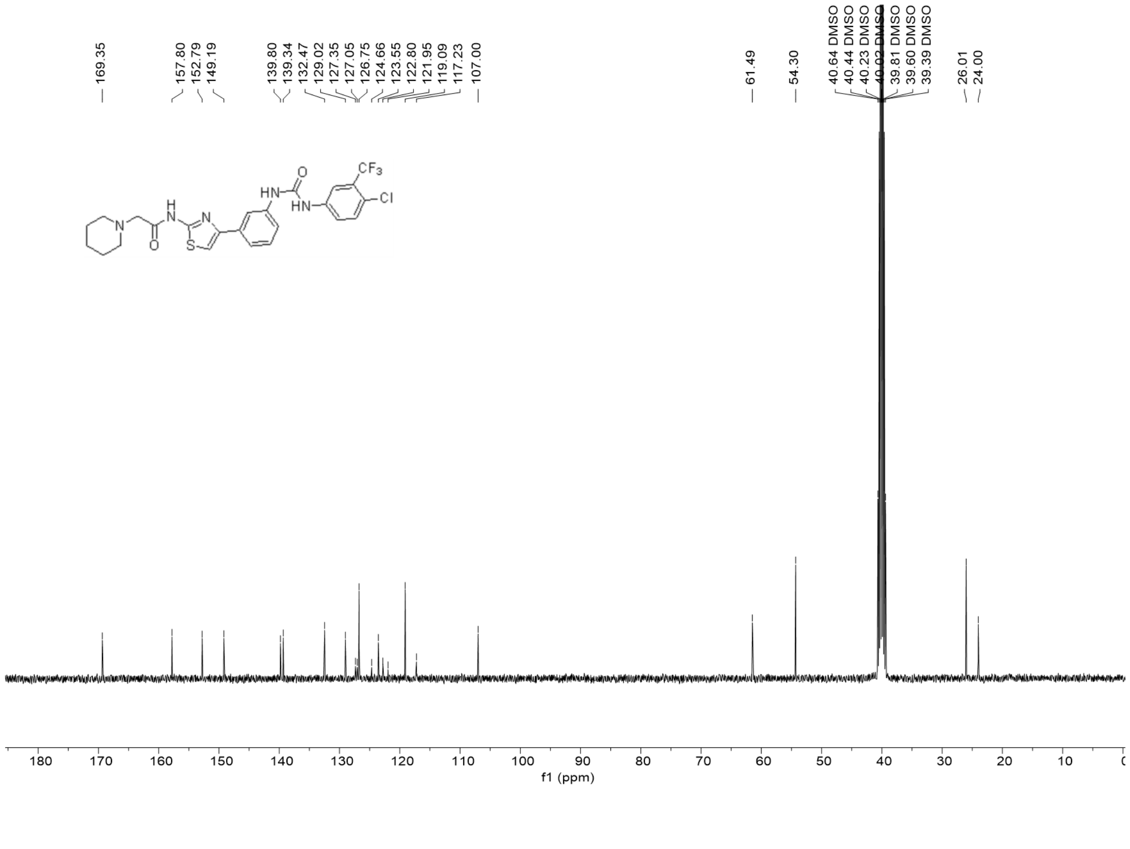

N-(4-(3-(3-(3-methoxyphenyl)ureido)phenyl)thiazol-2-yl)-2-(piperidin-1-yl)acetamide (**30**):

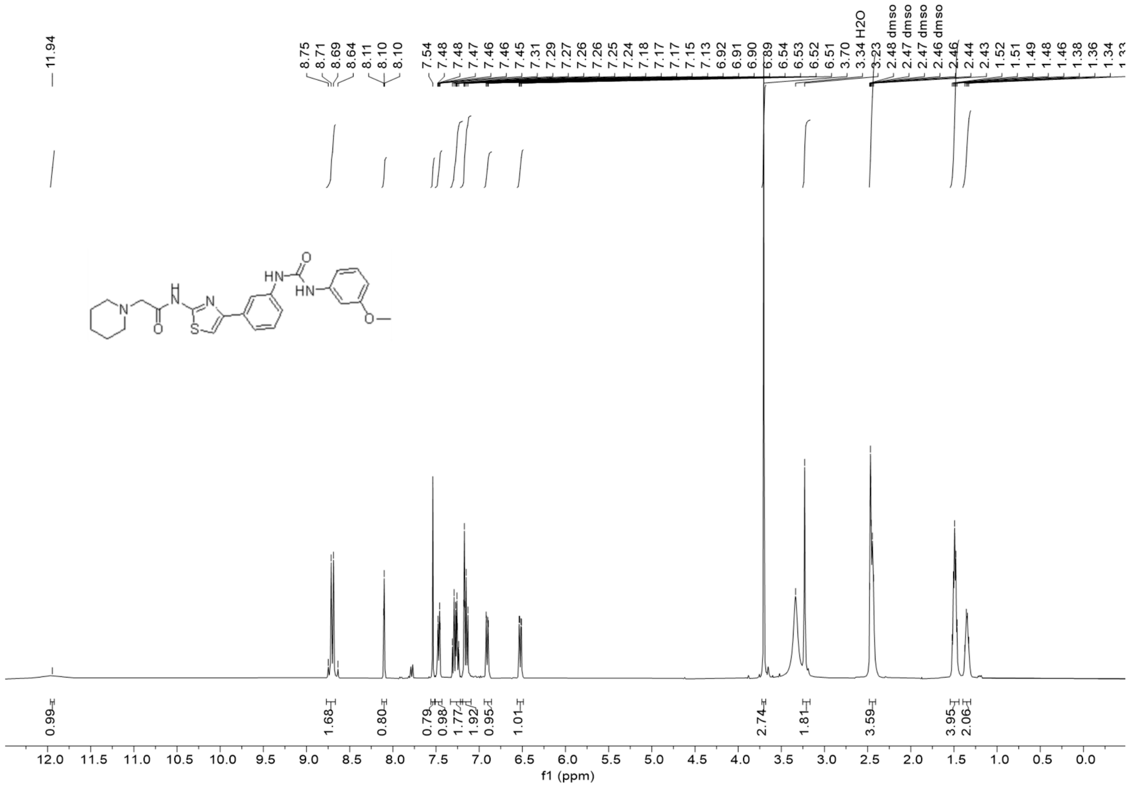

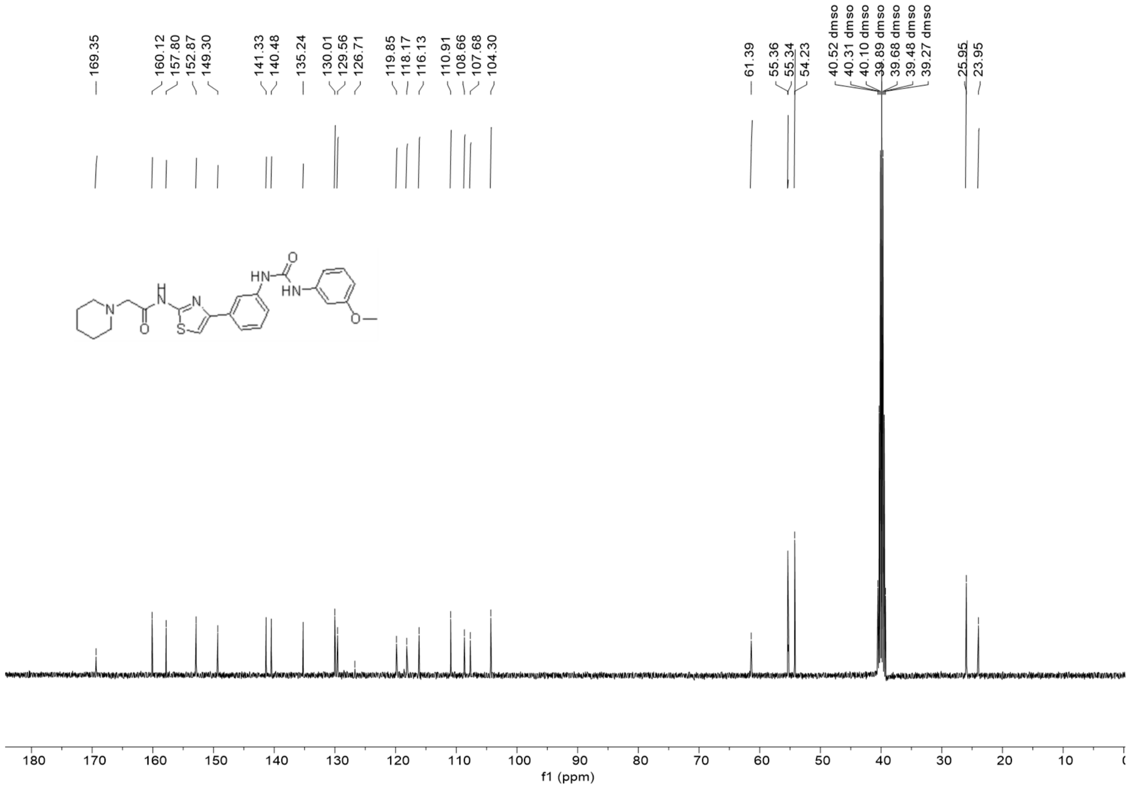

HRMS of compound (5~30):

4-(3-nitrophenyl)thiazol-2-amine (**1**):

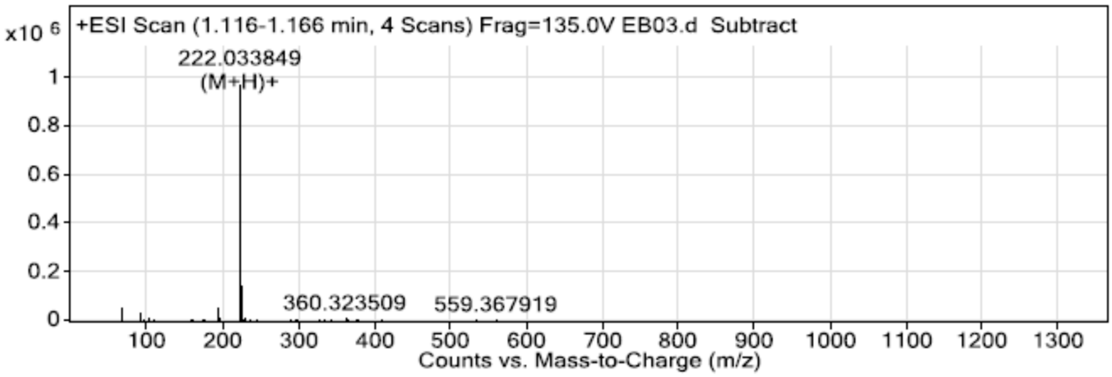

2-chloro-*N*-(4-(3-nitrophenyl)thiazol-2-yl)acetamide (**2**):

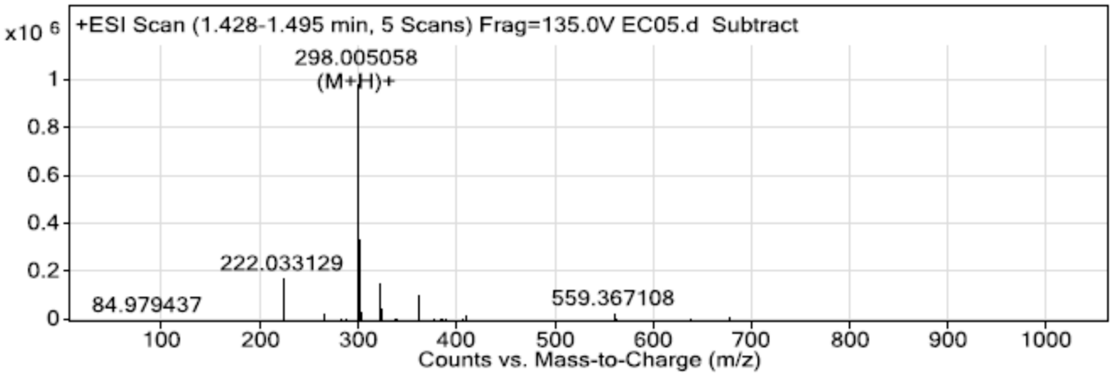

2-morpholino-*N*-(4-(3-nitrophenyl)thiazol-2-yl)acetamide (**3a**):

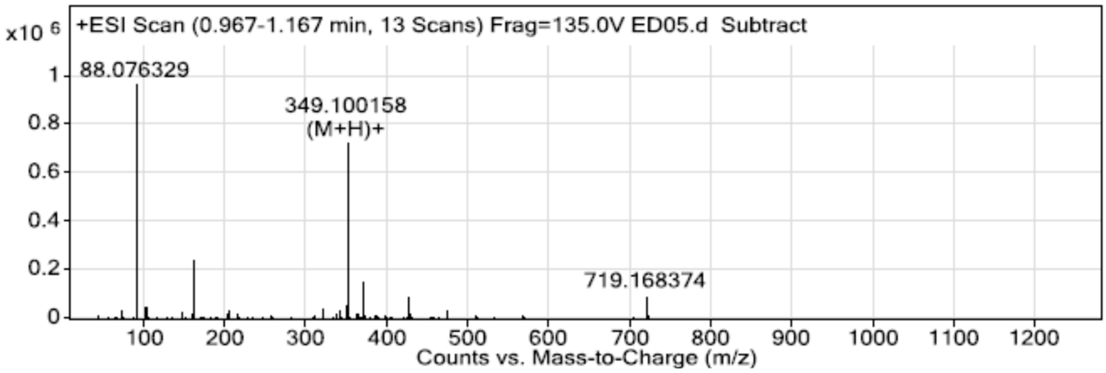

N-(4-(3-nitrophenyl)thiazol-2-yl)-2-(piperidin-1-yl)acetamide (**3b**):

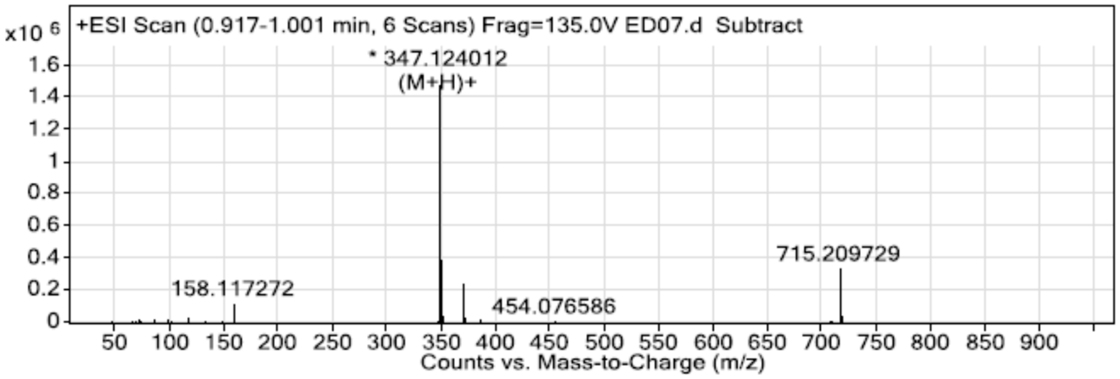

N-(4-(3-aminophenyl)thiazol-2-yl)-2-morpholinoacetamide (**4a**):

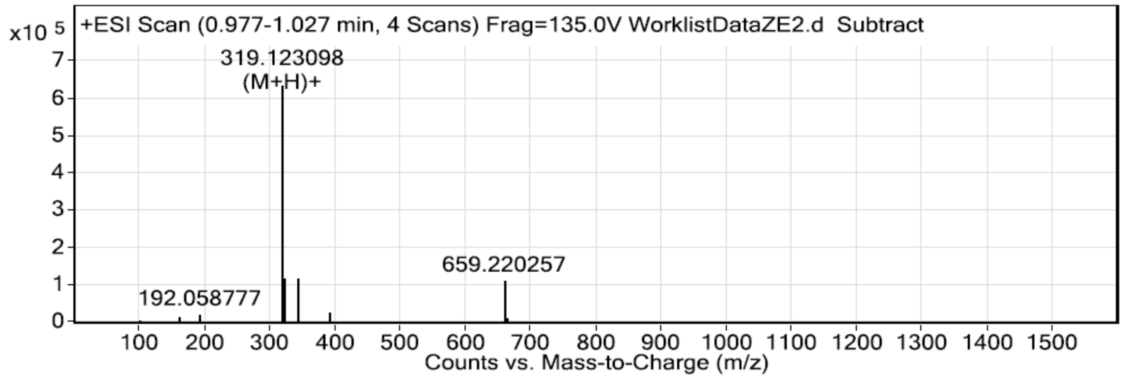

N-(4-(3-aminophenyl)thiazol-2-yl)-2-(piperidin-1-yl)acetamide (**4b**):

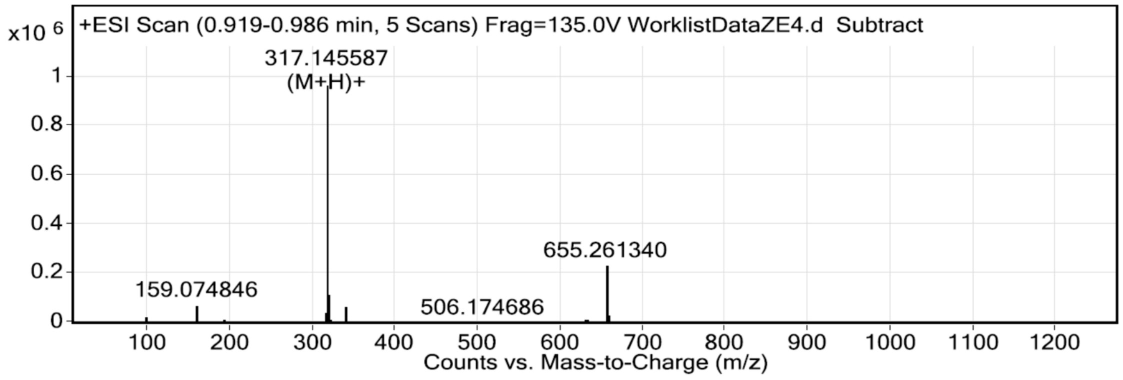

N-(4-(3-(3-cyclohexylureido)phenyl)thiazol-2-yl)-2-morpholinoacetamide (**5**):

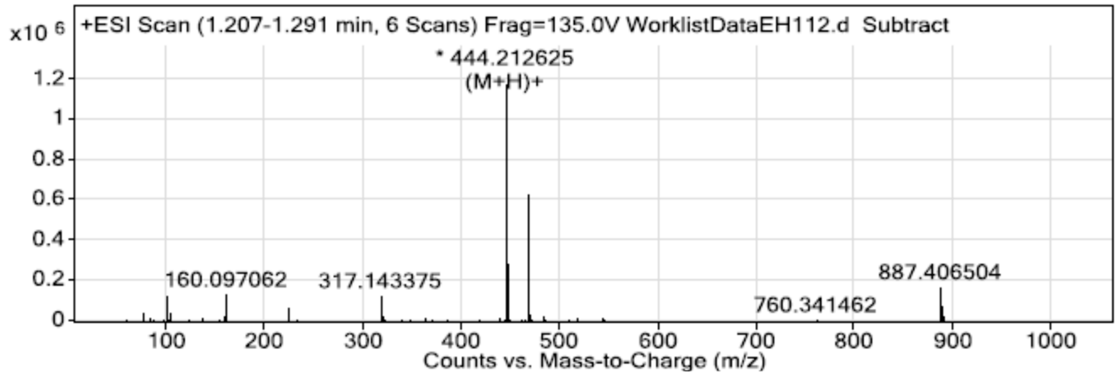

N-(4-(3-(3-(4-chlorophenyl)ureido)phenyl)thiazol-2-yl)-2-morpholinoacetamide (**6**):

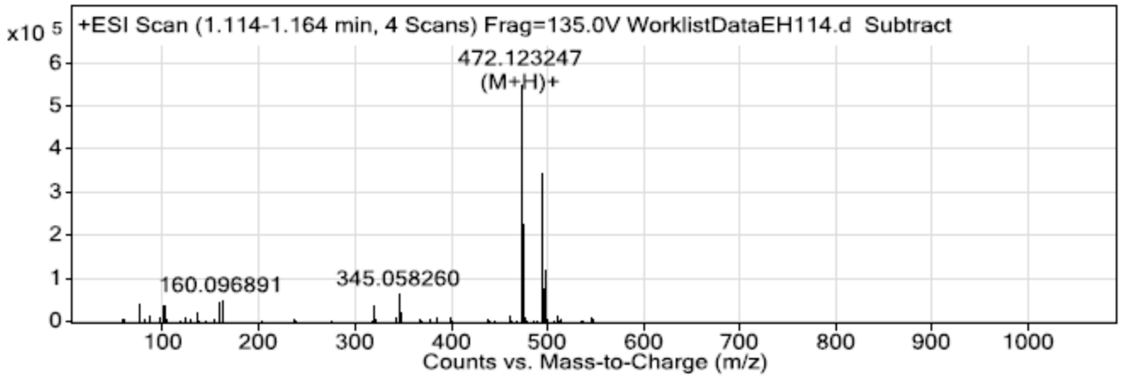

N-(4-(3-(3-(3-chlorophenyl)ureido)phenyl)thiazol-2-yl)-2-morpholinoacetamide (**7**):

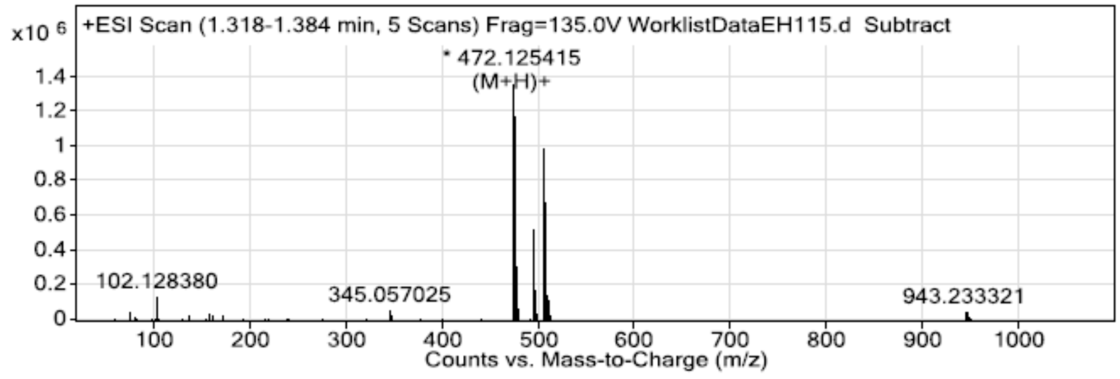

N-(4-(3-(3-(2,4-dichlorophenyl)ureido)phenyl)thiazol-2-yl)-2-morpholin-4-ylacetamide (**8**)

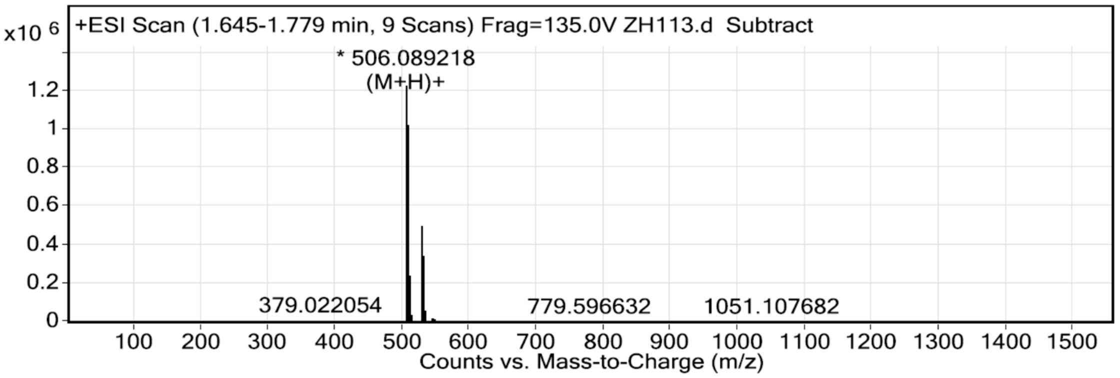

N-(4-(3-(3-(3,4-dichlorophenyl)ureido)phenyl)thiazol-2-yl)-2-morpholinoacetamide (**9**):

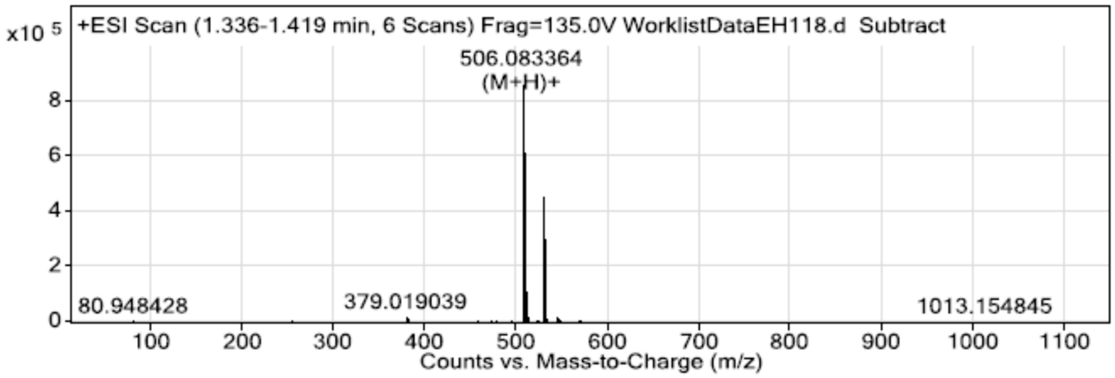

N-(4-(3-(3-(3,5-dichlorophenyl)ureido)phenyl)thiazol-2-yl)-2-morpholinoacetamide (**10**):

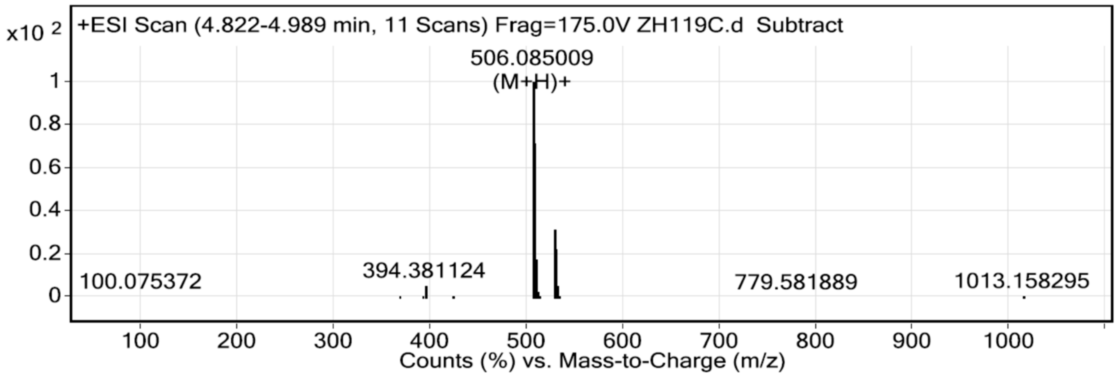

N-(4-(3-(3-(3-chloro-4-methylphenyl)ureido)phenyl)thiazol-2-yl)-2-morpholinoacetamide (**11**):

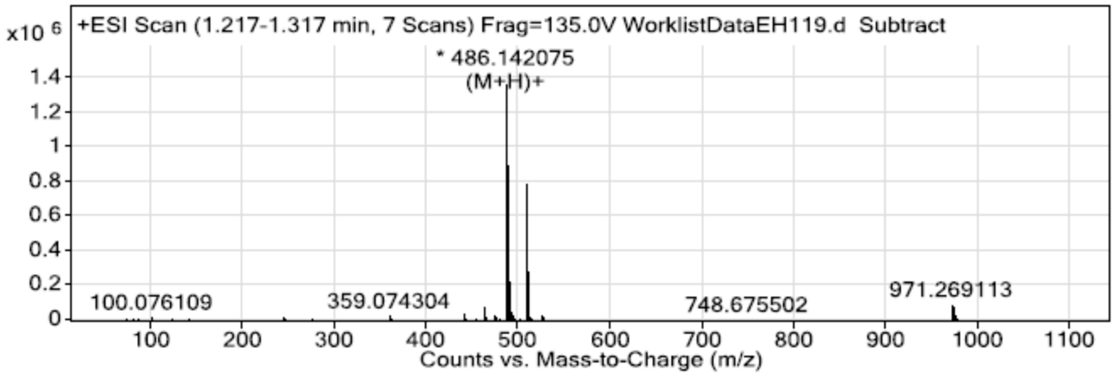

N-(4-(3-(3-(4-chloro-3-(trifluoromethyl)phenyl)ureido)phenyl)thiazol-2-yl)-2-morpholinoacetamide (**12**):

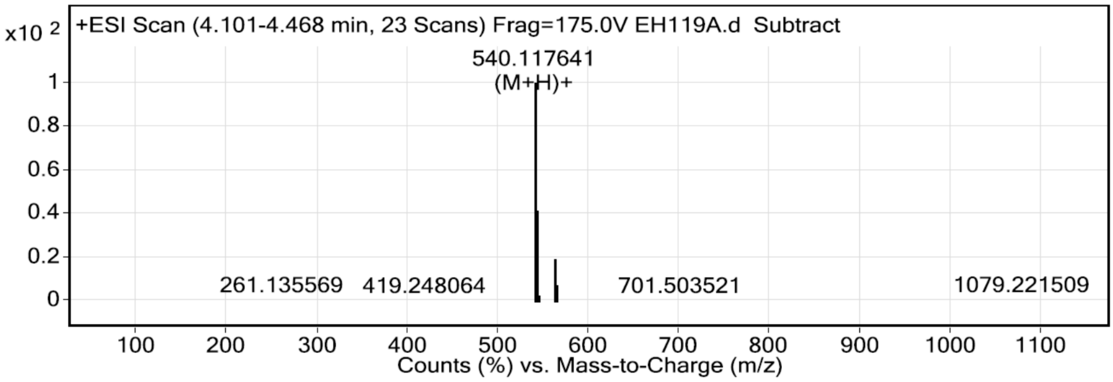

N-(4-(3-(3-(4-bromophenyl)ureido)phenyl)thiazol-2-yl)-2-morpholinoacetamide (**13**):

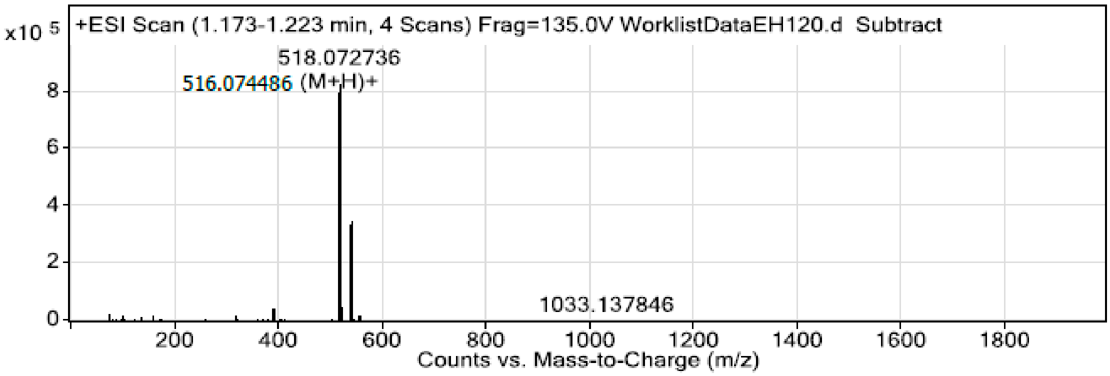

N-(4-(3-(3-(4-fluorophenyl)ureido)phenyl)thiazol-2-yl)-2-morpholinoacetamide (**14**):

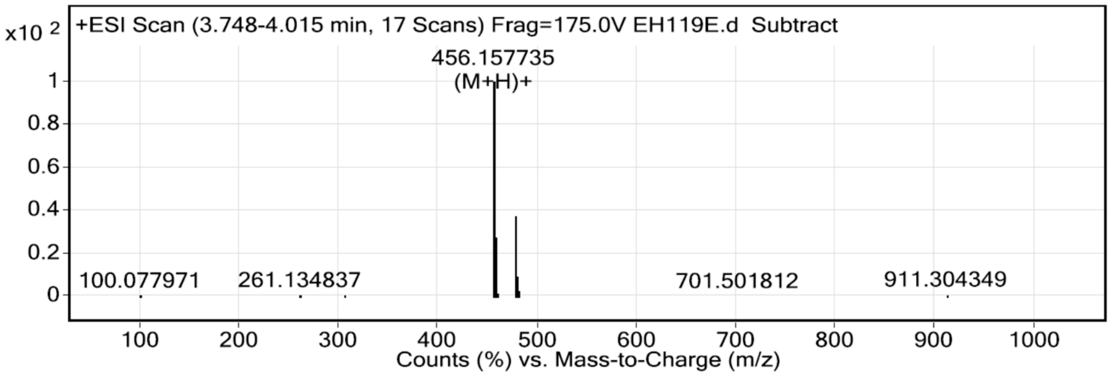

N-(4-(3-(3-(2,4-difluorophenyl)ureido)phenyl)thiazol-2-yl)-2-morpholinoacetamide (**15**):

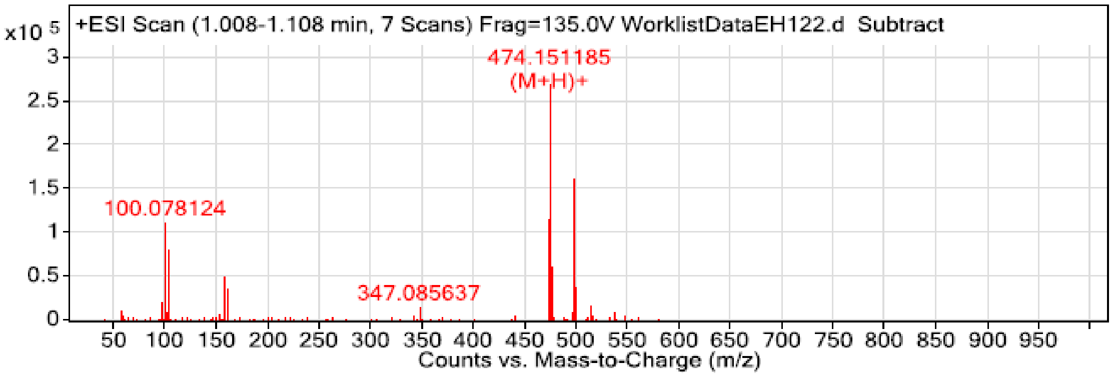

2-morpholino-*N*-(4-(3-(3-(4-(trifluoromethyl)phenyl)ureido)phenyl)thiazol-2-yl)acetamide (**16**):

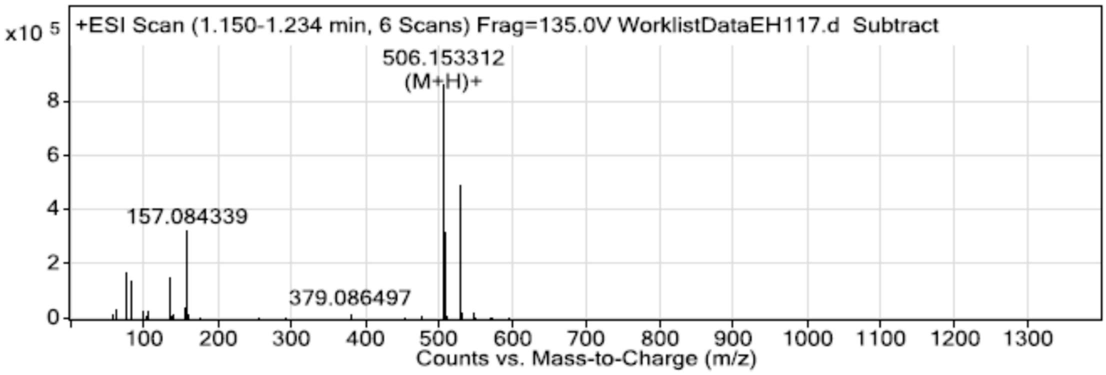

2-morpholino-*N*-(4-(3-(3-(m-tolyl)ureido)phenyl)thiazol-2-yl)acetamide (**17**):

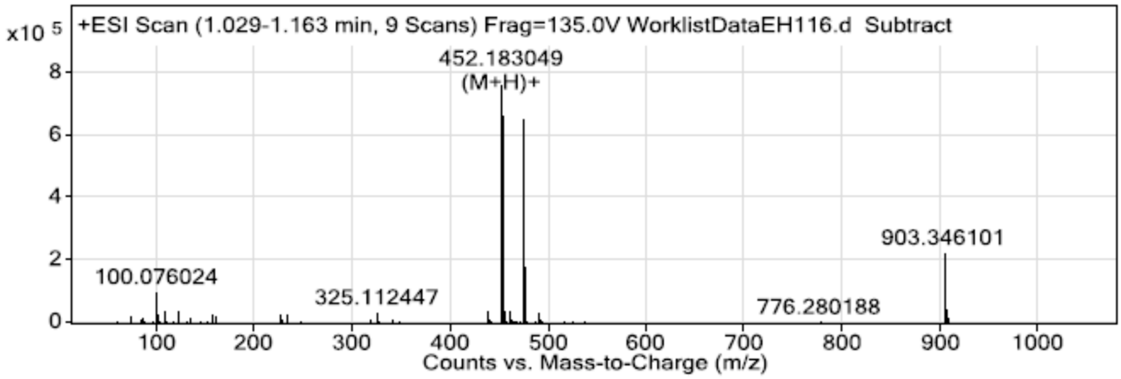

N-(4-(3-(3-(3-methoxyphenyl)ureido)phenyl)thiazol-2-yl)-2-morpholinoacetamide (**18**):

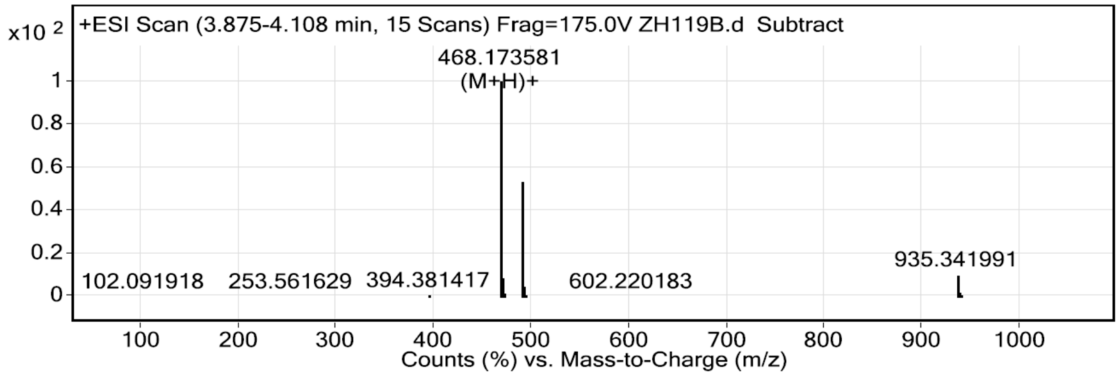

N-(4-(3-(3-(4-methoxyphenyl)ureido)phenyl)thiazol-2-yl)-2-morpholinoacetamide (**19**):

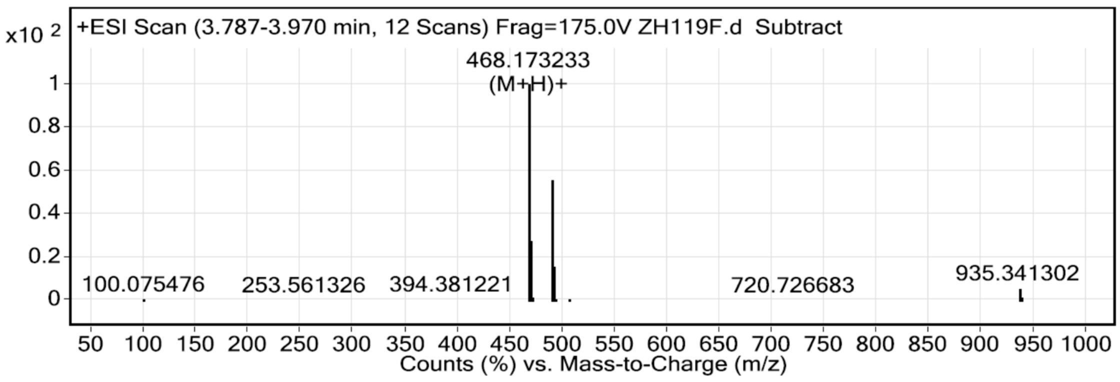

2-morpholino-*N*-(4-(3-(3-(4-(trifluoromethoxy)phenyl)ureido)phenyl)thiazol-2-yl)acetamide (**20**)

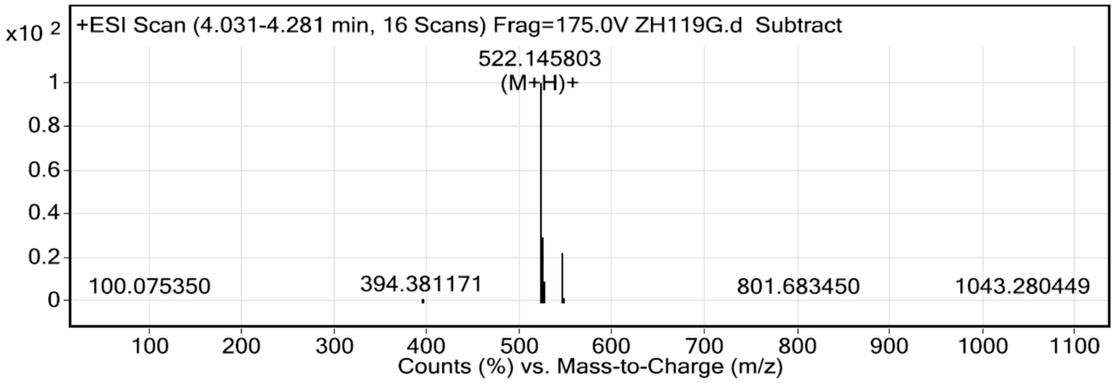

N-(4-(3-(3-benzylureido)phenyl)thiazol-2-yl)-2-morpholinoacetamide (**21**)

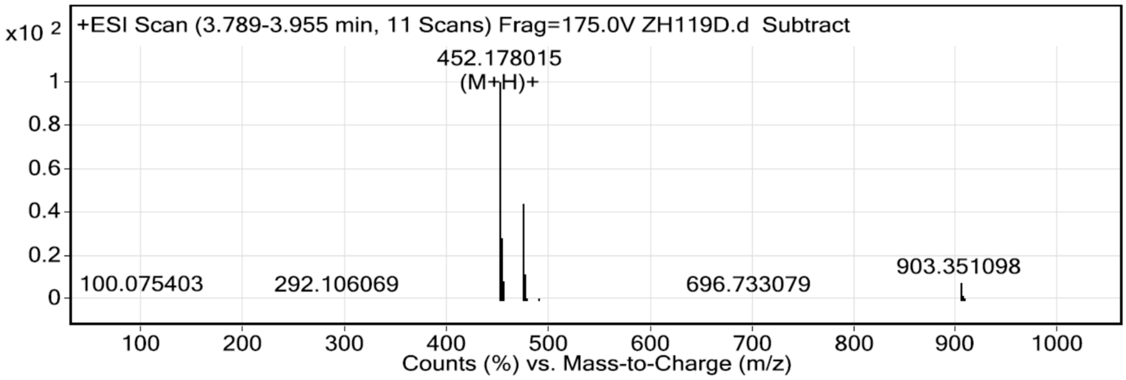

N-(4-(3-(3-(4-chlorophenyl)ureido)phenyl)thiazol-2-yl)-2-(piperidin-1-yl)acetamide (**22**):

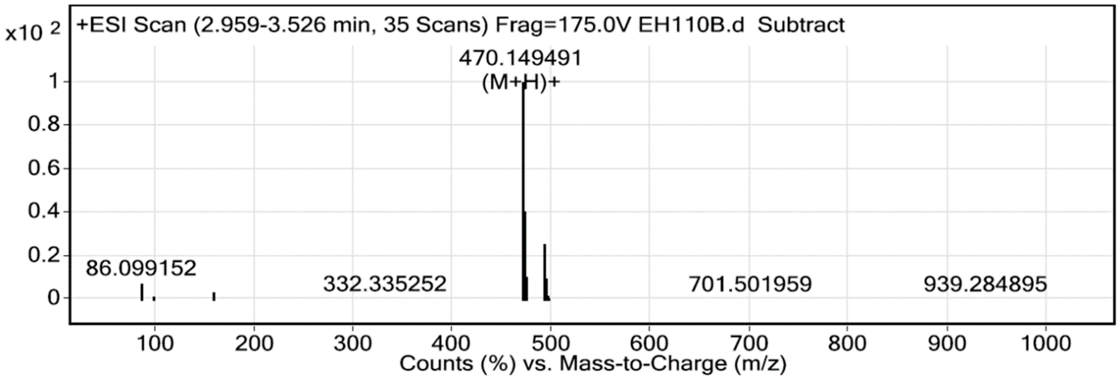

N-(4-(3-(3-(3-chlorophenyl)ureido)phenyl)thiazol-2-yl)-2-(piperidin-1-yl)acetamide (**23**):

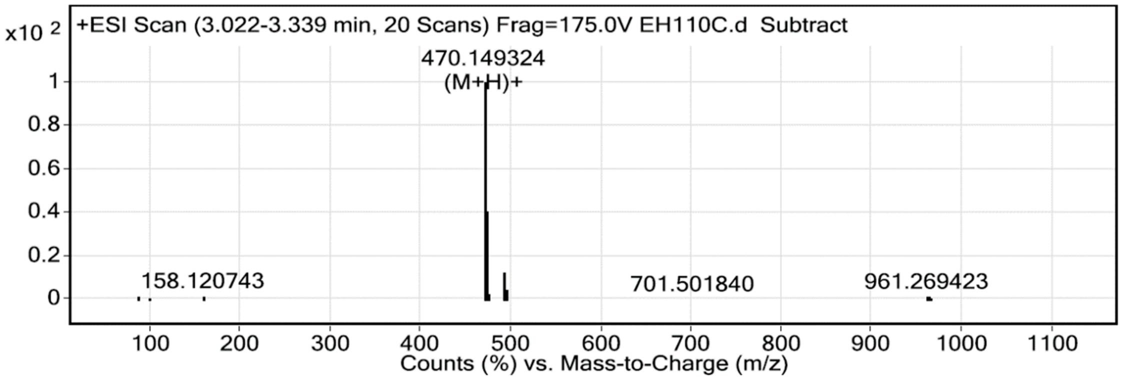

N-(4-(3-(3-(2,4-dichlorophenyl)ureido)phenyl)thiazol-2-yl)-2-(piperidin-1-yl)acetamide (**24**):

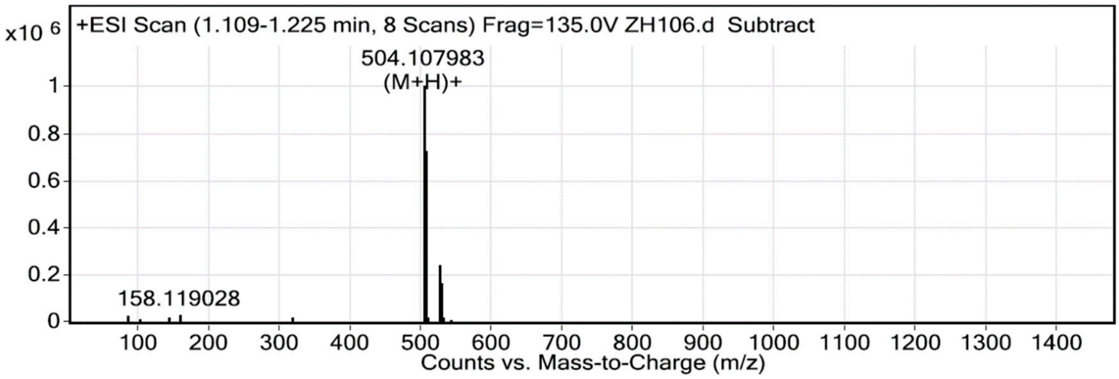

N-(4-(3-(3-(3,5-dichlorophenyl)ureido)phenyl)thiazol-2-yl)-2-(piperidin-1-yl)acetamide (**25**):

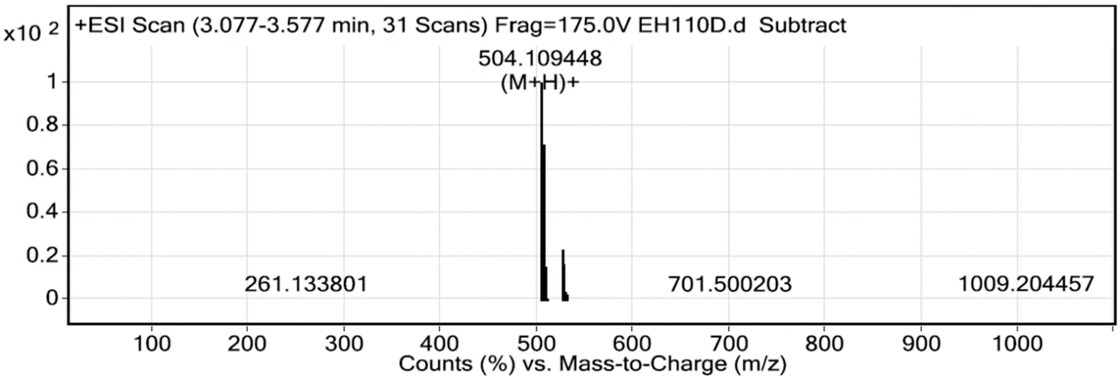

N-(4-(3-(3-(3-chloro-4-methylphenyl)ureido)phenyl)thiazol-2-yl)-2-(piperidin-1-yl)acetamide (**26**):

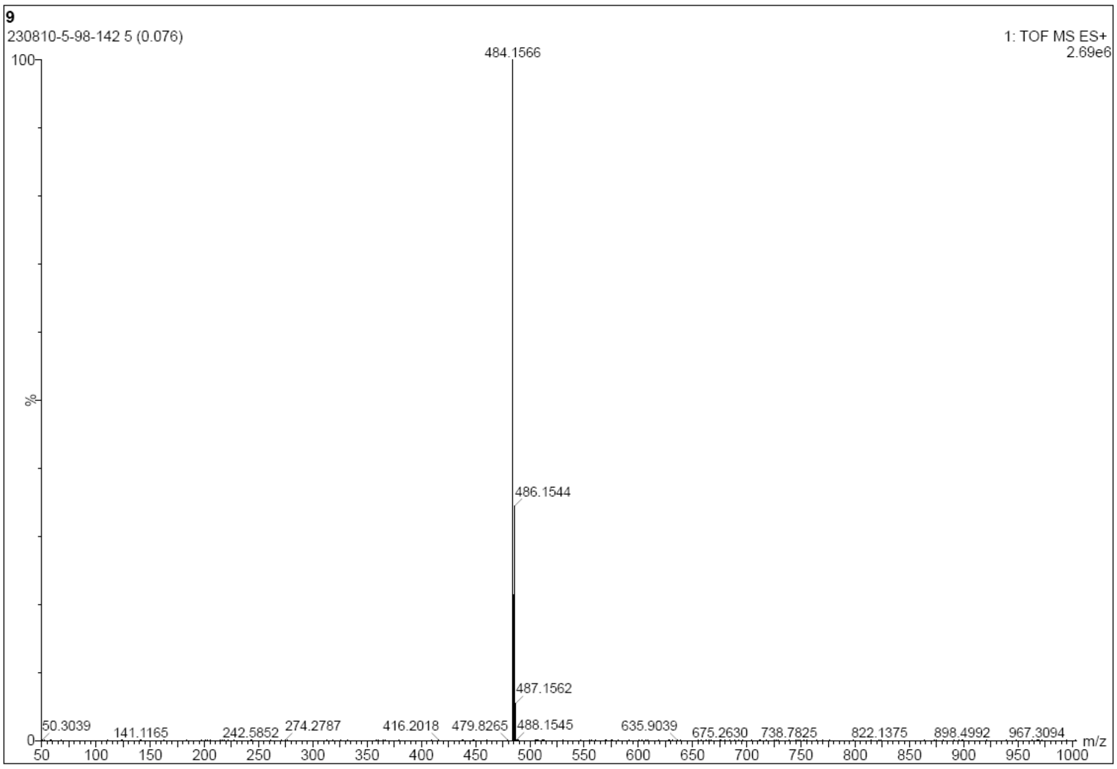

N-(4-(3-(3-(4-bromophenyl)ureido)phenyl)thiazol-2-yl)-2-(piperidin-1-yl)acetamide (**27**):

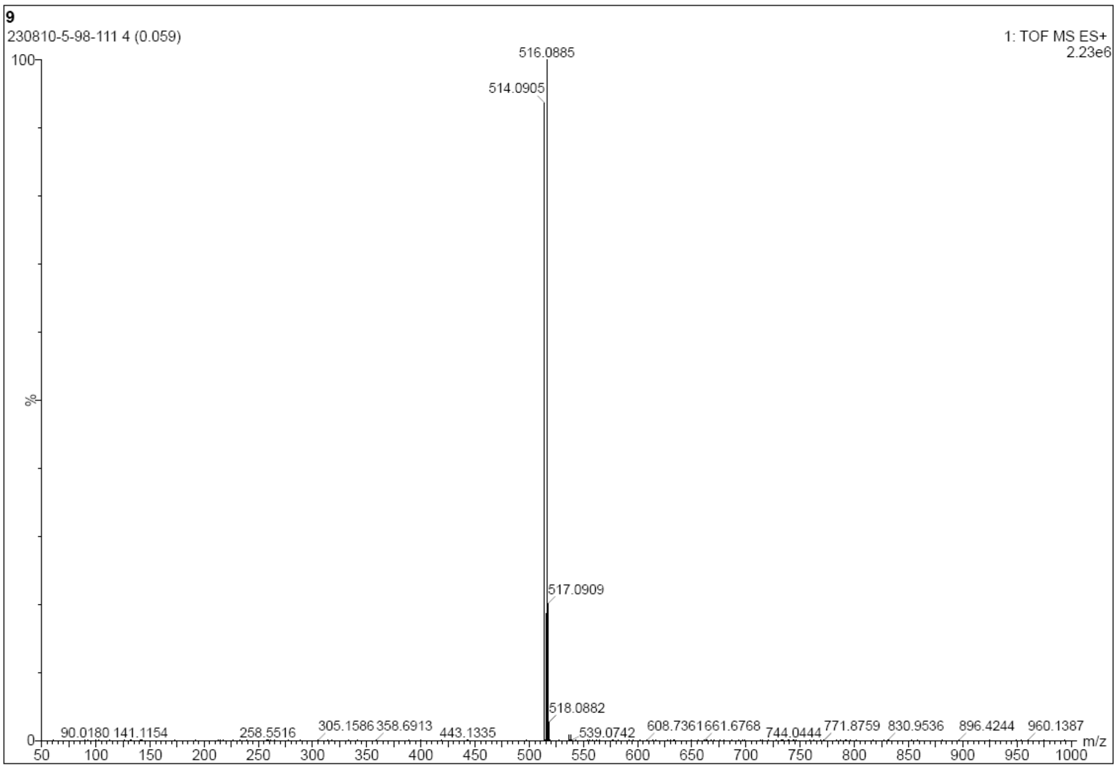

2-(piperidin-1-yl)-*N*-(4-(3-(3-(4-(trifluoromethyl)phenyl)ureido)phenyl)thiazol-2-yl)acetamide (**28**):

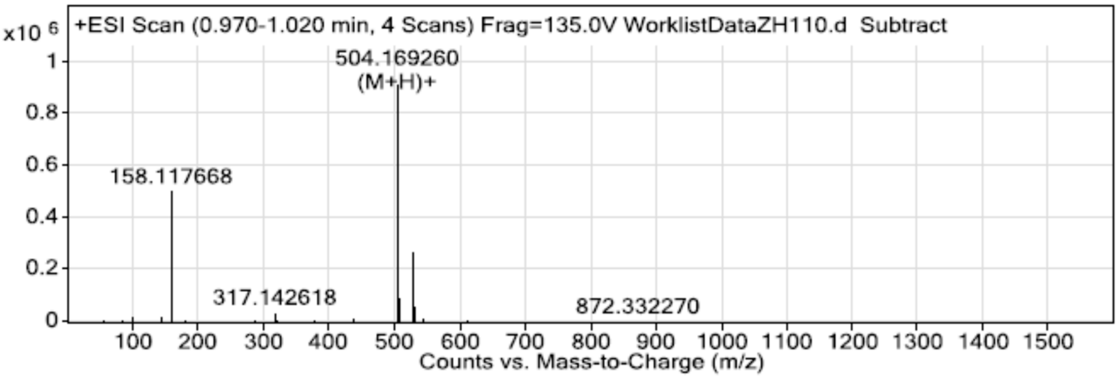

N-(4-(3-(3-(4-chloro-3-(trifluoromethyl)phenyl)ureido)phenyl)thiazol-2-yl)-2-(piperidin-1-yl)acetamide (**29**)

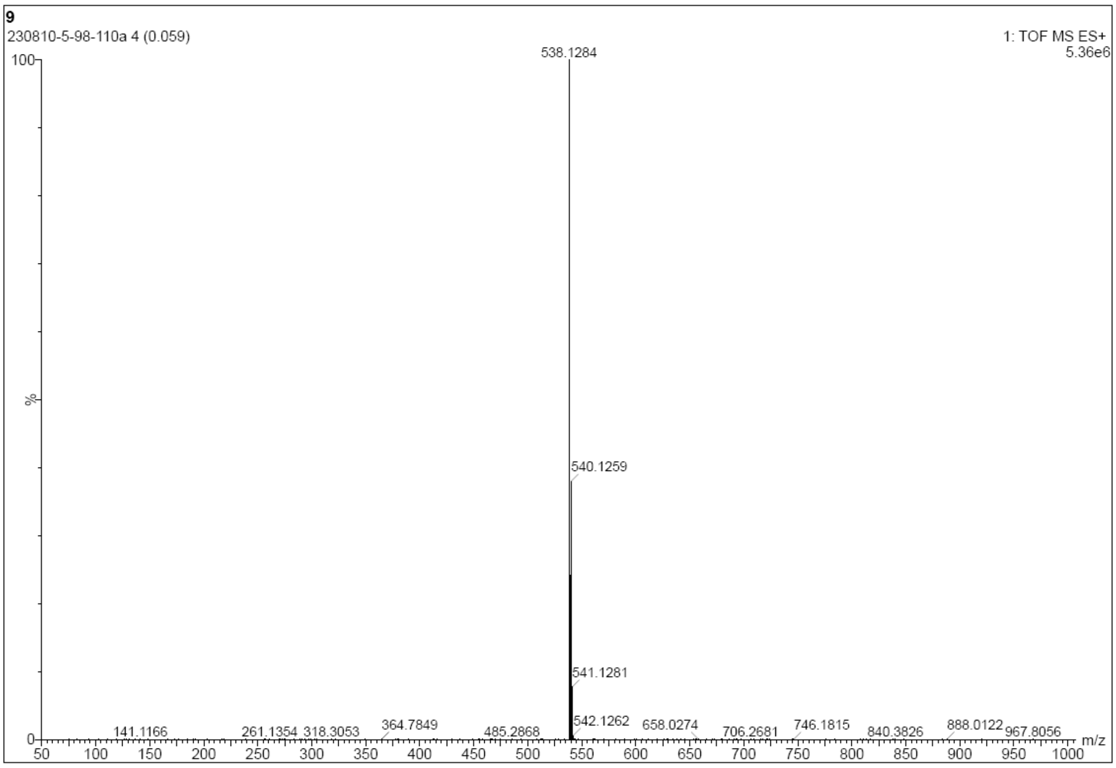

N-(4-(3-(3-(3-methoxyphenyl)ureido)phenyl)thiazol-2-yl)-2-(piperidin-1-yl)acetamide (**30**):

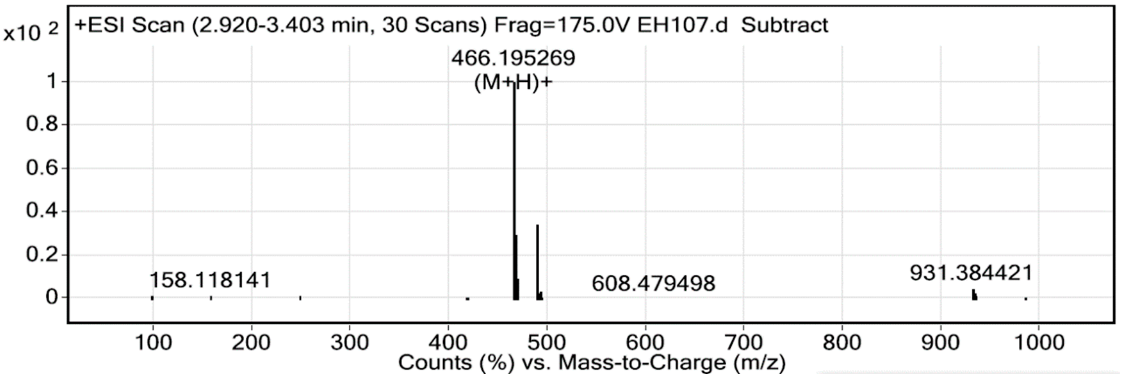

3. Bioinformatics analysis of potential targets for the target compound

3.1 Integration of Liver Cancer-Related Targets

The dataset "Adult Liver Carcinoma, C0220630" from DisGeNET yielded 1377 unique liver cancer-associated genes post-duplicate removal. Analysis of the human liver cancer gene chip dataset (GSE136846) identified 3807 significantly differentially expressed genes (PValue < 0.05, |log2 Fold Change| > 1).

Merging predictions from SwissTargetPrediction and Super-PRED revealed potential targets for compounds **13**, **20**, **21**, **25**, **27**, and **28**. UniProt IDs were converted to gene names (513 genes) using the UniProt website. Utilizing a Venn diagram, the intersection of targets produced 34 potential targets (see Figure. S-1).

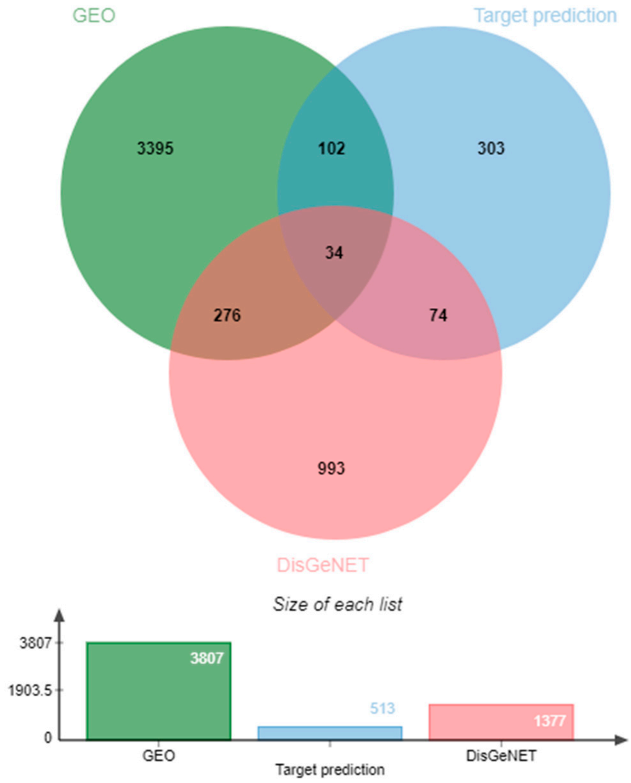

Figure S-1. The overlap between the potential targets of the representative target compounds and the key therapeutic targets for liver cancer.

3.2 Protein–Protein Interaction (PPI) Network Analysis

The 34 common target genes were input into the STRING database to construct a protein–protein interaction (PPI) network, illustrating the regulatory relationships between these targets. As depicted in Figure S-2, this PPI network was visualized using Cytoscape 3.10.1, consisting of 34 nodes and 140 edges. The average node degree was 8.24, and the average local clustering coefficient was 0.784. Importantly, the node size positively correlated with the degree of the liver cancer protein targets. Network analysis identified the top ten genes by degree—BCL2, PPARG, MMP9, PARP1, AR, PTGS2, MMP2, MTOR, CCNB1, and CXCR4—as potential key players in the development of liver cancer.

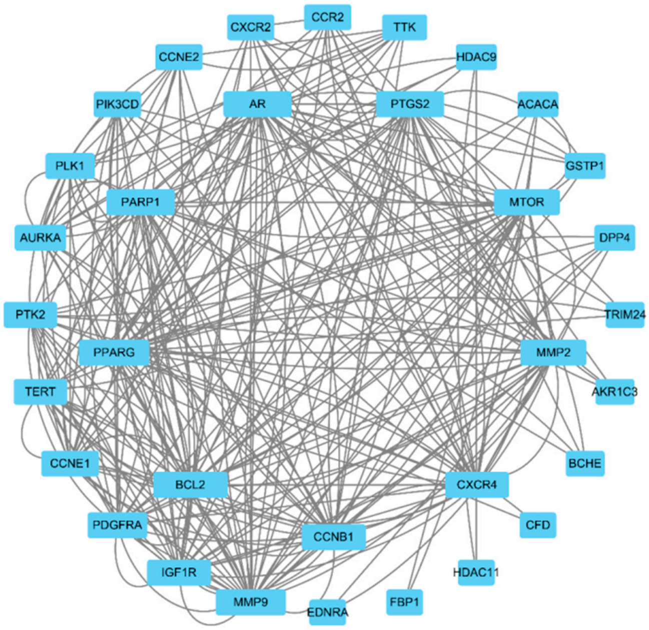

Figure S-2. Protein–protein interaction (PPI) network.

3.3 GO and KEGG Enrichment Analysis

The top ten genes set was subjected to GO (Gene Ontology) and KEGG (Kyoto Encyclopedia of Genes and Genomes) enrichment analysis using the R language. A functional analysis was conducted from three perspectives: Biological Processes (BPs), Cellular Components (CCs), and Molecular Function (MF). A total of 216 significant GO entries ($P < 0.05$) were obtained, comprising 157 in BPs, 25 in CCs, and 34 in MF. The top 10 entries from each category were selected for bar graph representation, as illustrated in Figure S-3.

The GO enrichment analysis of liver cancer-related genes revealed their crucial roles in diverse biological processes, cellular components, and molecular functions. These genes actively participate in responses to peptide hormones, hypoxia, nutrient levels, cell movement, and stress responses. At the cellular level, they function in vital structures like transferase complexes, chromosomal regions, and spindles. Additionally, the genes exhibit essential molecular functions such as interacting with DNA-binding transcription factors, protein tyrosine kinase activity, and chemokine receptor functions. These findings emphasize the compounds' potential in targeting key pathways, providing a foundation for effective liver cancer treatments.

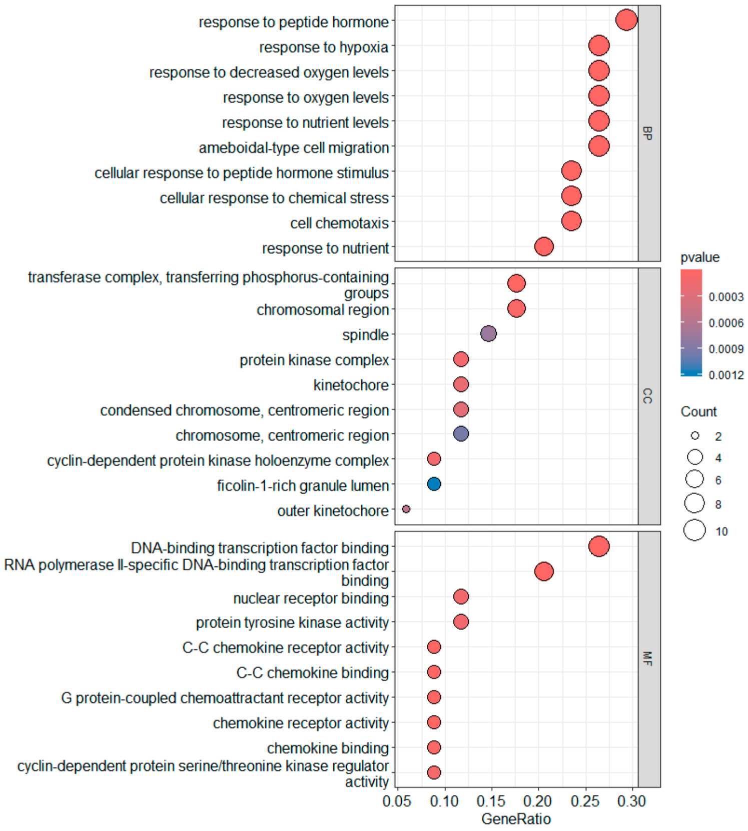

Figure S-3. The top 10 significant GO enrichment results for liver cancer-related genes.

As illustrated in Figure S-4, the KEGG enrichment analysis reveals pivotal pathways covering diverse biological processes and disease-related pathways. Noteworthy pathways include Prostate Cancer (10 associated genes), MicroRNAs in Cancer (8 genes), Endocrine Resistance (7 genes), Oocyte Meiosis (7 genes), Human Cytomegalovirus Infection, Small Cell Lung Cancer (6 genes), the AMPK Signaling Pathway (6 genes), Fluid Shear Stress and Atherosclerosis (6 genes), Gastric Cancer (6 genes), and EGFR Tyrosine Kinase Inhibitor Resistance (5 genes). These significantly enriched pathways span multiple facets of cancer, encompassing signaling cascades, cell cycle modulation, immune regulation, and infectious diseases related to cancer.

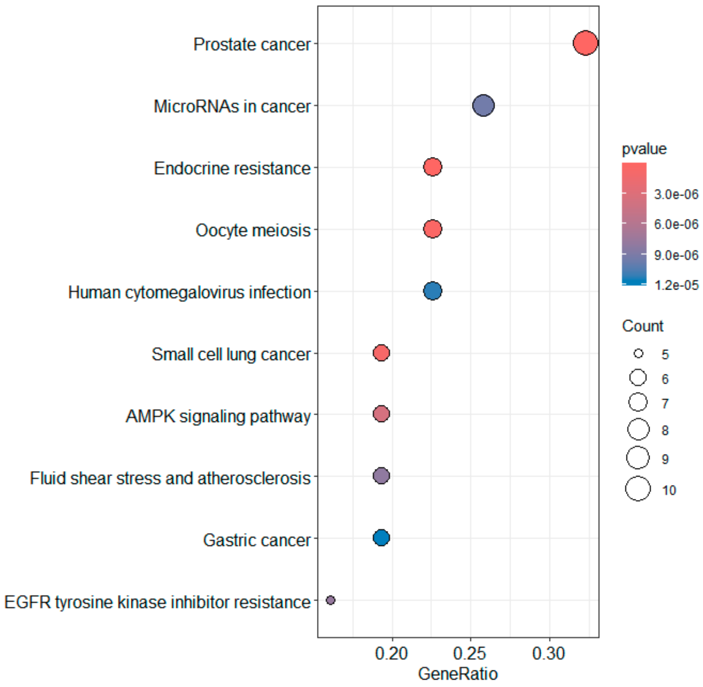

Figure S-4. The top 10 significant KEGG enrichment results for liver cancer-related genes.

3.4 KEGG pathway analysis

As depicted in Figure S-5, the 34 intersecting target genes were precisely mapped onto relevant pathways using Mapper (https://www.kegg.jp/kegg/mapper/search.html). In the Human Hepatocellular Carcinoma pathway (KEGG ID: hsa05225), five pivotal genes were identified, namely, the mechanistic targets of MTOR, GSTP1, IGF1R, PIK3CD, and TERT. These genes play crucial regulatory roles in the pathogenesis of hepatocellular carcinoma, unveiling potential molecular mechanisms underlying this disease. Specifically, IGF1R is an FDA-approved anti-tumor target.

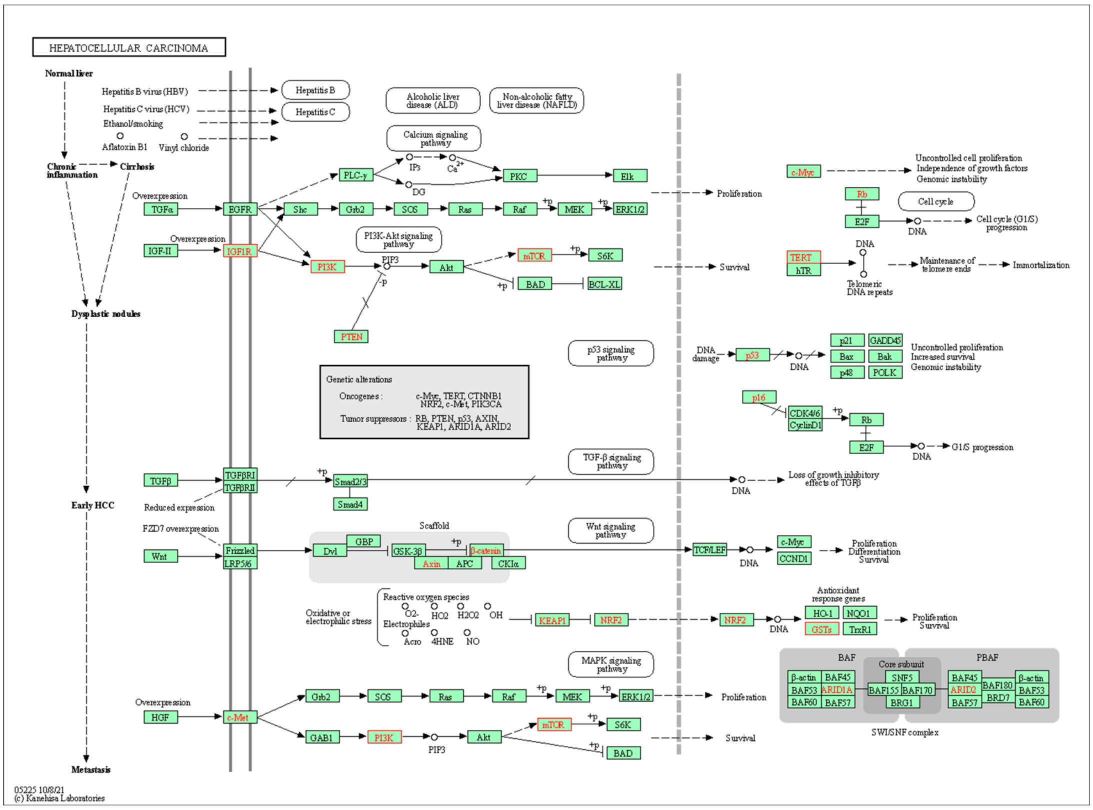

Figure S-5. KEGG Hepatocellular carcinoma—Homo sapiens (human).

4. Network Pharmacology Steps

4.1 Selection of Liver Cancer-Related Targets

Utilizing the DisGeNET database (https://www.disgenet.org/search), a search was conducted using the keywords "liver cancer" or "hepatic carcinoma," and "Adult Liver Carcinoma, C0220630" was chosen as the target. Key gene targets associated with adult liver carcinoma were identified, and duplicate genes were excluded.

Accessing the Gene Expression Omnibus (GEO) database (https://www.ncbi.nlm.nih.gov/geo), gene chip datasets related to human liver cancer were downloaded, ensuring the species was Homo sapiens. The online tool "Analyze with GEO2R" was employed to analyze and select differentially expressed genes (DEGs) between human liver cancer and adjacent normal tissues. The criteria for DEG selection were a log |log2 Fold Change| > 1.0 and a significant PValue < 0.05.

4.2 Reverse Prediction of Potential Target

Accessing SwissTarget Prediction (http://www.swisstargetprediction.ch/) and Super-PRED (https://prediction.charite.de/subpages/target_prediction.php), SMILES strings of representative compounds were obtained using PubChem (https://pubchem.ncbi.nlm.nih.gov/). Subsequently, the SMILES strings were input into the mentioned websites for virtual screening.

4.3 Construction of Intersection of Target Compounds and Disease-Related Targets

Predicted targets of representative compounds were obtained, and the target genes, converted into UniProt IDs, were intersected with disease targets selected from other websites. This intersection of genes was obtained using the MicroBioNet online bioinformatics analysis

and visualization platform (https://www.bioinformatics.com.cn/).

Construction of Protein–Protein Interaction Network (PPI): The intersection genes were uploaded to the STRING database (https://stringdb.org), selecting the species as "Homo sapiens" and removing isolated targets. Subsequently, "Send network to Cytoscape" was executed, optimizing the interaction network diagram between targets in Cytoscape 3.10.1 (**https://cytoscape.org/).**

4.4 GO and KEGG Enrichment Analysis

GO and KEGG Enrichment: Using the R language, an enrichment analysis of GO biological processes and KEGG metabolic pathways was conducted for the intersected genes from the Venn diagram. In this analysis, the org.Hs.eg.db data object was imported, incorporating data from major databases, providing the entrez ID and ensembl database ID. This study aims to delve into the biological processes and signaling pathways involved in the gene set.

4.5 KEGG Pathway Analysis

The primary function of Mapper (https://www.kegg.jp/kegg/mapper/search.html) is to accurately locate genes within relevant pathways, enabling researchers to explore the functional interactions of genes in biological pathways in greater detail.

4.6 KEGG Pathway Analysis

Visit the KEGG database website (https://www.kegg.jp/) and find the pathway search or Mapper tool. Enter the names of intersection genes in the search box to perform a gene pathway association analysis, precisely locating these genes in relevant KEGG pathways.

5. Drug-likeness prediction

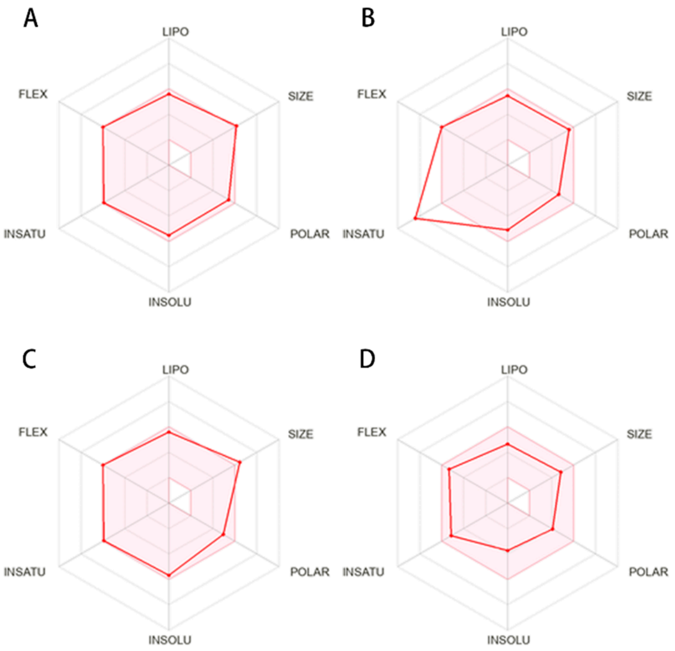

Figure S-6. Bioavailability radar chart generated by Swiss-ADME for (A) compound **27**, (B) Sorafenib, (C) Nintedanib, and (D) Sunitinib.

Table S-1. Drug-likeness evaluation of compound **27** compared with Sorafenib, Nintedanib, and Sunitinib.

Test items	compound 27	Sorafenib	Nintedanib	Sunitinib
Water Solubility				
Log S (ESOL)	-5.53	-5.11	-5.69	-3.72
Solubility (mol/L)	2.94×10^{-6}	7.79×10^{-6}	2.05×10^{-6}	1.90×10^{-4}
Class	Moderate	Moderate	Moderate	Soluble
Log S (Ali)	-6.41	-5.71	-6.17	-3.90
Solubility (mol/L)	3.89×10^{-7}	1.93×10^{-6}	7.17×10^{-7}	1.25×10^{-4}
Class	Moderate	Moderate	Poor	Soluble
Log S (SILICOS-IT)	-8.17	-8.60	-7.93	-7.35
Solubility (mol/L)	6.81×10^{-9}	2.50×10^{-9}	1.17×10^{-8}	4.43×10^{-8}
Class	Poor	Poor	Poor	Poor
Pharmacokinetics				
GI absorption	High	Low	High	High
BBB permeant	No	No	No	Yes
P-gp substrate	No	No	Yes	Yes
CYP1A2 inhibitor	No	Yes	No	No
CYP2C19 inhibitor	Yes	Yes	Yes	Yes
CYP2C9 inhibitor	Yes	Yes	Yes	No
CYP2D6 inhibitor	Yes	Yes	Yes	Yes
CYP3A4 inhibitor	Yes	Yes	No	Yes
Log K_p (cm/s) (skin permeation)	-6.39	-6.25	-6.54	-6.86
Drug-likeness				
Lipinski	Yes<br>1 violation: MW>500	Yes<br>0 violations	Yes<br>1 violation: MW>500	Yes<br>0 violations
Ghose	No<br>2 violations: MW>480, MR>130	No<br>1 violation: WLOGP>5.6	No<br>3 violations: MW>480, MR>130, #atoms>70	Yes
Veber	Yes	Yes	Yes	Yes
Egan	Yes	No<br>1 violation: WLOGP>5.88	Yes	Yes
Muegge	Yes	Yes	Yes	Yes
Bioavailability Score	0.55	0.55	0.55	0.55
Medicinal Chemistry				
PAINS	0 alert	0 alert	0 alert	0 alert
Brenk	0 alert	0 alert	1 alert<br>imine_1	1 alert<br>michael_acceptor_1
Lead-likeness	No<br>3 violations: MW>350, Rotors>7, XLOGP3>3.5	No<br>3 violations: MW>350, Rotors>7, XLOGP3>3.5	No<br>3 violations: MW>350, Rotors>7, XLOGP3>3.5	No<br>2 violations: MW>350, Rotors>7
